# MicroRNA cluster miR199a/214 are differentially expressed in female and male rats following nicotine self-administration

**DOI:** 10.1038/s41598-018-35747-z

**Published:** 2018-11-30

**Authors:** Steven T. Pittenger, Victoria L. Schaal, Dalia Moore, Rahul S. Guda, Sneh Koul, Sowmya V. Yelamanchili, Rick A. Bevins, Gurudutt Pendyala

**Affiliations:** 1 Department of Psychology, University of Nebraska-Lincoln, Lincoln, Nebraska USA; 2 Department of Pharmacology and Experimental Neuroscience, University of Nebraska Medical Center, Omaha, Nebraska USA; 3 Department of Anesthesiology, University of Nebraska Medical Center, Omaha, Nebraska USA; 4 Present Address: Yale University School of Medicine, Division of Molecular Psychiatry, New Haven, Connecticut USA

## Abstract

Previous research has established sex differences associated with nicotine intake, however a significant gap in knowledge remains regarding the molecular mechanisms that govern these differences at the transcriptional level. One critical regulator of transcription are microRNAs (miRNAs). miRNAs are a family of non-coding RNAs that regulate an array of important biological functions altered in several disease states, including neuroadaptive changes within the brain associated with drug dependence. We examined the prefrontal cortex (PFC) from male and female Sprague-Dawley rats following self-administration (22 days) of nicotine or yoked saline controls using next generation RNA-Sequencing (RNA-Seq) technology and found an array of miRNAs to be significantly and differentially regulated by nicotine self-administration. Of these, we found the expression of miR-199a and 214, which are expressed on the same cluster of chromosome 1, to be upregulated in the female rats exposed to nicotine; upregulation in this group was further validated by real time polymerase chain reaction (RT-PCR). Bioinformatics analysis to assess common targets of miR-199/214 identified Sirtuin 1 (SIRT1), a nicotinamide adenine dinucleotide (NAD)- dependent deacetylase that plays a role in apoptosis, neuron survival, and stress resistance. Using western-blot, we confirmed downregulation of SIRT1 and increased cleaved caspase 3 expression in the brains of nicotine-exposed female rats and no change in expression levels in the other groups. Collectively, our findings highlight a miR-199/214 regulatory network that, through SIRT1, may be associated with nicotine seeking in females which may serve as a potential therapeutic target for sex-specific treatment approaches.

## Introduction

Smoking remains a significant health and economic burden in the United States, resulting in 480,000 deaths and costing more than $300 billion annually^1^. While many smokers report a desire to quit, most are unsuccessful in quit attempts^2^. The tenacity of this addiction can be, in part, attributed to nicotine, the primary addictive constituent in cigarettes^3^. Notable sex differences related to smoking and quitting have been identified [for a review see^4^]. While men are more likely to be smokers and smoke more cigarettes per day, women develop dependence to nicotine more rapidly. Women also have higher relapse rates than men and nicotine replacement therapy may be less effective^4–6^. These differences may reflect women’s greater sensitivity to non-pharmacokinetic effects of nicotine^4,7^.

While environmental and social factors likely play a role in some of these nicotine sex differences, biological factors including the effect of gonadal hormones on nervous system development, changes in circulating gonadal hormones across the lifespan, and differential expression of X and Y genes have also been implicated^8–12^. These factors produce well-described sex differences in the bio-behavioral processes that contribute to drug addiction–learning and memory, drug efficacy and toxicity, neuroinflammation, etc.^13–16^. Concomitantly, sex differences in pre-clinical animal models have also been demonstrated. Females rats show greater nicotine-induced locomotor sensitization, more pronouced withdrawal following nicotine cessation, enhanced conditoned reinforcement to nicotine-associated cues, and higher break points on a progressive ratio nicotine self-administratin schedule^4,17–19^. Previous studies have also demonstrated that endogenous neuropeptide experession is sex-dependently altered following nicotine self-administration^20^. Tissue levels of neurotensin in the PFC were increased in the male, but not female, rats following nicotine self-administration compared to saline controls, implicating the PFC as an area of interest when exploring sex differences related to nicotine intake^20^.

While nicotine sex differences have been established, a significant gap in knowledge remains regarding the molecular mechanisms that govern these differences at the transcriptional level. One critical regulator of transcription are microRNAs (miRNAs), which are a family of non-coding RNAs. At the molecular level miRNAs are about 21–23 nucleotides long and regulate gene expression through direct binding to the 3′ untranslated region (UTR) of gene targets, thereby inhibiting their translation into protein^21^. From a pathological perspective, miRNAs have been shown to regulate an array of important biological functions in several diseases including neuroadaptive changes within the brain caused by drugs of abuse^22–24^.

Given the effects of nicotine on brain areas involved in emotional and cognitive processing, we performed RNA-Seq on the prefrontal cortex (PFC) from female and male rats following long-term nicotine self-administration. The PFC was selected as it is critically involved in higher-order executive function, as well as regulation of behaviors associated with drug abuse and withdrawal in humans^25–27^ and non-human animals^28–30^. PFC involvement in the modulation of maladaptive behaviors associated with stimulant dependence is well supported, however little is known regarding differential PFC miRNAs expression between sexes in a preclinical animal model of nicotine taking (i.e., self-administration). Using RNA-Seq technology, which enables rapid and quantitative profiling of the transcriptome, we profiled the PFC of male and female Sprague Dawley rats that self-administered nicotine (0.0073 mg/infusion) or received yoked saline infusions for 22 days on a variable ratio 3 (VR3) paradigm to identify differentially expressed miRNAs. Nicotine self-administration in the females had a more notable effect compared to the males. Among the differentially expressed miRNAs, miR-199a and 214, which are both expressed on the same cluster of chromosome 1, were upregulated in the female rats self-administering nicotine compared to males. This was validated by real time polymerase chain reaction (RT-PCR). Further, bioinformatics analysis to assess common targets of miR-199/214 identified Sirtuin 1 (SIRT1), a nicotinamide adenine dinucleotide (NAD)- dependent deacetylase that plays a role in apoptosis, neuron survival, stress resistance, and regulation of drug dependence^31–34^. Using western-blot, we confirmed expression of SIRT1 was only downregulated in the brains of nicotine exposed female rats, with no expression change in the males. To our knowledge, this is the first study to employ nicotine self-administration paradigm coupled with next generation sequencing technology to identify sex-specific miRNA signatures associated with long term nicotine self-administration. Collectively, our findings highlight a miR-199/214 regulatory network via downregulation of SIRT1 to be associated with nicotine self-administration in females but not males.

## Results

### Nicotine maintained robust self-administration

**Figure 1:**
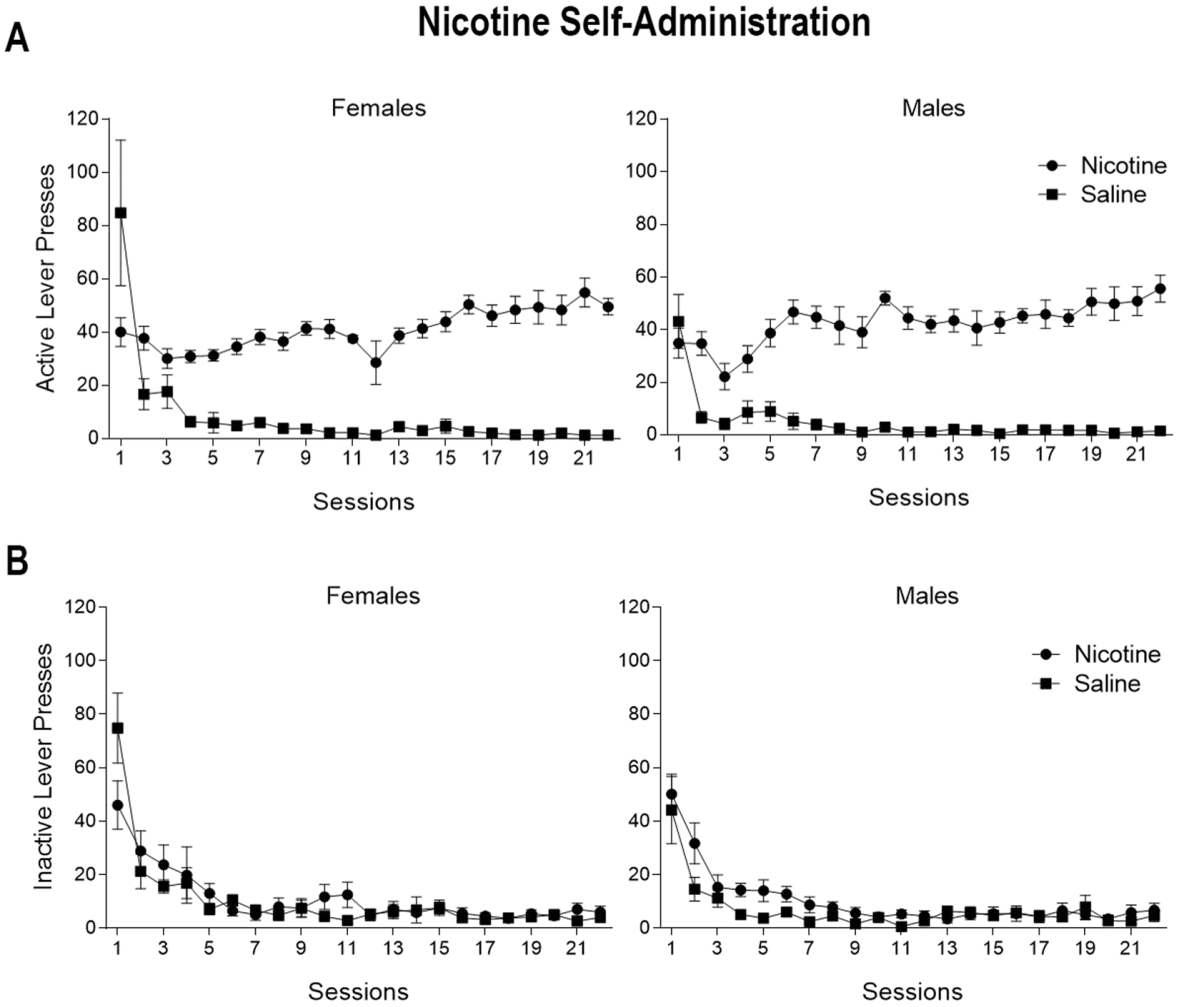
Nicotine maintained robust self-administration. (**A**) Active lever presses for females (left panel) and males (right panel) receiving nicotine infusions on a VR3 schedule of reinforcement (circles) or yoked-saline infusions (squares). Yoked-saline infusions were administered contingent on responding from a nicotine partner. That is, yoked rats did not control the saline infusions. (**B**) Inactive lever presses for females (left panel) and males (right panel) receiving nicotine infusions on a VR3 schedule of reinforcement (circles) or yoked-saline infusions (squares).

Both male and female rats maintained robust self-administration of nicotine on a VR3 schedule over the 22 sessions, while yoked-saline maintained very little responding (Fig. 1A). For active lever presses, there was a main effect of Group [F(1,21) = 410.25, p < 0.01] and Session [F(21,441) = 8.97, p < 0.01] but no effect of Sex (F < 1, p = 0.66). The 3-way Sex x Group x Session interaction [F(21, 441) = 1.03, p = 0.42] and the 2-way Sex x Group interaction [F(1,21) = 2.28, p = 0.14] were not significant. The Group x Session interaction was significant [F(21, 441) = 15.00, p < 0.01]. Saline-yoked rats responded more on session 1, but the nicotine rats responded more on sessions 2 to 22. The Sex x Session interaction was also significant [F(21,441) = 2.30 p < 0.01]; the female rats responded more than males on sessions 1 and 3 with equivalent responding on all other sessions. Responding on the inactive lever quickly decreased (Fig. 1B), demonstrating a clear discrimination between the active and inactive lever in male and female nicotine rats. For inactive lever presses, there was a main effect of Session [F(21,441) = 39.01, p < 0.01], but no main effect of Sex [F(1,21) = 1.79, p = 0.19] or Group [F(1,21) = 1.89, p = 0.18]. The 3-way interaction of Sex x Group x Session [F(21,441) = 1.49, p = 0.07], the 2-way interaction of Sex x Session [F(21,441) = 1.04, p = 0.40] and the 2-way interaction of Sex x Group (F < 1, p = 0.43) were not significant. There was a significant interaction of Group x Session [F(21,441) = 1.66, p < 0.05], with saline-yoked rats responding more on the inactive lever on session 1 and the nicotine rats responding more on the inactive lever on sessions 2, 5, and 11.

**Figure 2:**
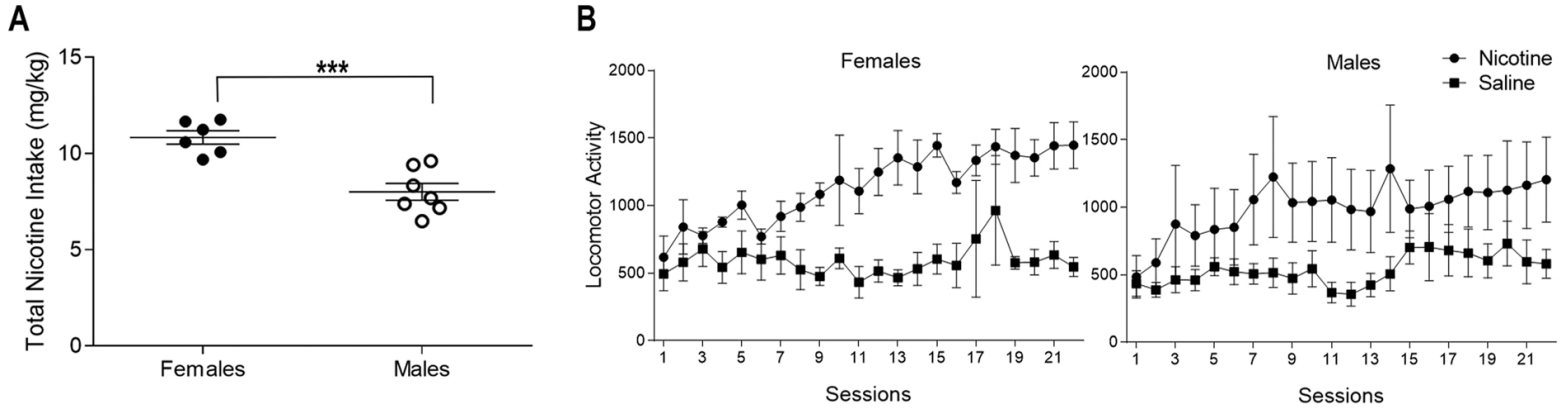
Nicotine intake and locomotor activity. (**A**) Total nicotine intake throughout self-administration is displayed for female (closed circle) and male (open circle) rats. (**B**) Locomotor activity for females (left panel) and males (right panel) receiving nicotine infusions on a VR3 schedule of reinforcement (circles) or yoked-saline infusions (squares).

Interestingly, when differences in body weight were accounted for, males and females did differ in total nicotine intake [Fig. 2; t(11) = 4.886 p < 0.001]. Females (10.83 ± 0.35 mg/kg) had greater total nicotine intake than the males (8.01 ± 0.44 mg/kg) (Fig. 2A) when intake over all 22 days are summed. Male and female nicotine rats displayed an increase in general locomotor activity (Fig. 2B). There were main effects of Group [F(1,21) = 7.12, p < 0.05] and Session [F(21,441) = 6.21, p < 0.01] but no main effect of Sex (F < 1, p = 0.60). The 3-way Sex x Group x Session [F(21,441) = 1.07, p < 0.37], the 2-way Sex x Group (F < 1, p = 0.81), and the 2-way Sex x Session (F < 1, p = 0.68) interactions were not significant. There was a Group x Session interaction [F(21,441) = 3.26, p < 0.01]; the nicotine rats showed more general locomotor activity than the saline-yoked rats on sessions 2 to 22.

### Differential expression of miRNAs post nicotine exposure

**Figure 3:**
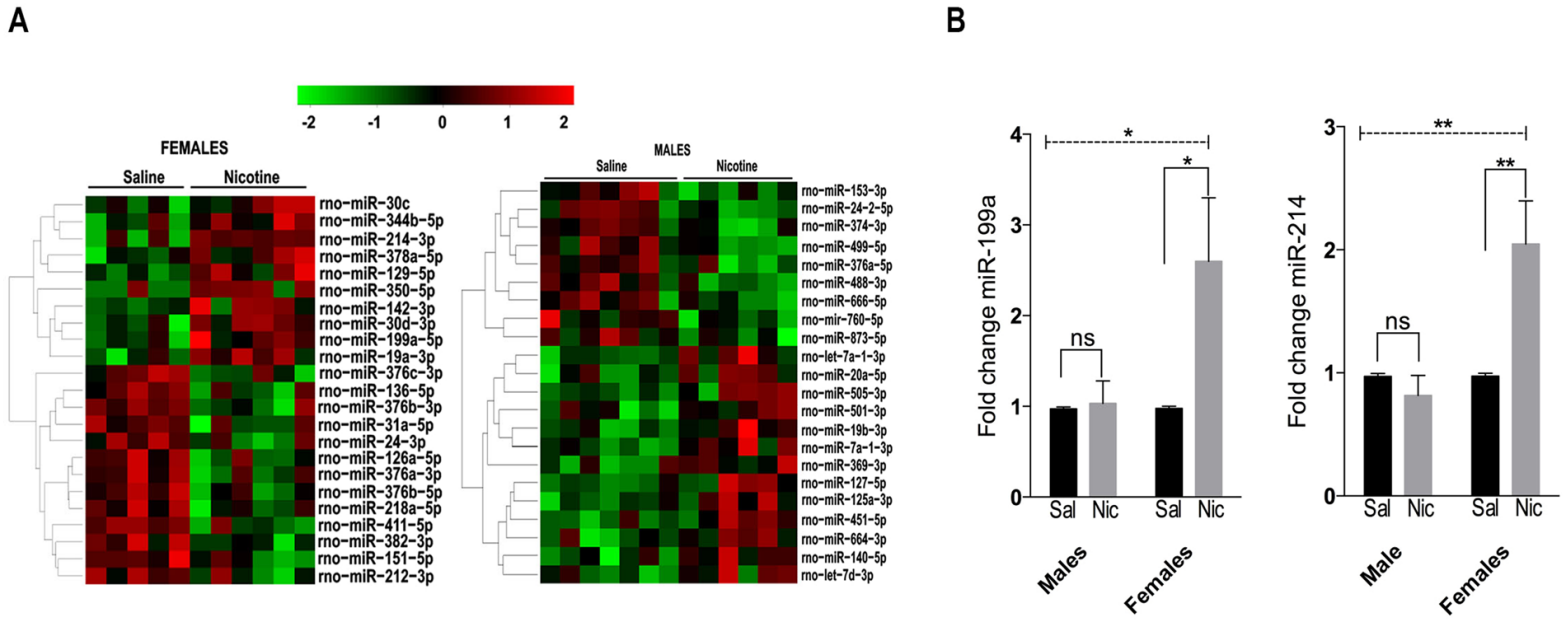
Differentially expressed miRNAs and post validation. (**A**) Venn diagram showing differentially expressed miRNA between males and females post nicotine self-administration. The color code represents the expression levels (Red- up and Green – down). (**B**) Validation of the increase in expression of miR-199a and miR-214 in female rats that self-administered nicotine compared to saline controls. Overall 2-Way ANOVA (hatched line) with Sidak’s multiple comparison test (*p < 0.05 and **p < 0.01) was used to determine significance. ns- not significant.

Following completion of the 22 self-administration sessions, total PFC RNA isolated from individual animals was subjected to miRNA sequencing using an RNA-Seq approach to identify differential miRNAs as sex-specific signatures associated with chronic nicotine exposure. We obtained between 5 and 34 million raw reads per sample per condition and observed an overall greater than 83% of mapped reads between the sexes (Supplemental Table 1). Among the 688 miRNAs profiled (Supplemental Table 2), we identified 45 miRNAs, 23 in females and 22 in males, to be differentially expressed after nicotine self-administration (Fig. 3A). Amongst the 23 miRNAs in females, 10 were up regulated and 13 down regulated while in the males, 9 were up regulated and 13 down regulated (Table 1). Interestingly, the overall levels of expression of the differentially expressed miRNAs were more subtle in the males post nicotine intake. Females, on the contrary, showed enhanced and robust expression of some miRNAs. Further applying a cutoff of ±1.5 fold change and p < 0.05 as our criteria, we found four miRNAs to be up regulated in the females post nicotine intake: miR-214-3p (+7.94 fold), miR-350-5p (+4.44), miR-19a-3p (+2.14), and miR-199a-5p (+1.77). For the males, only miR-451-5p (+1.69) was up regulated. None of the down regulated miRNAs met criteria in the females. However, miR-760-5p (−1.58 fold) was down regulated in the males after nicotine exposure.

**Table 1:** Differential miRNAs expression analysis post nicotine self-administration between males and females (− sign before the number indicates down regulation).

Females			Males		
miRNA	Fold Change	p-value	miRNA	Fold Change	p-value
rno-miR-214-3p	7.94	4.28E-02	rno-miR-451-5p	1.69	2.41E-02
rno-miR-350-5p	4.44	4.07E-02	rno-miR-19b-3p	1.35	1.85E-02
rno-miR-19a-3p	2.14	2.07E-02	rno-miR-127-5p	1.3	4.47E-02
rno-miR-199a-5p	1.77	2.62E-02	rno-miR-501-3p	1.28	3.63E-02
rno-miR-378a-5p	1.38	2.74E-02	rno-miR-125a-3p	1.25	3.81E-02
rno-miR-142-3p	1.29	3.08E-02	rno-miR-7a-1-3p	1.22	1.38E-02
rno-miR-344b-5p	1.26	3.98E-02	rno-let-7a-1-3p	1.21	2.17E-02
rno-miR-129-5p	1.2	1.10E-02	rno-miR-20a-5p	1.21	3.55E-02
rno-miR-30d-3p	1.17	1.85E-02	rno-miR-505-3p	1.21	3.82E-02
rno-miR-30c-5p	1.15	4.03E-02	rno-miR-664-3p	1.19	2.86E-02
rno-miR-376c-3p	−1.36	1.37E-02	rno-let-7d-3p	1.17	4.60E-02
rno-miR-151-5p	−1.33	1.55E-02	rno-miR-140-5p	1.16	1.61E-02
rno-miR-376a-3p	−1.33	2.77E-02	rno-miR-369-3p	1.16	3.93E-02
rno-miR-218a-5p	−1.3	2.32E-02	rno-mir-760-5p	−1.58	3.33E-02
rno-miR-376b-3p	−1.26	2.05E-02	rno-miR-374-3p	−1.39	3.74E-02
rno-miR-376b-5p	−1.25	1.21E-02	rno-miR-499-5p	−1.29	9.67E-03
rno-miR-382-3p	−1.23	1.47E-02	rno-miR-24-2-5p	−1.29	1.64E-02
rno-miR-126a-5p	−1.21	1.95E-02	rno-miR-666-5p	−1.23	4.32E-02
rno-miR-31a-5p	−1.21	4.09E-02	rno-miR-488-3p	−1.22	1.07E-02
rno-miR-411-5p	−1.19	6.27E-03	rno-miR-376a-5p	−1.19	3.52E-02
rno-miR-212-3p	−1.15	3.49E-02	rno-miR-873-5p	−1.18	3.73E-02
rno-miR-24-3p	−1.13	3.91E-02	rno-miR-153-3p	−1.13	2.06E-02
rno-miR-136-5p	−1.12	2.82E-02			

Notably, miRs-199 and 214, which are co-expressed on the same cluster of chromosome 1, was significantly up regulated in female rats exposed to nicotine compared to males that received nicotine. The up regulation of these miRNAs solely in females self-administering nicotine and not in the males was further validated by RT-PCR (Fig. 3B). For miR-199a, the 3-way interaction of Sex x Group x Session [F(1,20) = 4.437, p = 0.048], the 2-way interaction of Sex x Session [F(1,20) = 4.481, p = 0.047] and the 2-way interaction of Sex x Group [F(1,20) = 5.146 p = 0.035] were significant. Similarly, for miR-214 the the 3-way interaction of Sex x Group x Session [F(1,16) = 10.14, p = 0.058], the 2-way interaction of Sex x Session [F(1,16) = 10.16, p = 0.006] and the 2-way interaction of Sex x Group (F(1,16) = 5.686, p = 0.030) was significant.

### SIRT1 is a target of miR-199 and 214

**Figure 4:**
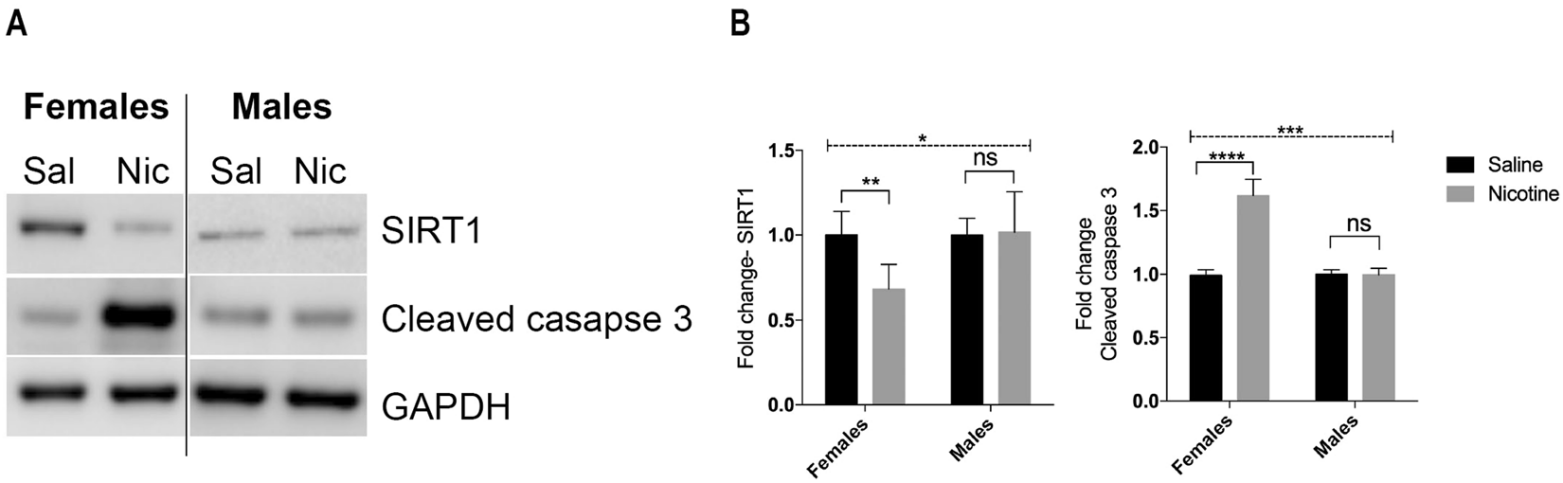
Validation of SIRT1 downregulation in female rats that self administered nicotine. (**A**) Representative western blot showing a decrease in SIRT1 expression in the brain lysates of female rats that self-administered nicotine compared to saline controls. This increase in SIRT1 expression correlated with an increase in cleaved caspase 3 expression in the brain lysates of female rats that self-administered nicotine compared to saline controls. Male rats that self-administered nicotine did not differ in SIRT1 and cleaved caspase 3 expression compared to the saline controls. (**B**) Quantification of the blots from all the animals used in the study is represented as a histogram below. Overall 2-Way ANOVA (hatched line) with Sidak’s multiple comparison test (*p < 0.05, **p < 0.01, ***p < 0.001, ****p < 0.0001) was used to determine significance. ns- not significant.

As miRNAs can regulate the expression of several gene targets, we performed bioinformatics analysis to assess the common targets of miR-199 and 214 (Supplemental Table 3). One prominent target we identified was SIRT1 which plays a significant role in cellular processes^33^. Western blot analysis on the brain lysates showed a significant decrease in SIRT1 expression in the nicotine exposed female rats (3-way interaction of Sex x Group x Sesssion F(1,24) = 7.231, p = 0.013) but not in the males (Fig. 4A,B). The decrease in SIRT1 levels in nicotine self-administering females correlated with increased expression of cleaved caspase 3, a pro-apoptotic protein (3-way interaction of Sex x Group x Sesssion F(1,24) = 17.81, p < 0.001). Cleaved caspase did not differ between the male nicotine self-administration and yoked saline controls. To further elucidate if other brain regions unrelated to drug abuse also show sex-specific difference with SIRT1 expression, we examined the cerebellum and hippocampus. We observed no significant change in SIRT1 expression in both the brain regions with nicotine intake between the two sexes (Supplementary Figs 1 and 2). These data collectively show that down regulation of SIRT1 by nicotine is specific to regions involved in drug seeking and more specifically in female rats. In summary, our current findings demonstrate the co-expressed miRNAs 199 and 214, and their common gene target SIRT1 to be associated with chronic nicotine taking in female, but not male rats.

## Discussion

Studies investigating potential sex differences in nicotine dependence have now begun to document evidence that women, despite being less prone to smoking, often show more pronounced measures of withdrawal and are more likely to relapse^6,35–38^. Using the rodent IV drug self-administration model that mimics human drug-taking behavior, we allowed male and female rats to self-administer IV nicotine for 22 days. Consistent with our recently published study^20^, we found that both male and female rats readily self-administered nicotine. In that study, we found that female rats had higher total nicotine intake than males (+1.26 fold), although in that study it did not meet the threshold for statistical significance. In the current report, we observed the same pattern with total intake in the females significantly higher than the males (+1.35 fold). Higher nicotine intake in females is in line with previously published works^5,17,39–42^. One possible reason for the higher intake in females could be the stage of estrous, which has been associated with the observed sex differences with drugs of abuse^43,44^. Previous studies have reported that intact female rats show greater behavioral response to psychostimulants such as cocaine in estrus compared to other stages of the estrous cycle^45^ or to males^46^. This observation further draws support from another study which showed females reaching higher breaking points during estrus than during other stages of the estrous cycle on a progressive ratio of cocaine self-administration^47^.

While there have been reports of some behavioral sex differences associated with chronic nicotine intake, the molecular mechanisms contributing to these observed sex differences remain poorly explained. The goal of the current study was to identify sex-specific miRNA signatures associated with chronic nicotine intake. miRNAs represent a class of noncoding RNAs that critically regulate gene expression by binding to the 3′ UTR of target transcripts, thus blocking their translation. Several studies have documented the role of these miRNAs in a plethora of diseases including maladaptive behavior associated with addiction to drugs of abuse^22–24^. Our current study did identify differentially expressed miRNAs between male and female rats post nicotine self-administration. While the relative expression levels of the significantly regulated miRNAs in males were subtle, females showed more robust changes in expression. The increase in miRNA expression may be a result of the elevated nicotine intake in the females, including the role of estrous as noted above, compared to the males.

Among the differentially regulated miRNAs in nicotine-taking females, we found miR-199 and 214, which are co-expressed on chromosome 1, to be up regulated. Co-expression studies showing the implications of miR-199a and 214 dysregulation have been very limited. Studies have implicated these miRNAs in several types of cancers^48–50^, and their roles have been documented in neural development^51,52^. Using a self-administration model of drug-intake, this study revealed that miR-199a and 214 may serve as novel markers associated with sex differences in nicotine intake. Post-nicotine exposure, females showed a significant increase in both miR-199a and 214 expression compared to the yoked saline controls. In the males, miR-199a was slightly increased in the nicotine group while miR-214 was decreased, albeit both of these miRNAs did not differ significantly compared to the saline controls. These findings were validated by RT-PCR.

miRNAs play a critical role in regulating gene expression and can target several gene targets. We performed a bioinformatics search that identified SIRT1 as one of the common targets for both miR-199a and 214. Western blot confirmed a significant reduction in SIRT1 in female rats that self-administered nicotine vs females that were in the saline group, an effect that was not found in the male groups. SIRT1 is a NAD- dependent deacetylase belonging to the class III histone/protein deacetylases and a member of the silent information regulator family that has been shown to play a significant role in several cellular processes in a number of diseases^33^. miRNAs have been shown to regulate the expression of SIRT1 in different disease models. This includes miR-34a in fatty liver disease^53^, miR-195a in lipotoxic cardiomyopathy and diabetic retinopathy^54^, and miR-199a in cardiac myocyte hypoxia^55^. Specific to drug dependence, previous work has notably shown that SIRT1 is upregulated in the NAc in male mice and rats administered (IP) cocaine, morphine, or heroin^34^. Although a follow-up study by Ferguson and collegues^31,32^ revealed that although the total number of SIRT1 binding sites is increased following 7 days of IP cocaine injections, drug administration results in depletion of SIRT1 from genic regions, specifically gene promoters. Divergent results from those studies and the work presented here, are likely a result of the type of drug utilized, the region of the brain examined, the sex and type of animal model employed, and the drug administration paradigm used.

In terms of neurodegenerative disorders, upregulation of miR-142 has been shown to decrease SIRT1 levels in a macaque model of neuroAIDS^56,57^. A recent study demonstrated that upregulation of miR-199a-5p caused a significant decrease in SIRT1 levels that subsequently led to neuronal loss and apoptosis in a rat model of epilepsy^58^. The role of SIRT1 in exerting neuroprotection both *in vivo* and *in vitro* has been well documented^59^. In an Alzheimer’s disease model, overexpression of SIRT1 caused reduction in beta amyloid production and toxicity^60^ as well as prevented neurotoxic tangles by deacetylating the microtubule binding protein tau^61^. In Huntington’s disease, SIRT1 overexpression ameliorates the neurotoxic effect of the mutant protein HTT while overexpression reverses this effect^59^. In a rodent model of Parkinson’s disease, overexpression of SIRT1 caused a reduction in alpha synuclein aggregates and increased longevity while knockout mice displayed opposite effects^62^.

The current study shows that the upregulation of miR-199 and 214 post-nicotine exposure in female rats resulted in a significant decrease in SIRT1 levels, which led to an increase in the expression of the pro-apoptotic protein cleaved caspase 3 in the PFC, but not in the cerebellum and hippocampus. Decreased neuroprotection specifically in the PFC via SIRT1 in females following long-term nicotine administration may play a role in female increased vulnerability to nicotine relapse^6,38,63^ and heighted sensitiveity to nicotine withdrawal^4,17–19^. Future work including a yoked-nicotine control will allow careful examination of how nicotine reinforcment, compared to passive nicotine intake, may alter expression of miRNAs. Additionally, a study holding nicotine intake constant across sex will allow determination if miRNA expression differences may be a result of differential exposure to nicotine in males and females. Future directions of this work will also include examining the effect of molecular manipulation of SIRT1 in the PFC of female and male rats on nicotine self-administration. Further, studies aimed at dissecting mechanisms associated with the increase in such apoptosis signaling molecules will lend significant insights into understanding the sex-based differences associated with chronic nicotine intake.

## Materials and Methods

### Animals

Sprague-Dawley rats (14 males; 15 females) were purchased from Harlan Laboratories, Inc. (Indianapolis, IN, USA) at approximately 9 weeks of age (males 275–300 g; females 175–200 g). Rats were individually housed in clear polycarbonate cages (48.3 × 26.7 × 20.3 cm) lined with TEK-Fresh cellulose bedding. Rats were allowed *ad libitum* access to water in home cages throughout the experiment. Following 3 days of acclimation to the colony room, food was restricted to maintain 90% of a rat’s free-feeding weight. The colony room was temperature and humidity controlled and maintained on a 12 h:12 h light/dark cycle. All experimental procedures were performed during the light cycle. Animal procedures and methods employed were approved by both the University of Nebraska-Lincoln and University of Nebraska Medical Center Institutional Animal Care and Use Committee in accordance with the relevant guidelines and regulations.

### Apparatus

Experimental procedures were conducted in 10 conditioning chambers (ENV-008CT; Med Associates, Georgia, VT), measuring 30.5 × 24.1 × 21.0 cm, enclosed in sound-attenuating cubicles. Each chamber had a variable-speed syringe pump (PMH-100VS; Med-Associates) located outside the cubicle. Tygon^®^ tubing was threaded from the pump syringe through a leash into the chamber to be attached to the catheter port. A recessed receptacle (5.2 × 5.2 × 3.8 cm) was centered on one sidewall of each chamber. A dipper arm could be raised to provide access to 0.1 ml of 26% (w/v) sucrose in this recessed receptacle. Two retractable levers were located on each side of the receptacle. A white cue-light (2.54 cm diameter; 28 V, 100-mA) was mounted 7 cm above each lever and a house-light (two white 28 V, 100-mA lamps) was located in the cubicle, 10 cm above the chamber ceiling. An infrared emitter/detector unit was located 4 cm above the stainless steel rod floor and 14.5 cm from the side wall containing the receptacle. The number of times this beam was broken provided a measure of chamber activity.

### Drugs

(-)-Nicotine hydrogen tartrate, obtained from Sigma-Aldrich (St. Louis, MO, USA), was dissolved in sterile saline and pH adjusted to 7.0 ± 0.2. Nicotine was infused intravenously at 35.74 μl over 1 sec at 0.0073 mg/infusion (i.e., 0.03 mg/kg/infusion given an average weight of 243 g per rat). Nicotine dose is reported as the free base.

### Preliminary Lever Training

Following acclimation and food restriction, rats were trained to lever press in our lab’s standard procedure^20,64,65^. Preliminary lever training sessions were initiated by illumination of the house light and insertion of one of the two levers (randomly selected). A lever press or a lapse of 15 sec resulted in 4-sec access to sucrose, lever retraction, and a timeout (average duration = 60 sec; range = 30–89 sec). After the timeout, one of the two levers was again inserted randomly with the condition that the same lever would not be presented more than twice in a row. These procedures were repeated until all rats received 60 sucrose deliveries. Daily sessions ranged from 65 to 80 min depending on individual performance. Rats received daily sessions until a lever press was made on at least 80% of the lever insertions; all rats learned within 3–5 sessions.

### Indwelling Jugular Catheter Surgery and Recovery

Rats were then implanted with indwelling jugular catheters following our lab’s standard procedures^20,64,66^. Briefly, rats were anesthetized with a 2:1 ketamine HCl (100 mg/kg) + xylazine HCl (20 mg/kg) cocktail (IM) and prepped for surgery. Two small incisions were made below the scapula and the back mount catheter was fit under the skin with the back plate lying flat and the cannula port exiting the more rostral incision. Another small incision was then made above the right jugular vein and the catheter tubing pulled subcutaneously from the back, over the shoulder, to the neck. The vein was then isolated and the catheter tubing fed into the vein and fastened securely. All incisions were then sutured and the rats administered buprenorphine (0.1 mg/kg) for pain maintenance (also administered 24 h post-surgery) and atipamezole (0.5 mg/kg) to terminate anesthesia. Rats then recovered for 7 days, during which they remained in their home cages and their catheters were flushed daily with a cocktail of 0.2 ml cefazolin (50 Units/ml) and heparin (30 Units/ml). Patency was checked on the last day of recovery by IV infusion of 0.05 ml xylazine (20 Units/ml). Rats that displayed ataxia (e.g., impaired locomotor function, sedation) within 20 sec were considered patent. Rats that were not patent (1 male; 4 females) were excluded from the study.

### Post-Surgery Lever Training

After recovery, rats received training sessions with sucrose available on a variable ratio (VR) 3 schedule; on average every third response was reinforced (range 1–5 presses). This training consisted of 3 daily sessions; during which 1 of the 2 levers was extended into the chamber and then retracted following requisite responding. Requisite responding resulted in 4-sec access to sucrose. After sucrose access, the opposite lever was inserted and functioned as the active lever. This process was repeated until the end of the 1-h session. These procedures produce robust lever responding with each lever having an equivalent learning history^65^.

### Self-Administration

Prior to the self-administration phase of the experiment, female and male rats were separated into two groups creating a total of 4 groups (sample size from post-patency test): nicotine self-administration groups (nic-males, n = 7; nic-females, n = 6) and saline-yoked groups (sal-males, n = 6; sal-females, n = 5). During self-administration sessions (1 h) both levers were inserted to begin the session. Rats in the nicotine self-administration groups received nicotine infusions following responding on 1 of the 2 levers; responding on the other lever was recorded but held no programed consequence (i.e., only pressing on the active lever was reinforced). Assignment of active and inactive lever was counterbalanced for all groups. The schedule of reinforcement in the self-administration phase was again a VR 3. Following the nicotine infusion, a 20-sec timeout was signaled by the house light turning on and extraction of the levers. Levers were again inserted and the house light turned off following the timeout. Rats in the saline-yoked groups were presented with two inactive levers (i.e., responding was recorded but pressing held no programed consequence). Saline-yoked rats received saline infusions and signaled timeouts matched to a nicotine self-administering partner’s response pattern. This procedure produced controls that had equated handling, chamber exposure, time-outs, etc. yet they were void of nicotine exposure. Self-administration sessions were conducted daily for 22 days. Brains were extracted 24 h after the last self-administration session. Individual brain regions were immediately dissected, rapidly frozen, and stored at −80 °C until further use.

### Dependent Measures

Active lever pressing, inactive lever pressing, and activity were the dependent measures utilized during the self-administration phase. Nicotine intake controlling for body weight was calculated by the equation: nicotine intake = [(group average weight (g) used to calculate dose/rat’s weight (g) on each self-administration day) * 0.03 mg * infusions earned]. Using this calculation, we were able to keep the dose of nicotine per infusion constant and still properly assess possible differences in total nicotine intake^20^. Total nicotine intake was calculated by summing nicotine intake on all self-administration sessions.

### Total RNA extraction, Quality control, library preparation and small RNA-Seq

Total RNA from the PFC was isolated from individual rats from both sexes and treatment groups using the Direct-Zol RNA kit (Zymo Research, USA) based on the manufacturer’s protocol. Quantitation of extracted total RNA used Nanodrop 1000 (Thermo Fisher Scientific Inc., USA) and RNA integrity was determined by Agilent 2100 Bioanalyzer (Agilent Technology, USA). RNA samples were then sent on dry ice to LC Sciences (Houston, TX, USA) for miRNA sequencing. Briefly, total RNAs with a RIN value >7 were selected for library construction. A small RNA library was generated from the sample using the Illumina Truseq™ Small RNA Preparation kit according to Illumina’s TruSeq™ Small RNA Sample Preparation Guide. The purified cDNA library was used for cluster generation on Illumina’s Cluster Station and then sequenced on the Illumina HiSeq platform. Raw sequencing reads (50 nt) were obtained using Illumina’s Sequencing Control Studio software version 2.8 (SCS v2.8) following real-time sequencing image analysis and base-calling by Illumina’s Real-Time Analysis version 1.8.70 (RTA v1.8.70).

After trimming the adaptor sequence from the raw reads, mappable reads with the length ranging from 15 to 32 bases were mapped to miRBase v21 and the rat genome. Reads mapped to rat miRNAs in miRbase were annotated and the number of mapped reads counted for each identified rat miRNA. The raw counts of reads were normalized by the library size parameter of the corresponding sample. The library size parameter is a median value of the ratio between the counts of a specific sample and a pseudo-reference sample. A count number in the pseudo-reference sample is the count geometric mean across all samples and was calculated using the formula

$${S}_{j}=\mathop{median}\limits_{{\rm{i}}}(\frac{{c}_{ij}}{{({\prod }_{k=1}^{m}{c}_{ik})}^{1/m}})$$

where *S*_*j*_ is the library size parameter; *c*_*ij*_ is the count number of sequence *i* of sample *j; m* is the total number of samples involved.

### Quantiative Real Time PCR

TaqMan miRNA and Gene Expression Assays (Life Technologies, Carlsbad, CA, USA) were used for cDNA synthesis and real time PCR according to manufacturer’s instructions. Small nuclear RNA U6 (U6) was used as a control for miRNA studies and glyceraldehyde 3-phosphate dehydrogenase (GAPDH) was used as a control for mRNA studies. Delta-delta Ct method^67^ was used to calculate fold change (2^−((CtmiRNA − CtU6)exp − (CtmiRNA − CtU6)control)^) or (2^−((CtmRNA − CtGAPDH)exp − (CtmRNA − CtGAPDH)control)^). Statistical significance was determined using ΔCt values (^CtmiRNA − CtU6) or (CtmRNA − CtGAPDH^).

### Bioinformatics Analysis

The detailed sequence information of miR-199 and miR-214 was extracted from miRBase (Release 21)^68,69^. Targets for miR-199 and miR-214-3p were identified using experimentally validated miRNA-target interaction database - miRTarBase (Release 6.0)^70,71^. TargetScan^72,73^ was used to predicted additional targets for miR-199-5p and miR-214-3p.

### Western blot

Western blot using SDS-PAGE electrophoresis was performed using NuPAGE gel system (Invitrogen) as described in our previous studies^74,75^. Briefly, 30 µg (SIRT1) and 10 µg (cleaved caspase 3) of PFC brain lysates from all the animals were separated on a 4–12% and 10% Bis-Tris gels respectively under reducing conditions followed by transfer and immunodetection. Nonspecific antibody binding was blocked using Superblock (Thermofisher Scientific, Rockford, IL, USA) for 1 h at room temperature. Immunoblotting was carried out with antibodies against SIRT1 (1∶1000, Santa Cruz Biotechnology, Santa Cruz, CA) and cleaved caspase-3 (1: 1000, Cell Signaling,Technology, Danvers, MA) followed by secondary antibody (1∶3,000 HRP conjugated anti rabbit IgG for SIRT1 and 1∶3000 HRP conjugated anti rabbit for cleaved caspase-3 respectively (Thermofisher Scientific, Rockford, IL, USA). Blots were developed with 1∶1 solution of Super Signal West Pico Chemiluminescent Substrate and Luminol/Enhancer (Thermo Fisher Scientific, Rockford, IL, USA).

### Statistical Analyses

Active lever presses, inactive lever presses, and chamber activity were analyzed by separate 3-way mixed measures ANOVAs (Type III Sum of Squares) with Sex (male vs female) and Group (nicotine vs saline) as between-subjects factors and Session (22) as a within-subjects factor. An independent samples t test was used to analyze differences in total nicotine intake between females and males in the nicotine self-administration groups. Least Significant Difference (LSD) tests were utilized for post-hoc analysis. Statistical significance was declared at p < 0.05. For miRNA analysis, after normalization, a student t-test was done to identify miRNAs showing significant differences between groups (saline versus nicotine for each of the sex). miRNAs were filtered using False Discovery Rates (FDR) ≤ 5% and p < 0.05 were considered significant. For the western blots, 2-way ANOVA followed by Sidak’s multiple comparision test with p < 0.05 was considered significant. All statistical tests were performed with GraphPad Prism (La Jolla, CA, USA); data represented as Mean ± SEM on the graphs.

## Electronic supplementary material

Supplemental Datasets

## Data Availability

Most of the data generated or analyzed during this study are included in this published article (and its Supplementary Information file). All datasets generated during and/or analyzed in the current study are available from the corresponding author on reasonable request. All Med-PC programs used to control the behavioral equipment are available upon request to RAB at rbevins1@unl.edu.
