# Supplementary material for: MicroRNA cluster miR199a/214 are differentially expressed in female and male rats following nicotine self-administration
Source: Sci Rep. 2018 Nov 30;8:17464. doi: 10.1038/s41598-018-35747-z (PMC6269448; doi:10.1038/s41598-018-35747-z)
Supplement: Supplementary file 1 — Supplemental Datasets [file 41598_2018_35747_MOESM1_ESM.pdf]

MicroRNA cluster miR199a/214 are differentially expressed in female and male rats following nicotine self-administration.

Steven T. Pittenger[1 #], Victoria L. Schaal[3], Dalia Moore[2], Rahul S. Guda[3], Sneh Koul[3], Sowmya V. Yelamanchili[2], Rick A. Bevins[1], and Gurudutt Pendyala[3*]

Supplemental Information.

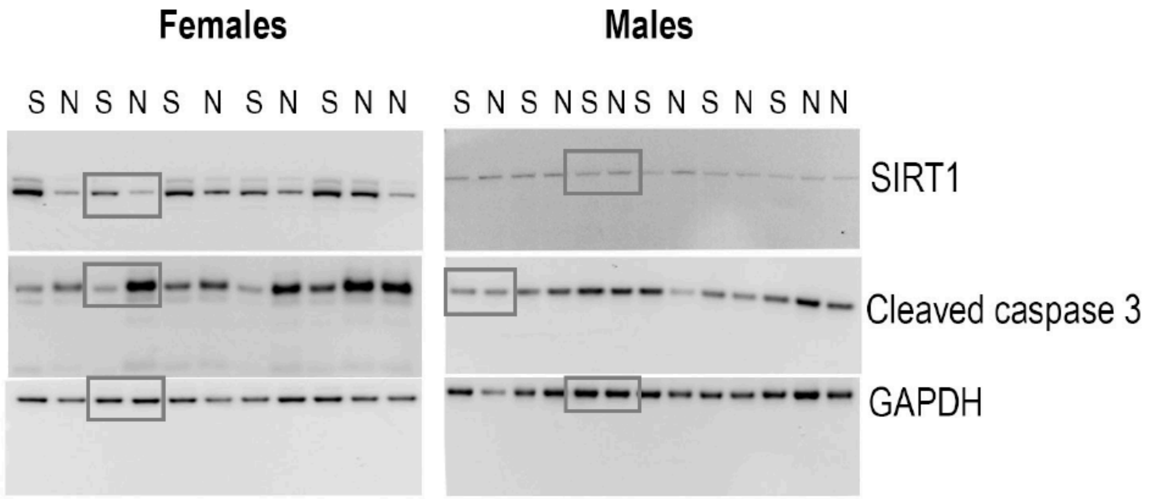

Supplementary Fig 1: Individual western blots on the PFC lysates from all the animals used in the study. Boxed blots are represented in the manuscript. S-Saline, N-Nicotine.

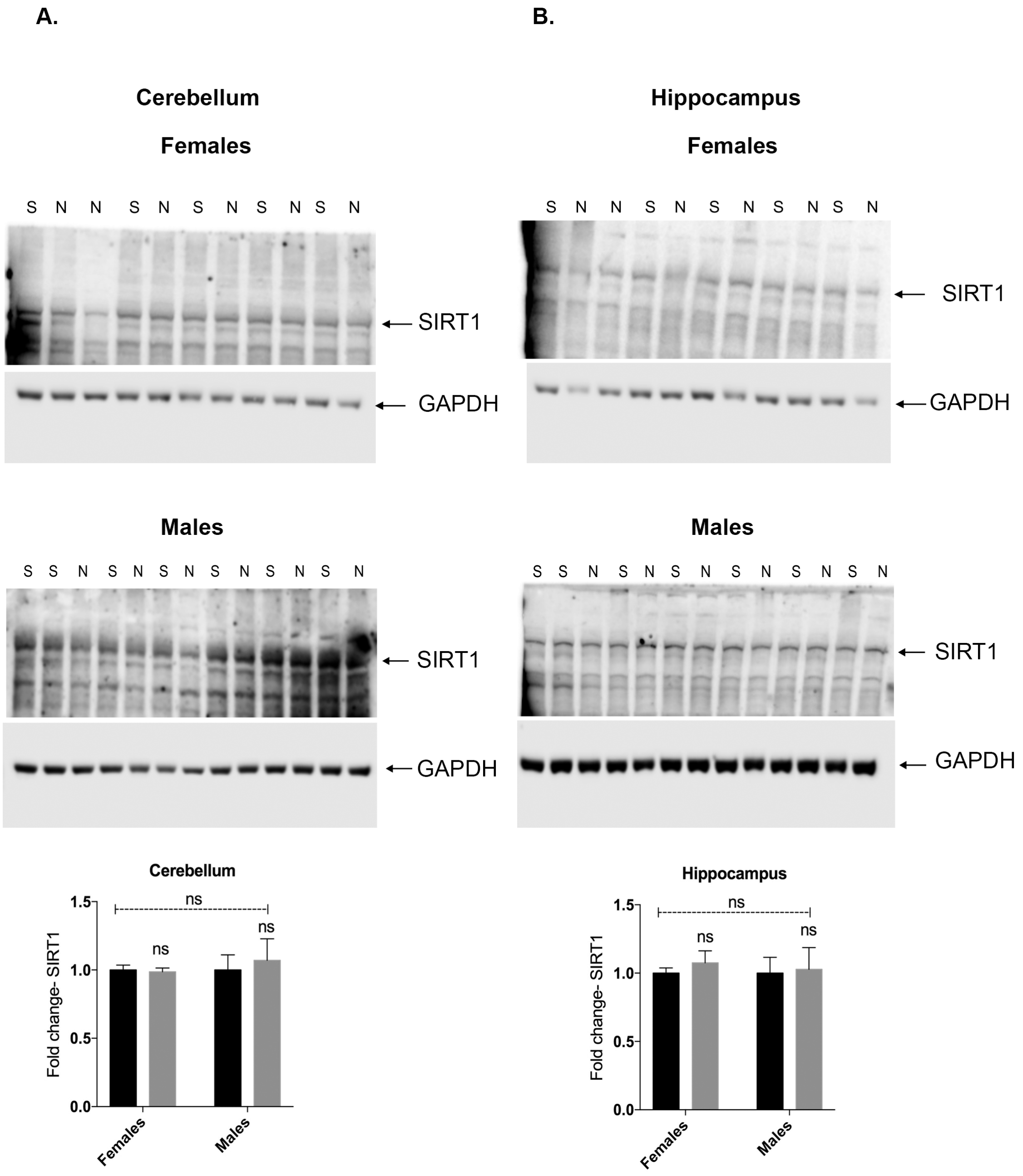

Supplementary Fig 2: Western blots for SIRT1 expression in cerebellum (A) and Hippocampus (B) from all the animals including quantification of the blots used in the study is represented as a histogram below. Overall 2-Way ANOVA (hatched line) with Sidak's multiple comparison revealed no overall effect in the two brain regions as well as between the sexes after nicotine exposure.

Sample	# raw reads	# of reads containing 3ADT	# reads <15 nt after removing 3ADT	# reads after removing 3ADT	# mappable reads	% mapped reads
MS1	17,755,895	17,619,575	2,378,531	15,241,044	15,161,162	85.4
MS2	33,601,446	33,070,469	2,195,576	30,874,893	30,704,125	91.4
MS3	34,875,267	34,528,273	1,602,714	32,925,559	32,779,376	94.0
MS4	9,321,584	9,149,808	290,543	8,859,265	8,804,709	94.5
MS5	26,068,132	25,364,771	1,409,985	23,954,786	23,787,918	91.3
MS6	20,071,802	19,967,339	1,100,654	18,866,685	18,748,153	93.4
MS7	14,142,315	14,068,614	851,745	13,216,869	13,131,992	92.9
MN1	7,265,100	7,206,827	409,530	6,797,297	6,765,368	93.1
MN2	18,137,020	18,054,322	1,237,190	16,817,132	16,731,239	92.2
MN3	23,388,051	23,208,417	1,161,318	22,047,099	21,917,872	93.7
MN4	15,524,292	15,373,315	522,380	14,850,935	14,765,979	95.1
MN5	17,752,867	17,565,527	691,313	16,874,214	16,774,555	94.5
MN6	7,809,742	7,745,704	298,984	7,446,720	7,389,571	94.6
FS1	9,029,216	8,966,320	385,108	8,581,212	8,506,928	94.2
FS2	8,801,501	8,579,305	262,688	8,316,617	8,235,811	93.6
FS3	10,423,363	10,183,871	538,104	9,645,767	9,541,991	91.5
FS4	7,583,966	7,387,585	198,430	7,189,155	7,123,257	93.9
FS5	6,423,507	6,374,972	589,866	5,785,106	5,741,550	89.4
FN1	19,415,644	19,238,159	843,722	18,394,437	18,248,846	94.0
FN2	20,650,546	20,427,333	717,009	19,710,324	19,549,953	94.7
FN3	5,637,587	5,373,271	95,803	5,277,468	5,226,055	92.7
FN4	20,862,458	19,399,753	544,894	18,854,859	18,677,070	89.5
FN5	6,496,883	5,953,563	150,071	5,803,492	5,752,942	88.5
FN6	19,697,802	19,609,631	3,064,449	16,545,182	16,457,596	83.6

Supplemental Table 1. Number of raw and mapped reads in individual animals post nicotine self-administration

Index	miR_seq	miR_name	raw_MS1	raw_MS2	raw_MS3	raw_MS4	raw_MS5	raw_MS6	raw_MS7	raw_MN1	raw_MN2	raw_MN3	raw_MN4	raw_MN5	raw_MN6	raw_FS1	raw_FS2	raw_FS3	raw_FS4	raw_FS5	raw_FN1	raw_FN2	raw_FN3	raw_FN4	raw_FN5	raw_FN6
1	GAATTGCAGTACTCCAACATTC	rno-mir-3068-p3	4,538	8,506	12,339	3,490	8,587	7,264	4,126	2,383	6,598	5,892	4,177	4,204	1,745	2,424	1,892	2,898	1,621	2,171	6,925	4,995	962	2,717	912	3,941
2	TCTTTGGTTATCTAGCTGTATGA	rno-miR-9a-5p	83,342	196,435	271,454	49,022	102,290	102,436	60,174	66,647	119,685	95,245	72,325	68,814	22,635	26,351	17,653	19,544	13,800	22,532	62,291	72,520	12,435	32,199	10,917	38,340
3	ATAAAGCTAGATAACCGAAAGT	rno-miR-9a-3p	21,768	45,137	54,837	9,895	28,173	29,667	14,829	12,727	27,994	21,167	16,822	16,115	9,514	8,301	6,791	7,189	4,054	6,697	12,860	15,359	3,890	11,736	3,729	12,359
4	CACCCGTAGAACCGACCTTGCG	rno-miR-99b-5p	84,897	238,026	313,212	58,911	109,591	111,919	62,278	80,689	142,433	59,178	46,254	59,434	24,270	25,500	18,726	19,036	13,671	23,687	51,895	69,656	11,851	30,862	11,022	40,684
5	TTTGGCACTAGCACATTTTTGCT	rno-miR-96-5p	1,480	1,503	1,121	398	283	708	177	156	6,570	13,594	24,333	367	67	452	229	183	111	1,030	53,228	152	39	140	5	34
6	TATTGCACTCGTCCCGGCCTCC	rno-miR-92b-3p	11,900	16,859	20,990	5,147	8,762	12,072	7,843	6,065	10,183	11,314	6,758	7,134	2,840	2,505	2,454	1,802	1,189	2,335	4,541	4,875	1,062	4,019	1,445	7,865
7	TGGAAGACTAGTGATTTTGTTGTT	rno-miR-7a-5p_R+1	9,187	17,680	70,142	7,277	13,220	12,794	5,914	9,843	13,223	11,123	6,029	5,801	2,141	4,552	2,038	1,984	1,308	5,011	8,359	6,557	1,539	3,936	1,141	3,829
8	AGCTCGACTCATGGTTTGAACCA	rno-miR-434-5p	16,088	35,957	42,298	9,746	20,769	19,720	13,241	10,632	21,562	15,911	13,047	13,352	5,860	5,506	4,378	4,044	3,542	4,736	7,624	15,058	3,278	8,032	2,399	9,663
9	TTTGAACCATCACTCGACTCCT	rno-miR-434-3p	64,737	101,591	121,802	26,173	60,352	63,723	40,222	33,648	69,057	45,770	33,628	35,578	16,530	14,824	12,272	13,056	8,499	13,462	23,700	36,178	7,490	20,701	7,857	52,773
10	TAATACTGTCTGGTAATGCCGT	rno-miR-429	185	129	107	22	17	96	22	13	699	4,439	4,010	34	7	42	25	19	9	349	7,640	7	2	12	0	4
11	AGGTTACCCGAGCAACTTTGCAT	rno-miR-409a-5p	18,149	41,315	58,096	12,589	25,016	23,186	15,435	12,522	26,064	17,461	13,348	16,295	5,684	5,327	4,437	3,650	3,739	5,117	7,655	16,740	3,123	8,347	2,494	9,102
12	TGTAAACAATTCCTAGGCAATGT	rno-miR-384-5p	7,245	14,260	18,465	3,747	9,660	7,992	4,275	3,893	9,586	4,943	3,909	3,687	2,878	2,567	2,082	2,420	1,397	2,742	3,633	6,262	1,511	3,385	1,085	3,642
13	ATTCCTAGAAATTGTTCACAAT	rno-miR-384-3p	10,132	20,779	24,919	4,631	13,150	13,117	6,627	5,335	12,905	8,439	7,623	6,851	3,258	3,085	2,552	2,674	1,891	3,147	4,913	6,310	1,701	4,189	1,565	6,641
14	TCTCACACAGAAATCGCACCCGTC	rno-miR-342-3p_R+1	6,151	17,076	18,609	3,963	7,668	8,139	5,342	4,546	9,009	7,439	5,417	5,521	2,448	2,235	1,800	1,705	1,327	1,898	4,136	5,366	1,110	3,180	1,360	5,011
15	CTGGCCCTCTCTGCCCTTCCGT	rno-miR-328a-3p	4,690	8,118	11,131	1,684	4,498	4,733	2,996	2,433	5,183	5,832	3,408	3,958	1,545	1,499	1,308	1,275	879	1,117	2,682	3,341	585	2,096	1,127	7,607
16	TTTATTGAGCACCTCCTATCA	rno-miR-325-3p_R-1	7,857	13,442	20,787	3,624	8,030	8,298	3,846	5,173	9,609	3,994	3,112	3,198	1,619	1,487	1,138	1,327	819	1,615	1,850	2,973	711	1,729	672	3,119
17	TGTAAACATCCTTGACTGGAAGCT	rno-miR-30e-5p_R+2	87,679	161,977	177,198	32,836	87,184	83,106	52,246	37,557	91,540	74,970	67,601	58,725	27,830	24,206	21,996	18,781	16,850	22,225	52,694	59,751	11,049	39,334	12,299	49,276
18	TGTAAACATCCCCGACTGGAAGCT	rno-miR-30d-5p_R+2	76,903	136,724	108,911	29,424	63,400	53,280	52,675	25,960	84,309	49,473	40,137	35,933	21,994	16,342	16,957	15,239	9,691	18,288	34,702	39,661	9,243	35,413	10,055	31,705
19	TGTAAACATCCTACACTCTCAGCT	rno-miR-30c-5p_R+1	37,407	81,497	76,568	15,611	44,552	41,772	25,187	20,314	42,028	37,447	31,267	27,344	15,373	12,741	11,150	9,841	7,339	9,977	23,255	30,437	6,392	21,023	7,326	33,236
20	TGTAAACATCCTACACTCAGCT	rno-miR-30b-5p	27,231	48,596	39,603	9,668	29,701	21,195	16,671	9,364	32,504	17,464	14,290	12,151	7,147	5,775	5,334	5,319	2,832	5,844	9,580	10,759	3,092	9,989	2,973	11,929
21	TGTAAACATCCTCGACTGGAAGCT	rno-miR-30a-5p_R+2	48,033	108,182	105,641	24,664	54,694	56,363	38,678	23,620	53,158	58,629	49,116	45,116	19,804	16,607	15,522	12,573	11,481	14,152	35,215	44,686	8,283	28,400	9,510	34,093
22	TAGCACCATCTGAAATCGGTT	rno-miR-29a-3p_R-1	199,349	292,310	436,642	68,835	193,946	194,741	95,212	91,513	216,884	124,923	85,999	84,508	50,378	46,853	37,667	47,071	23,472	42,922	67,306	96,890	23,837	57,963	19,548	96,821
23	TTCACAGTGGCTAAGTTCTGC	rno-miR-27b-3p	84,504	193,180	204,012	54,360	109,953	105,650	70,995	53,265	102,557	87,308	72,699	72,621	28,204	26,497	22,142	19,769	16,374	25,076	47,393	75,778	12,747	36,113	11,336	51,422
24	TTCACAGTGGCTAAGTTCCGC	rno-miR-27a-3p	5,615	15,467	13,201	2,864	8,436	8,386	4,948	2,589	6,769	8,043	6,224	6,647	2,909	2,305	2,249	1,925	1,465	1,911	3,737	5,149	1,201	3,196	1,255	6,374
25	TTCAAGTAATTCAGGATAGGTT	rno-miR-26b-5p_R+1	6,347	14,718	20,086	3,546	9,359	9,536	4,947	3,955	8,641	9,044	7,057	6,026	2,660	2,632	2,055	2,003	1,332	2,250	5,635	5,921	1,204	3,186	1,154	4,468
26	TTCAAGTAATCCAGGATAGGCT	rno-miR-26a-5p	298,287	779,203	584,505	181,733	395,457	401,878	284,627	165,252	353,367	397,034	344,266	306,716	126,912	124,549	98,253	75,706	65,594	100,823	273,919	277,487	49,659	164,982	52,767	200,090
27	TGGCTCAGTTCAGCAGGAAC	rno-miR-24-3p_R-2	10,801	27,475	25,189	6,820	15,255	13,745	9,226	4,341	14,147	13,043	10,974	12,161	6,136	5,231	4,872	4,519	3,314	5,031	8,883	13,236	2,216	6,296	2,200	11,946
28	AGCTACATCTGGCTACTGGGTCTCT	rno-miR-222-3p_R+4	10,405	22,465	18,580	4,562	9,948	10,680	8,144	5,818	10,601	14,132	9,360	9,753	4,018	3,731	2,808	2,830	1,918	2,940	5,730	10,586	1,636	5,866	1,843	9,160
29	ACCTGGCATACAATGTAGATTTCT	rno-miR-221-5p_R+1	5,600	14,471	13,938	4,003	7,406	7,464	4,932	3,181	6,568	5,940	4,065	5,281	2,006	1,652	1,669	1,337	1,420	1,469	2,959	6,200	1,153	3,446	999	2,961
30	AGCTACATTGTCTGCTGGGTTT	rno-miR-221-3p_R-1	14,168	28,224	35,295	6,376	13,864	15,275	10,387	9,245	17,810	16,921	11,074	11,671	4,165	4,416	2,798	3,788	2,153	3,743	6,193	11,738	1,915	5,957	2,067	10,861
31	AAGCTGCCAGTTGAAGAACTGT	rno-miR-22-3p	43,231	74,955	68,717	16,144	48,021	46,771	28,527	16,919	42,338	36,460	31,991	26,875	16,500	12,544	11,614	10,568	6,846	12,584	17,383	25,870	6,337	18,049	5,977	43,664
32	ACCTTGGCTCTAGACTGCTTACT	rno-miR-212-5p_R-1	25,434	51,081	63,638	17,176	27,554	26,947	16,068	16,565	32,599	18,534	15,262	15,979	6,226	5,563	4,768	4,133	3,389	5,914	8,321	16,704	2,837	7,836	2,601	12,809
33	TTCCCTTTGTCATCCTATGCCT	rno-miR-204-5p	3,294	10,549	10,688	1,867	4,440	4,836	3,094	1,788	4,061	8,059	6,630	3,890	1,377	1,398	1,227	861	950	1,277	7,694	2,748	651	1,994	748	2,415
34	TAATACTGCCTGGTAATGATGA	rno-miR-200b-3p_R-1	992	590	441	142	90	390	82	41	2,850	15,973	13,723	147	39	218	96	104	55	1,228	30,700	66	9	56	0	19
35	TAACACTGTCTGGTAACGATGT	rno-miR-200a-3p	1,363	1,165	713	195	176	525	108	74	5,070	25,866	25,645	298	70	386	159	140	73	2,057	55,637	167	11	121	7	30
36	CAACGGAATCCCAAAAGCAGCTG	rno-miR-191a-5p	61,023	166,021	156,189	39,469	86,214	88,610	57,922	34,288	78,251	85,531	73,231	72,236	36,654	31,052	27,136	20,830	18,313	25,229	58,833	74,216	15,184	46,435	14,649	53,989
37	CAAAGAATTCTCCTTTTGGGCT	rno-miR-186-5p	10,015	25,670	25,269	5,622	11,988	11,998	8,414	5,529	10,541	12,065	12,450	10,092	4,613	4,866	3,648	2,791	2,959	3,849	9,355	11,392	2,753	7,536	2,510	8,957
38	TATGGCACTGGTAGAATTCACT	rno-miR-183-5p	1,020	850	1,056	204	188	370	94	54	3,231	11,182	12,567	224	29	233	114	99	60	664	31,974	85	5	85	0	14
39	TTTGGCAATGGTAGAACTCACACC	rno-miR-182_R-1	2,176	1,567	1,636	480	308	820	191	179	7,274	19,308	33,869	368	62	498	157	177	88	1,469	58,619	169	18	139	10	27
40	AACATTCAACGCTGTCGGTGAGT	rno-miR-181a-5p	25,364	50,234	70,145	11,284	26,183	24,699	18,191	17,541	33,441	22,217	18,407	15,625	6,705	7,076	4,665	4,984	3,406	6,542	13,569	15,679	2,862	7,820	2,551	12,459
41	TAGCAGCACGTAAATATTGGCG	rno-miR-16-5p	7,270	16,165	15,744	3,475	9,373	8,616	5,422	3,640	9,445	7,116	6,111	5,565	3,018	2,663	2,191	2,205	1,400	2,309	6,296	5,188	1,306	3,684	1,198	4,285
42	TTGCATAGTCACAAAAGTGATC	rno-miR-153-3p	6,575	13,002	17,019	3,022	8,696	8,759	4,258	2,999	7,676	6,042	5,088	4,464	2,169	2,062	1,743	1,772	1,339	1,970	2,964	3,762	1,333	3,052	1,006	4,102
43	TCGAGGAGCTCACAGTCTAGT	rno-miR-151-5p	42,995	72,400	113,826	23,154	47,205	45,857	24,307	27,395	59,496	25,335	19,157	19,303	8,625	7,931	6,389	6,548	3,869	9,335	11,779	18,959	3,213	7,523	2,283	11,055
44	TGAGAACTGAATTCCATAGGCTGT	rno-miR-146b-5p	20,960	54,116	44,587	17,093	30,820	31,128	19,536	11,798	26,751	25,871	23,652	23,523	9,446	7,023	6,896	5,841	5,760	6,605	13,358	25,043	4,386	13,392	4,160	13,491
45	TGAGATGAAGCACTGTAGCT	rno-miR-143-3p_R-2	52,546	116,603	107,156	28,874	70,827	62,306	45,783	21,835	57,568	85,810	64,568	52,628	21,982	18,335	16,541	13,555	11,248	14,639	36,676	41,873	8,505	23,317	9,231	36,157
46	ATCATCGTCTCAAATGAGTCT	rno-miR-136-3p_L-1	27,486	52,110	39,589	11,015	29,154	31,335	24,164	9,529	24,974	32,756	29,200	27,662	11,879	10,515	8,836	7,612	6,264	8,767	17,071	21,153	4,136	13,559	4,148	27,262
47	ACCGTGGCTTTCGATTGTTACT	rno-miR-132-5p	9,195	14,188	12,711	3,561	9,137	9,555	5,945	3,462	9,684	7,803	6,729	6,828	3,670	2,984	2,689	2,281	1,623	2,544	3,848	5,528	1,184	4,001	1,331	8,077
48	TAACAGTCTACAGCCATGGTCG	rno-miR-132-3p	9,622	17,414	16,057	6,020	12,783	12,122	8,554	4,201	13,676	9,192	8,254	8,742	3,597	3,414	3,029	3,276	2,110	3,336	5,254	9,517	1,671	4,460	1,198	2,730
49	AAGCCCTTACCCCAAAAAGCAT	rno-miR-129-1-3p_R+3	6,859	14,500	18,348	3,913	9,027	11,050	4,800	4,730	10,940	8,172	6,348	6,601	2,425	2,035	2,596	2,479	3,026	1,683	4,672	9,504	2,678	5,635	1,715	1,945
50	TCACAGTGAACCGGTCTCTTT	rno-miR-128-3p	123,437	217,042	262,415	50,639	110,370	121,629	82,351	72,380	150,509	109,313	82,776	86,717	34,853	37,239	26,513	29,088	16,508	31,831	54,389	82,124	15,266	44,137	17,089	54,721

51	TCGGATCCGTCTGAGCTTGGCT	rno-miR-127-3p	285,985	485,931	771,304	146,212	304,004	282,868	186,760	173,431	368,379	211,326	156,429	167,462	66,452	64,890	50,539	54,195	39,507	63,365	98,021	162,735	32,432	76,528	25,885	138,656
52	CATTATTACTTTTGGTACGCG	rno-miR-126a-5p	8,753	13,461	14,151	2,830	9,111	8,811	5,175	2,841	8,978	6,731	5,545	5,092	3,514	2,785	2,280	2,711	1,419	2,792	3,604	5,618	1,388	3,242	1,050	5,356
53	TCGTACCGTGAGTAATAATGC	rno-miR-126a-3p_R-1	41,240	68,145	78,848	17,565	43,329	43,123	26,353	17,932	41,713	32,978	25,875	24,773	15,081	11,234	9,974	8,789	7,060	9,857	18,002	23,308	6,285	16,589	5,134	17,687
54	TCCCTGAGACCCTAACTTGTGA	rno-miR-125b-5p	322,669	577,257	822,134	167,858	386,579	359,925	190,782	223,095	422,168	203,321	137,532	159,462	90,050	77,093	69,032	64,436	40,996	73,367	97,304	160,625	40,505	103,101	41,591	181,308
55	TCCCTGAGACCCTTTAACCTGT	rno-miR-125a-5p_R-2	246,764	497,404	527,150	156,974	315,614	288,532	188,205	155,094	305,620	235,060	180,262	189,995	86,865	73,811	71,379	51,725	39,684	66,117	131,883	159,510	34,371	112,998	43,172	214,395
56	TAAGGCACGCGGTGAATGCC	rno-miR-124-3p	19,136	45,965	54,789	8,874	27,836	24,841	13,633	8,473	27,235	20,752	13,834	16,442	9,061	9,256	7,422	8,957	4,951	8,839	17,969	17,933	3,979	10,085	3,541	21,638
57	GTACAGTACTGTGATAACTGAC	rno-miR-101b-3p_L+1_2ss17GA22AC	6,404	11,883	15,752	2,516	6,212	6,426	4,294	3,261	6,758	7,953	5,958	5,171	1,715	2,211	1,458	1,781	1,119	1,818	5,366	4,802	901	2,445	837	4,928
58	TACAGTACTGTGATAACTGACT	rno-miR-101a-3p_R+1_1ss21AC	7,166	14,666	14,752	2,981	7,345	7,760	6,178	3,386	6,597	9,981	9,335	7,935	2,723	2,991	2,310	1,960	1,740	2,129	7,230	6,607	1,176	3,856	1,304	7,472
59	AACCCGTAGATCCGATCTTGT	rno-miR-100-5p_R-1_1ss16AT	169,144	450,378	637,689	117,406	244,929	259,200	141,125	148,603	264,804	154,728	109,693	142,301	59,009	58,921	46,163	41,966	32,875	56,808	105,580	141,758	28,067	70,057	23,037	89,525
60	TGAGGTAGTAGTTTGTGCTGTT	rno-let-7i-5p	29,909	62,721	75,453	15,712	34,244	34,576	24,137	20,695	37,627	37,669	27,031	27,354	10,530	11,108	7,563	7,667	5,223	10,041	27,932	27,445	3,952	13,527	4,340	21,981
61	TGAGGTAGTAGATTGTATAGTT	rno-let-7f-5p	72,934	135,605	185,438	42,008	89,195	84,095	47,225	51,226	91,284	65,043	47,007	48,427	18,008	18,050	14,421	14,059	8,294	17,392	39,701	48,056	6,834	22,463	6,718	26,096
62	TGAGGTAGGAGGTTGTATAGTT	rno-let-7e-5p	18,623	36,191	36,719	8,563	20,106	19,154	12,524	11,077	20,583	18,297	11,074	13,105	5,305	4,821	4,241	3,944	1,938	4,215	9,369	12,469	1,546	6,770	1,593	6,747
63	AGAGGTAGTAGGTTGCATAGTT	rno-let-7d-5p	28,700	54,377	79,807	14,303	34,571	31,649	16,546	18,818	35,541	24,855	16,227	18,193	7,255	6,881	5,558	5,984	3,286	6,502	14,501	17,089	2,624	8,549	2,319	11,435
64	TGAGGTAGTAGGTTGTATGGTT	rno-let-7c-5p	65,168	123,852	143,703	30,507	72,569	71,037	46,894	37,929	74,124	80,180	47,529	53,635	21,140	20,631	17,248	17,045	9,219	17,647	50,754	53,903	6,762	28,475	8,086	39,411
65	TGAGGTAGTAGGTTGTGTGGTT	rno-let-7b-5p	17,563	35,333	36,476	7,347	19,385	17,499	13,469	8,298	18,459	25,152	13,418	16,422	7,077	7,029	5,648	5,563	2,789	5,584	19,698	17,507	2,025	10,136	2,698	14,033
66	TGAGGTAGTAGGTTGTATAGTT	rno-let-7a-5p	26,268	49,276	63,116	13,136	29,759	29,161	17,716	17,360	32,146	26,326	16,948	18,752	7,297	7,305	5,712	5,987	3,293	6,555	16,306	18,853	2,444	9,424	2,667	12,187
67	GCCCGGATAGCTCAG	rno-mir-760-p5_1ss10CG	666	499	743	103	401	370	261	76	376	292	158	196	54	69	51	79	38	126	160	147	25	78	28	302
68	CTGAGACTAACTCACCTGTCT	rno-mir-6331-p3	1,461	2,176	3,088	419	1,647	1,473	826	518	1,573	1,226	952	858	399	353	304	329	188	372	459	704	158	408	157	1,217
69	TCAGACAGCTGAGTCACTCTGC	rno-mir-6329-p3	10	20	20	4	12	10	2	5	9	11	4	8	3	2	0	0	0	3	9	6	3	0	0	5
70	TCTGCTGACTGCCTATGGGCT	rno-mir-6324-p5	27	42	58	9	26	24	19	11	25	29	16	23	4	3	6	2	7	2	13	15	2	4	0	18
71	AAGTGCTGAGATTACA	rno-mir-6320-p5	4	4	7	0	2	0	0	0	6	2	0	2	0	0	0	0	0	0	2	3	0	0	0	12
72	AGGCAGGAGGATCACGA	rno-mir-6320-p3_1ss15AC	44	44	32	2	39	21	40	8	21	34	15	23	2	8	10	19	0	14	28	24	0	10	2	26
73	TACATACACACATACACACAGA	rno-mir-466b-2-p3_2ss19TC21TG	3	6	21	0	8	4	4	4	9	8	0	6	3	2	5	0	0	0	3	0	0	2	0	3
74	TTTGTGTAGAGGAAGGCT	rno-mir-463-p3_1ss5GT	4	0	2	0	4	0	2	0	2	13	3	14	0	0	2	6	0	0	8	2	0	4	0	15
75	ACAGACATTAATTGGGCGCCTGA	rno-mir-421-p3_1ss23CA	599	1,069	1,371	279	592	669	397	261	609	564	426	440	199	172	134	148	83	173	250	398	84	279	80	298
76	AGACCAGGTCACGTCTCTGCAGA	rno-mir-370-p5_1ss23TA	92	161	238	42	84	86	44	61	110	71	39	39	7	17	9	16	4	16	23	62	7	25	5	40
77	GAAGGAGGCAGAGGC	rno-mir-346-p3_1ss12GA	3	8	6	0	17	3	5	2	4	25	7	15	2	6	4	10	2	0	20	13	0	4	0	29
78	AAGTCAGGCTCCTGGCTGGAGT	rno-mir-344i-p5	5	12	24	2	5	7	6	2	13	16	4	2	0	4	0	0	0	5	14	5	2	3	0	10
79	CTCTAGCCAGGGGCTTGACTGT	rno-mir-344g-p3_1ss22CT	12	21	23	3	15	10	6	0	7	20	10	13	10	2	2	3	2	2	11	12	3	8	0	2
80	CGGTCGGCCGATCGCTCGGTCTGTCAGTCAGTCGGTCGGT	rno-mir-341-p5	12	35	29	5	18	17	13	0	18	22	15	9	4	5	2	0	2	4	9	5	2	8	0	20
81	TCGATCGGTCGGTCGGTCAGT	rno-mir-341-p3	2,029	3,429	3,845	622	1,963	1,924	1,422	971	2,508	2,120	1,211	1,240	624	611	400	501	270	547	959	1,268	202	583	196	1,654
82	CGGGTCTTCCCGGAGTCGGG	rno-mir-320-p5_1ss4TG	7	0	10	2	13	4	0	0	0	14	0	6	4	4	2	2	2	4	15	4	2	4	0	17
83	GGTGGTGCAGGCAGGAGAGC	rno-mir-3102-p5	41	85	97	19	34	40	27	28	45	50	27	50	20	10	13	17	12	11	38	36	2	17	4	10
84	GAGCACCCCATTGGCTACCCACAGT	rno-mir-3102-p3_1ss25GT	359	1,214	800	193	422	364	342	243	505	553	393	439	156	164	113	99	82	115	396	394	66	237	84	226
85	AGGTGCCATTCTGAAGGCCAGGAGT	rno-mir-3085-p5	11	33	81	8	28	18	10	8	17	15	13	11	7	0	4	8	0	3	3	16	0	8	0	7
86	AAGCATCTGGCACCA	rno-mir-301b-p3_1ss11GC	523	999	889	124	301	151	195	185	436	193	101	195	116	75	24	52	18	75	87	115	6	46	13	470
87	CAAGCTCGTGTCTGTGGGTCCGT	rno-miR-99b-3p_R+1	104	192	288	30	120	102	67	64	118	134	65	76	28	19	16	19	17	27	66	74	8	17	15	81
88	CAAGCTCGTTTCTATGGGTCTGT	rno-miR-99a-3p_R+1	37	64	58	16	42	45	29	12	40	59	43	23	12	19	12	8	10	8	22	23	11	25	9	31
89	TGAGGTAGTAAGTTGTATTGTT	rno-miR-98-5p	4,729	9,435	12,283	2,948	6,388	6,001	3,613	3,504	7,343	4,479	3,493	3,531	1,428	1,404	1,069	1,147	635	1,361	2,488	3,297	556	1,638	517	2,343
90	CTATACAACTTACTACTTTCCT	rno-miR-98-3p_R+1	149	300	399	79	166	127	83	80	180	145	104	108	41	48	26	35	16	41	67	113	30	93	24	93
91	CAGTTACCGCTTCCGCTACCGC	rno-miR-935_L-1	110	179	202	37	118	104	83	43	119	107	91	78	39	32	29	32	16	30	88	79	9	47	19	100
92	CAAAGTGCTGTTCGTGCAGGTAG	rno-miR-93-5p	482	998	997	138	371	450	333	258	554	541	419	306	179	164	109	119	72	128	400	332	53	199	68	353
93	ACTGCTGAGCTAGCACTTCCCGA	rno-miR-93-3p	7	18	59	0	13	17	10	0	21	10	12	11	6	4	2	7	2	7	7	3	0	4	2	23
94	AGGGACGGGACGCGGTGCAGTGTT	rno-miR-92b-5p	6	9	25	2	6	15	9	3	10	3	7	2	0	5	0	2	2	6	4	6	0	2	3	3
95	TATTGCACTTGTCCCGGCCTGA	rno-miR-92a-3p_R+1	170	302	394	68	137	180	127	110	179	166	103	121	58	46	36	41	22	36	101	98	21	57	25	127
96	AGAGGCTTATAGCTCTAAGC	rno-miR-879-5p_R-1	709	1,089	1,396	205	657	712	394	257	673	632	547	431	191	183	133	160	101	168	245	441	69	167	68	485
97	CTTATGGCTTCAAGCTTTCGGT	rno-miR-879-3p_R+1	56	72	84	8	40	47	20	14	61	41	23	27	22	11	12	17	6	15	17	21	6	21	7	25
98	GTAGAGGAGATGGCGCAGGGGA	rno-miR-877_R+2	14	50	52	10	17	15	14	8	27	20	15	20	0	4	7	0	0	3	11	15	0	14	2	2
99	TGTCCTCTTCTCCCTCCTCCCA	rno-miR-877-3p	23	55	65	15	34	24	23	10	31	42	24	26	6	9	11	5	4	9	8	21	4	7	3	29
100	TGGATTTCTCTGTGAATCACC	rno-miR-876_R-1	75	144	226	39	118	112	37	45	119	63	50	41	36	23	21	22	17	30	21	47	20	42	15	64

101	CGGCCCCACGCACCAGGGTAAGA	rno-miR-874-5p_R+2	20	35	68	7	21	22	11	2	24	11	16	8	4	4	2	6	0	9	4	15	0	5	2	15
102	CTGCCCTGGCCCGAGGGACCGAC	rno-miR-874-3p_R+1	1,751	4,918	5,551	1,100	2,398	2,482	1,565	1,251	2,578	1,754	1,323	1,470	727	674	521	508	458	635	1,090	1,604	296	953	334	1,544
103	TGCAGGAACTTGTGAGTCTCC	rno-miR-873-5p_L+1R-1	2,028	3,285	4,556	1,039	2,363	1,893	1,315	823	2,319	1,828	1,151	1,361	436	469	363	465	307	500	672	1,172	215	623	189	1,307
104	GAGACTGACAAGTTCCCGGGA	rno-miR-873-3p	627	756	1,195	293	556	389	331	180	772	605	371	398	109	105	102	79	69	137	161	252	34	186	39	203
105	AAGGTTACTTGTTAGTTCAGG	rno-miR-872-5p	908	1,507	1,866	312	959	979	615	478	903	778	617	617	272	261	189	220	120	266	533	592	114	284	103	699
106	TGAACTATTGCAGTAGCCTCCT	rno-miR-872-3p	1,003	1,975	1,960	342	947	1,005	664	388	938	952	799	674	350	292	246	213	153	239	526	623	110	411	130	719
107	TCAGTAACAAAGATTCATCCTT	rno-miR-802-5p	14	45	18	9	23	34	12	0	22	17	10	7	7	4	0	0	3	4	7	17	0	6	0	10
108	TGGAAGACTTGTGATTTTGTTGTT	rno-miR-7b	3,268	5,577	17,516	2,479	4,675	4,504	2,248	3,018	4,653	2,910	1,842	2,077	750	1,289	762	690	523	1,329	1,822	2,301	486	1,350	338	1,178
109	CAACAAGTCCCAGTCTGCCACA	rno-miR-7a-2-3p	74	111	227	23	72	56	59	40	93	97	50	56	14	27	10	15	15	22	47	47	3	21	8	64
110	CAACAAATCACAGTCTGCCATA	rno-miR-7a-1-3p	245	511	566	95	269	229	151	140	353	270	259	178	101	103	67	104	37	79	188	198	37	107	43	189
111	AGCACCACGTGTCTGGGCCACGT	rno-miR-770-5p	178	301	347	80	228	182	149	64	150	239	147	137	90	75	73	64	27	64	110	199	32	125	31	543
112	CGTGGGCCTGACGTGGAGCTGG	rno-miR-770-3p	139	226	352	61	168	155	100	56	143	197	92	132	34	41	21	29	6	36	89	117	10	41	13	120
113	GGTGCTCACATGTCCTCCTCCA	rno-miR-764-5p	4	6	61	0	10	3	0	8	2	2	0	0	2	2	0	0	0	0	2	2	0	0	0	5
114	GAGGAGGCCATAGTGGCAACTGT	rno-miR-764-3p	24	34	137	11	23	18	11	15	25	17	17	15	5	7	4	4	0	8	2	5	0	4	0	5
115	CGGCTCTGGGTCTGTGGGGAGT	rno-miR-760-3p	204	405	510	87	204	177	120	93	191	289	145	209	74	43	45	44	36	48	119	170	15	79	23	166
116	TTTGTGACCTGGTCCACTAACCC	rno-miR-758-3p	562	952	1,302	199	561	687	340	292	650	444	344	329	211	149	152	149	95	163	209	376	87	276	75	486
117	GAAAGACACCATACTGAATAGA	rno-miR-743b-3p	0	15	0	0	0	0	0	0	0	0	0	0	0	0	0	0	0	4	0	3	0	12	0	0
118	AAAGATGCCACGCTATGTAGA	rno-miR-741-3p	0	10	0	0	0	2	0	0	0	0	0	0	0	0	0	0	0	3	0	0	0	10	0	0
119	AAGGAGCTTACAATCTAGCTGG	rno-miR-708-5p	885	1,695	2,630	318	960	1,011	560	472	1,025	961	643	588	284	349	245	317	165	381	772	698	148	376	131	695
120	CAACTAGACTGTGAGCTTCTAGT	rno-miR-708-3p	793	1,524	2,576	401	893	847	512	453	864	731	593	517	211	223	168	172	139	210	551	496	104	269	96	430
121	TGCCCACCCTTTACCCCACTCCAGT	rno-miR-702-3p	250	506	491	138	278	241	182	176	310	305	207	227	77	61	75	62	46	45	126	164	44	123	49	103
122	GCACTGAGATGGGAGTGGTGT	rno-miR-674-5p	505	856	922	208	518	497	317	185	530	472	252	272	158	146	110	164	67	136	208	312	51	202	55	463
123	CACAGCTCCCATCTCAGAAC	rno-miR-674-3p	976	1,583	1,603	413	942	841	615	436	962	752	558	558	306	256	199	274	146	228	403	726	105	374	124	888
124	CTCACAGCTCCGGTCCTTGGAGC	rno-miR-673-5p	67	204	171	38	127	107	88	28	85	119	95	119	40	29	37	20	26	19	66	84	30	41	23	115
125	TCCGGGACTGAGTTCTGTGCACC	rno-miR-673-3p	237	484	682	114	249	235	141	168	357	156	132	146	54	66	45	58	27	49	100	171	39	93	26	112
126	TGAGGTTGGTGTACTGTGTGTGA	rno-miR-672-5p	238	438	594	121	215	195	109	133	221	230	96	117	41	49	34	36	19	52	181	120	16	86	19	110
127	ACACACAGTCGCCATCTTCGA	rno-miR-672-3p	247	438	685	138	310	352	190	122	290	274	220	206	99	70	67	45	47	84	148	171	31	118	36	272
128	AGGAAGCCCTGGAGGGGCTGGAGG	rno-miR-671-5p	98	162	215	35	98	73	42	50	96	90	78	71	22	19	11	30	5	36	46	63	14	53	5	53
129	TCCGGTTCTCAGGGCTCCACC	rno-miR-671	378	568	960	217	350	353	192	240	426	184	151	144	63	75	64	61	44	69	111	206	24	96	26	300
130	TGTCACTCGGCTCGGCCCACTACC	rno-miR-668	483	931	1,167	310	565	586	323	354	611	379	251	242	196	164	116	122	90	131	213	376	81	242	60	344
131	CGGTGCTGGTGGAGCAGTGAGCAC	rno-miR-667-5p	11	24	34	0	17	14	7	13	12	11	10	16	2	4	0	0	4	0	7	17	2	4	0	3
132	TGACACCTGCCACCCAGCCCAAG	rno-miR-667-3p	1,377	2,190	2,720	636	1,521	1,257	806	685	1,729	1,037	665	886	356	328	288	250	192	324	410	798	169	497	207	891
133	AGCGGGCACGGCTGTGAGAGC	rno-miR-666-5p	25	56	76	11	32	33	13	14	27	25	15	15	6	8	0	2	5	2	16	18	0	5	2	7
134	ACCAGGAGGCTGAGGTCCCTTA	rno-miR-665	599	1,068	1,388	204	704	613	339	317	669	471	358	353	145	135	133	117	74	131	165	316	67	156	59	357
135	TATTCATTTACTCCCCAGCCT	rno-miR-664-3p	1,288	3,188	2,549	507	1,525	1,621	934	804	1,680	1,815	1,222	1,337	468	385	373	338	305	321	773	1,291	122	710	252	773
136	CTGGCTGGGGAAAATGATTGGA	rno-miR-664-2-5p	316	532	589	116	315	289	177	166	297	298	156	187	85	74	53	50	39	67	113	190	7	104	20	89
137	AATGGCGCCACTAGGGTTGTGT	rno-miR-652-3p	1,105	2,297	2,537	542	1,328	1,328	786	441	1,281	1,693	1,105	1,064	537	461	374	387	257	381	1,010	982	171	629	184	954
138	CTTTGGTGGCTTAGTTCTTTGT	rno-miR-6331	567	956	1,335	264	601	557	398	383	671	469	293	428	126	145	99	113	62	145	195	379	58	192	72	389
139	AAAGTCAGACAGAGACTCTGCT	rno-miR-6325	3	20	34	0	7	11	3	3	3	16	20	11	3	6	0	4	2	2	8	9	0	7	0	4
140	CTGCCTGGCGCAGGGCCTGTAGT	rno-miR-6318	10	36	22	2	21	17	10	8	24	11	4	11	7	6	5	2	2	0	12	11	0	5	2	13
141	ATTAGGGTTGCAGAGCC	rno-miR-6215	2,191	1,857	1,697	353	2,260	2,332	1,546	275	1,496	1,745	1,232	1,251	618	838	638	905	381	1,072	1,686	1,099	96	682	212	2,161
142	TACGTCATCGTCGTCATCGTTAT	rno-miR-598-3p	2,124	4,070	5,605	961	2,284	2,390	1,347	1,095	2,957	2,348	1,800	1,968	547	477	370	577	317	487	798	1,477	286	714	598	1,892
143	TTGTGTCAATATGCGATGATGT	rno-miR-592	166	416	467	79	246	313	121	108	299	242	161	134	67	70	45	51	29	65	138	137	18	72	24	177
144	ATACAGTTGTTCAACCAGTTAC	rno-miR-582-5p	1,143	1,932	2,071	388	1,333	1,341	692	458	1,264	1,108	851	857	380	302	238	255	194	279	425	675	145	481	149	736
145	AACCTGTTGAACAACTGAACCC	rno-miR-582-3p	1,066	1,566	1,934	403	996	1,191	618	521	1,260	680	603	621	335	241	232	275	186	252	337	552	167	390	103	500
146	GAAATCAAGCTTGGGTGAGACCT	rno-miR-551b-5p	13	32	58	7	21	20	13	8	23	19	10	5	8	7	3	4	2	6	12	11	0	13	2	8
147	GCGACCCATACTTGGTTTCAGT	rno-miR-551b-3p	195	318	931	62	203	222	123	151	327	172	103	141	43	69	28	48	13	89	62	74	12	53	22	95
148	ATTGGTACTTCTTTAAGTGAGA	rno-miR-547-3p	3	25	23	3	22	12	5	2	6	12	4	5	2	4	3	3	2	0	5	4	3	6	2	8
149	TCTTGTTAAAAAGCAGATTCT	rno-miR-544-5p	16	31	22	6	14	22	16	9	15	22	17	15	8	7	10	14	3	3	7	17	4	8	8	17
150	ATTCTGCATTTTTAGCAAGCTT	rno-miR-544-3p	27	55	66	12	34	25	14	15	29	31	19	21	7	6	2	6	0	3	5	21	3	3	5	11
151	GAAGTTGCCCGCGTGTTTTTCG	rno-miR-543-5p	19	31	37	11	22	23	15	6	28	23	10	13	0	2	2	3	5	0	5	16	3	9	4	10
152	AAACATTCGCGGTGCACTTCTT	rno-miR-543-3p	545	1,180	1,992	268	746	666	470	386	884	778	452	612	287	335	241	279	167	291	439	935	157	385	144	411
153	CTCGGGGATCATCATGTCACGA	rno-miR-542-5p	9	9	22	3	10	15	4	4	10	11	11	7	2	0	6	2	0	5	3	5	2	0	0	6
154	TGTGACAGATTGATAACTGAAAGT	rno-miR-542-3p	231	661	1,045	180	349	376	190	203	367	392	285	190	69	76	63	49	38	61	159	198	46	109	30	123

155	AAGGGATTCTGATGTTGGTCACACT	rno-miR-541-5p	3,389	7,064	8,011	1,876	4,008	3,091	2,312	1,802	4,007	2,427	2,188	2,252	1,708	1,279	1,115	973	849	1,198	1,884	4,590	842	2,028	570	2,232
156	TGGCGAACACAGAATCCATACT	rno-miR-541-3p	161	471	542	128	255	265	169	152	288	213	153	218	88	72	77	67	55	73	109	194	47	119	39	124
157	CAAGGGTCACCCTCTGACTCT	rno-miR-540-5p	84	137	115	31	72	75	71	26	65	129	83	86	28	31	20	15	16	19	41	56	6	30	14	115
158	AGGTCAGAGGTCGATCCTGGGC	rno-miR-540-3p	82	156	144	31	72	64	73	36	81	108	58	71	29	20	22	16	17	20	48	80	17	35	12	45
159	GGAGAAATTATCCTTGGTGTGTT	rno-miR-539-5p	254	509	749	131	364	347	228	232	392	350	220	272	89	100	54	72	37	83	150	229	57	158	44	142
160	CATACAAGGGTAATTTCTTTTC	rno-miR-539-3p	384	676	839	124	432	344	245	206	418	299	223	233	143	142	81	111	45	103	149	274	60	199	51	264
161	CATGCCTTGAGTGTAGGACTGT	rno-miR-532-5p	246	490	506	98	233	210	160	105	253	423	302	241	81	85	68	64	49	87	232	190	30	106	53	236
162	CCTCCCACACCCAAGGCTTGC	rno-miR-532-3p	43	59	91	12	36	25	21	26	53	36	24	26	10	8	9	7	8	13	14	26	8	14	5	20
163	GGGAGCCAGGAAGTATTGATGTT	rno-miR-505-5p	21	74	96	14	58	40	19	23	35	37	25	29	20	16	13	7	3	13	21	26	8	15	0	10
164	CGTCAACACTTGCTGGTTTCCT	rno-miR-505-3p	317	661	836	149	348	391	215	198	357	446	327	322	149	141	101	81	52	88	333	268	65	189	59	283
165	GACCCTGGTCTGCACTCTGTC	rno-miR-504	523	1,039	1,026	188	483	474	358	236	501	781	345	432	155	172	142	133	78	136	331	401	64	210	95	487
166	GGAGTATTGTTTCCGCTGCCTGG	rno-miR-503-3p	54	84	110	15	56	51	42	21	38	88	40	52	18	13	9	6	8	14	23	31	4	14	7	22
167	AATGCACCCGGGCAAGGATTTT	rno-miR-501-3p	62	171	173	35	53	66	34	42	93	70	61	72	34	22	17	5	10	24	71	94	15	43	9	38
168	AATGCACCTGGGCAAGGGTTC	rno-miR-500-3p	38	107	125	17	46	45	26	34	62	56	29	28	17	12	6	8	0	16	32	27	2	13	11	21
169	TTAAGACTTGCAGTGATGTTT	rno-miR-499-5p	823	1,326	2,455	387	910	1,111	497	416	934	599	460	452	191	166	127	176	94	244	301	390	87	237	84	411
170	GAACATCACAGCAAGTCTGTGC	rno-miR-499-3p	19	41	54	9	13	49	19	3	17	37	25	13	11	4	10	5	9	13	12	28	4	2	3	21
171	CAGCAGCACACTGTGGTTTGT	rno-miR-497-5p	314	675	615	76	282	346	183	106	318	416	266	262	121	108	89	92	50	59	241	224	46	159	43	357
172	AGTATTACATGGCCAATCTCC	rno-miR-496-3p	1,779	3,207	2,533	559	1,802	1,547	1,119	1,141	2,567	760	557	633	502	397	312	372	166	411	373	777	215	538	175	515
173	AAACAAACATGGTGCACTTCTT	rno-miR-495	885	1,680	1,937	319	1,090	843	589	621	1,356	789	557	613	314	298	195	249	122	248	390	652	124	364	109	314
174	TGAAACATACACGGGAAACCTCT	rno-miR-494-3p	10	16	19	5	7	14	5	6	14	8	15	6	4	0	4	8	0	2	8	8	3	9	0	18
175	TTGTACATGGTAGGCTTTCATT	rno-miR-493-5p	256	448	1,080	123	315	319	148	179	363	258	166	193	67	75	48	52	31	74	115	189	37	75	28	153
176	TGAAGGTCTACTGTGTGCCAGG	rno-miR-493-3p	87	166	246	46	91	99	58	43	111	64	37	65	19	25	12	15	13	21	50	64	7	31	7	46
177	CCATGGATCTCCAGGTGGGT	rno-miR-490-5p	36	108	57	14	42	32	30	11	44	74	48	43	15	12	11	13	9	13	60	31	2	9	0	46
178	CAACCTGGAGGACTCCATGCTGT	rno-miR-490-3p	3,008	8,830	7,138	1,636	3,235	3,165	1,764	1,669	4,302	2,499	2,139	1,765	696	659	548	674	460	593	2,288	2,233	386	938	314	1,423
179	CCCAGATAATGGCACTCTCAAAC	rno-miR-488-5p	111	179	319	39	77	86	49	40	78	71	61	50	27	26	19	11	9	12	54	58	7	41	8	37
180	TTGAAAGGCTGTTTCTTGGTC	rno-miR-488-3p	6,562	11,348	15,852	3,277	6,552	7,193	3,568	3,349	5,570	5,337	3,894	3,641	1,539	1,250	1,242	1,177	880	1,448	2,558	4,110	795	2,146	651	2,662
181	GTGGTTATCCCTGTCCTCTTCG	rno-miR-487b-5p	22	11	22	2	17	10	6	0	16	15	5	8	4	0	0	2	0	0	0	6	0	2	0	7
182	AATCGTACAGGGTCATCCACTC	rno-miR-487b-3p	3,722	6,824	6,994	1,780	4,075	3,904	3,058	1,873	4,187	3,671	2,787	2,921	1,290	1,246	1,058	920	596	1,109	1,889	2,868	541	1,565	443	2,525
183	AGAGGCTGGCCGTGATGAATTCG	rno-miR-485-5p	932	1,538	1,469	432	956	795	709	386	897	903	556	724	346	242	227	205	114	203	397	676	78	382	101	378
184	CATACACGGCTCTCCTCTCTTC	rno-miR-485-3p	258	457	780	104	271	239	186	158	345	288	169	191	90	101	67	91	62	54	180	224	25	121	63	249
185	TCAGGCTCAGTCCCCTCCCGAT	rno-miR-484	196	389	448	59	159	185	114	95	210	138	92	97	59	61	59	48	23	37	108	78	21	78	26	205
186	CACTCCTCCCCTCCCGTCTTGT	rno-miR-483-3p	4	13	14	3	8	5	2	0	11	30	12	6	0	2	2	0	0	0	4	6	0	2	0	5
187	TATGTGCCTTTGGACTACATC	rno-miR-455-5p	33	62	96	12	40	48	13	17	42	33	30	10	10	11	5	7	0	4	21	18	9	9	7	35
188	GCAGTCCACGGGCATATACACC	rno-miR-455-3p	61	188	390	82	189	275	43	87	188	274	164	179	77	60	60	49	42	49	133	140	32	116	27	105
189	AAACCGTTACCATTACTGAGTTT	rno-miR-451-5p	474	922	779	211	967	467	373	236	805	1,326	684	808	263	161	75	278	70	134	323	413	87	399	105	774
190	TTTTGCGATGTGTTCCTAATAT	rno-miR-450a-5p	2,340	4,654	6,145	1,077	3,086	3,473	1,711	1,081	2,554	3,030	2,103	1,768	1,006	708	689	535	412	684	1,324	1,587	349	1,011	327	1,426
191	TTGCATATGTAGGATGTCCCT	rno-miR-448-3p	31	73	235	16	27	40	29	47	43	50	23	19	8	25	9	2	5	18	14	18	6	14	10	23
192	TACGGTGAGCCTGTCATTATT	rno-miR-433-5p	235	358	584	86	217	202	115	108	268	190	122	117	50	53	47	50	31	49	93	139	31	74	24	102
193	ATCATGATGGGCTCCTCGGTGT	rno-miR-433-3p	1,147	1,907	2,627	509	1,180	1,075	808	602	1,268	1,311	703	990	420	344	283	317	188	303	665	1,045	147	467	179	1,046
194	TGTCTTGCAGGCCGTCATGC	rno-miR-431	103	138	154	22	92	96	58	45	93	59	42	39	22	21	13	18	8	33	26	33	8	17	4	76
195	CAGGTCGTCTTGCAGGGCTTCT	rno-miR-431-3p	353	620	682	134	326	328	246	170	331	551	306	311	165	122	92	97	63	108	238	292	67	176	47	298
196	AATGACACGATCACTCCCGTTG	rno-miR-425-5p	798	1,670	2,149	380	944	928	580	392	957	883	741	620	335	329	285	236	195	284	573	707	147	433	156	738
197	CATCGGGAATATCGTGTCCGCC	rno-miR-425-3p	64	164	141	15	59	47	29	16	96	53	34	28	26	35	15	34	14	25	45	52	19	36	11	56
198	TGAGGGGCAGAGAGCGAGACTTT	rno-miR-423-5p	509	1,287	1,136	302	640	559	370	401	619	755	558	537	180	154	136	101	50	118	325	428	64	253	27	103
199	AGCTCGGTCTGAGGCCCCTCAGT	rno-miR-423-3p	1,484	2,327	3,544	670	1,171	1,299	755	954	1,493	1,224	849	756	313	299	259	246	135	251	556	634	153	445	174	937
200	TGGTCGACCAGCTGGAAAGT	rno-miR-412-5p	215	380	399	108	244	217	163	118	243	181	134	152	64	52	44	32	30	66	79	169	24	82	16	95
201	TACTTCACCTGGTCCACTAGC	rno-miR-412-3p_L+2R-3	31	53	96	14	34	30	31	14	41	32	19	17	6	8	2	7	4	8	7	21	0	14	4	31
202	ATAGTAGACCGTATAGCGTACG	rno-miR-411-5p_L+1	3,538	5,048	5,973	1,252	3,427	3,075	2,024	1,612	3,555	2,198	1,644	1,733	875	785	683	692	420	738	1,088	1,946	318	977	299	1,310
203	TATGTAACACGGTCCACTAAC	rno-miR-411-3p_R+1	204	367	431	71	239	225	127	111	336	163	104	142	94	75	65	80	33	83	90	148	27	102	28	167
204	AATATAACACAGATGGCCTGT	rno-miR-410-3p	3,743	5,402	6,293	1,191	3,571	3,606	2,423	1,365	3,858	3,376	2,251	2,315	1,086	827	771	846	470	972	1,326	2,084	391	1,155	394	2,001
205	GAATGTTGCTCGGTGAACCCCT	rno-miR-409a-3p_L+1R+1	1,532	2,762	4,134	713	1,674	1,715	1,152	859	1,934	1,523	983	1,167	620	670	421	439	298	543	918	1,446	266	873	225	1,155
206	AGATCAGAAGGTGACTGTGGCT	rno-miR-383-5p_L-1R+2	4,884	9,881	4,033	1,604	2,918	3,262	4,189	1,865	2,550	3,761	2,797	2,858	1,332	1,146	1,074	865	583	972	2,002	3,082	340	1,832	493	3,362

207	CCACAGCACTGCCTGGTCAGA	rno-miR-383-3p	18	21	15	3	14	6	7	3	15	12	4	9	2	2	2	3	0	2	7	3	0	2	0	12
208	GAAGTTGTTCGTGGTGGATTCG	rno-miR-382-5p	204	305	496	106	284	218	123	139	297	198	154	195	105	90	78	82	47	92	130	230	41	120	44	571
209	AATCATTCACGGACAACACTTT	rno-miR-382-3p_R+1	177	331	396	58	199	176	111	74	233	172	112	120	73	81	61	75	37	80	117	157	33	96	25	141
210	TATACAAGGGCAAGCTCTCTGT	rno-miR-381-3p_R+4	2,971	5,304	6,001	1,089	2,980	2,780	2,014	1,270	2,922	2,875	1,893	2,176	925	918	694	703	424	718	1,511	2,312	389	1,108	396	2,206
211	ATGGTTGACCATAGAACATGC	rno-miR-380-5p_R-1	149	259	456	71	155	131	103	100	168	154	123	107	21	56	33	41	22	38	83	111	19	44	16	87
212	TATGTAGTATGGTCCACATCT	rno-miR-380-3p	1,383	2,216	2,857	468	1,545	1,487	875	577	1,487	1,095	897	782	459	374	370	333	213	422	466	745	179	475	152	1,013
213	TGGTAGACTATGGAACGTAGG	rno-miR-379-5p	2,298	3,768	5,601	1,305	3,200	2,494	1,718	1,285	3,252	2,127	1,527	1,867	880	992	647	942	578	1,082	1,504	2,477	439	1,045	373	2,190
214	TATGTAACATGGTCCACTAAC	rno-miR-379-3p_L-1	374	769	814	186	464	394	300	204	512	319	252	297	166	164	124	160	79	144	219	405	82	235	76	361
215	ACTGGACTTGGAGTCAGAAGGT	rno-miR-378b_R+1_1ss2GC	80	128	182	24	71	67	31	35	81	57	42	28	16	15	9	13	5	14	34	33	6	19	5	30
216	CTCCTGACTCCAGGTCCTGTGT	rno-miR-378a-5p	46	97	237	28	97	96	38	35	101	96	73	67	25	13	16	15	9	19	45	44	9	33	13	65
217	ACTGGACTTGGAGTCAGAAGGC	rno-miR-378a-3p_R+1	4,633	10,243	13,220	2,292	4,930	4,553	2,749	2,945	6,229	4,873	3,243	2,670	1,195	1,179	956	1,053	635	954	2,926	3,188	647	1,683	517	2,579
218	TGAATCACACAAAGGCAACTTTT	rno-miR-377-3p	226	679	796	148	404	314	191	167	353	255	207	258	133	95	112	75	84	96	167	328	78	185	54	197
219	AACATAGAGGAAATTTCACGT	rno-miR-376c-3p	137	243	321	59	193	148	86	84	218	134	121	86	52	38	37	39	30	47	55	80	16	67	17	56
220	GTGGATATTCCTTCTATGGTT	rno-miR-376b-5p_R-1	477	635	1,253	207	619	475	285	248	630	328	290	341	221	159	135	160	88	179	210	361	77	172	61	320
221	ATCATGGAGGAACATCCACTT	rno-miR-376b-3p_1ss6AG	2,358	3,641	4,299	710	2,391	2,248	1,532	777	2,465	1,977	1,412	1,405	844	864	632	742	446	782	1,089	1,594	333	866	240	1,870
222	GGTAGATTCTCCTTCTATGAGT	rno-miR-376a-5p_R+1	519	900	1,206	216	589	638	292	239	644	358	292	274	142	135	89	107	74	95	166	289	69	174	49	250
223	ATCGTAGAGGAAAATCCACGTT	rno-miR-376a-3p_R+1	256	374	836	65	291	269	152	127	344	236	171	126	67	61	50	67	31	68	66	107	30	62	23	132
224	TTTGTTCGTTCGGCTCGCGTGA	rno-miR-375-3p	26	70	43	12	98	16	15	12	31	20	35	29	18	4	7	62	6	5	74	155	2	8	8	28
225	ATATAATACAACCTGCTAAGT	rno-miR-374-5p_R-1	893	1,952	2,460	451	1,266	1,218	605	497	1,133	1,253	868	780	289	340	274	273	186	339	633	633	170	444	200	1,153
226	CTTAGCACGTTGTATTATTATT	rno-miR-374-3p	490	883	1,390	284	654	599	237	250	547	277	195	221	161	141	100	130	85	145	202	266	71	218	58	157
227	GCCTGCTGGGGTGGAACCTGGT	rno-miR-370-3p	1,338	2,366	2,488	725	1,521	1,305	1,015	620	1,284	1,241	875	981	469	375	331	273	240	386	625	1,102	205	620	121	978
228	AGATCGACCGTGTTATATTCGC	rno-miR-369-5p	2,427	4,520	5,417	1,080	2,951	2,672	1,637	1,366	2,843	1,443	1,072	1,422	792	595	552	532	350	631	847	1,519	293	917	283	934
229	AATAATACATGGTTGATCTTT	rno-miR-369-3p	1,350	2,253	3,533	507	1,385	1,328	1,099	826	1,927	1,457	1,004	1,061	598	658	437	518	254	611	880	1,293	277	697	206	917
230	TAATGCCCCTAAAAATCCTTAT	rno-miR-365-3p	887	2,169	2,636	484	1,156	1,387	660	622	1,303	1,161	816	808	343	305	241	237	217	242	543	685	165	546	220	525
231	AATTGCACGGTATCCATCTGTAA	rno-miR-363-3p_R+2	0	11	12	2	6	6	4	0	0	8	0	13	0	0	0	0	0	6	26	7	0	4	0	4
232	AATCCTTGGAACCTAGGTGTGA	rno-miR-362-5p_R-2	22	51	64	11	38	40	16	10	33	32	15	23	28	20	24	17	7	16	41	36	8	22	9	26
233	AACACACCTGTTCAAGGATTCT	rno-miR-362-3p_1ss22AT	27	86	87	16	44	49	29	16	67	69	53	32	18	20	12	5	7	12	37	27	12	13	7	26
234	TTATCAGAATCTCCAGGGGTAC	rno-miR-361-5p	1,108	3,532	4,488	1,225	2,277	2,186	1,215	996	1,879	2,287	1,528	1,724	517	601	441	430	352	586	1,224	1,443	316	783	302	1,020
235	CCCCCAGGTGTGATTCTGATTCGT	rno-miR-361-3p	859	1,452	1,514	351	631	722	537	405	769	650	511	429	218	194	204	149	114	197	334	472	107	319	98	450
236	TTCACACCGCCTCTGCCCGCT	rno-miR-3594-3p_L+2R-3	11	14	28	8	7	9	6	3	14	11	3	8	4	0	3	2	3	2	5	4	0	4	3	12
237	TCTGTCCCTCTTGGCCCTTAGT	rno-miR-3577_R+1	97	141	227	52	91	98	48	64	100	60	23	25	25	17	16	10	8	12	29	45	7	17	9	118
238	TGACAGACTTAGTACTACATGA	rno-miR-3559-5p	52	82	147	15	39	40	31	25	45	46	25	21	13	18	6	2	4	10	37	20	8	9	4	20
239	AGGCTGCAGGCCCACTTCCCT	rno-miR-3552	6	12	36	3	6	5	4	6	6	12	6	5	0	6	5	2	0	8	0	0	0	7	2	18
240	TGAGCACCACCCCTCTCTCAGT	rno-miR-3547_R-1_1ss22AT	18	29	56	5	23	14	20	11	23	9	9	5	3	5	3	0	0	4	6	10	2	6	0	9
241	TCCCTGAGGAGCCCTTTGAGCCT	rno-miR-351-5p_R-2	2,631	6,329	6,497	1,523	2,830	3,098	2,105	1,520	2,860	3,718	2,748	2,443	994	832	806	542	480	786	1,462	2,152	444	1,361	401	1,689
242	GGTCAAGAGGCGCCTGGGAAC	rno-miR-351-3p	10	18	26	2	7	5	7	2	6	0	3	16	0	3	5	0	0	3	4	5	0	6	0	5
243	TTCACAAAGCCCATACACTTTC	rno-miR-350_R-2	50	137	246	26	86	97	52	61	122	94	69	64	18	19	21	20	17	27	47	51	9	27	9	31
244	AAAGTGCACGTGCTTTGGGAC	rno-miR-350-5p_R+2_2ss9TC11CT	10	14	16	3	2	3	8	2	9	10	8	10	0	0	0	3	0	0	3	7	2	8	0	10
245	AGGCAGTGTAGTTAGCTGATTGC	rno-miR-34c-5p	1,293	2,592	4,463	677	1,633	1,691	811	1,193	2,103	1,109	739	858	380	373	285	280	176	395	743	998	203	457	200	512
246	AATCACTAACCACACAGCCAGG	rno-miR-34c-3p	7	13	22	3	8	13	0	7	14	11	6	5	3	5	2	2	0	7	12	10	0	4	2	4
247	AATCACTAACTCCACTGCCATC	rno-miR-34b-3p	19	45	90	7	33	20	16	16	44	38	16	15	11	7	7	7	2	6	9	14	7	13	2	15
248	TGGCAGTGTCTTAGCTGGTTGT	rno-miR-34a-5p	749	1,653	1,516	278	864	768	570	543	1,424	623	501	610	283	244	221	310	125	191	259	645	103	269	101	394
249	GGGCTGGAGAGATGGCT	rno-miR-3473_L-4R-1	8	9	18	0	4	3	4	0	14	29	13	10	7	4	2	6	2	2	47	10	0	5	0	23
250	TGTCTGCCTGAGTGCCTGCCTCT	rno-miR-346	569	828	1,227	254	529	515	344	349	612	414	272	303	144	141	116	111	67	114	226	280	66	198	52	449
251	TGCTGACCCCTAGTCCAGTGCT	rno-miR-345-5p_R+1	132	356	294	42	165	131	150	75	152	296	222	201	66	97	67	67	48	41	165	129	31	122	32	228
252	CCCTGAACTAGGGGTCTGGAGT	rno-miR-345-3p_1ss22AT	96	185	290	52	112	123	55	51	108	169	123	89	42	38	27	31	31	38	110	84	23	77	17	111
253	CTCTAGCCAGGGCTTGACTGC	rno-miR-344i_R-1	10	12	31	3	11	6	4	0	12	6	10	4	2	6	2	2	0	0	13	10	2	0	2	9
254	AGTCAGGCTGCTGGTTATATTCC	rno-miR-344b-5p_R+1	160	277	294	45	128	114	69	86	139	102	57	72	28	24	24	23	19	22	56	90	15	42	20	77
255	GGTATAACCAAAGCCCGACTGT	rno-miR-344b-2-3p	256	457	534	93	255	257	156	119	350	262	179	148	72	84	53	67	41	60	85	137	49	105	29	168
256	GATATAACCAAAGCCCGACTGT	rno-miR-344b-1-3p	1,317	2,076	1,794	663	1,074	1,248	923	592	1,266	1,340	1,093	902	335	312	286	228	202	280	652	605	136	464	139	508
257	TGATCTAGCCAAAGCCTGACTGT	rno-miR-344a-3p_1ss21CT	405	863	1,125	153	492	506	294	226	541	567	347	322	135	198	170	164	103	143	349	393	80	228	71	236

258	AGGGGTGCTATCTGTGATTGA	rno-miR-342-5p_R-1	94	177	198	36	97	88	50	33	81	79	69	58	21	33	23	13	11	14	41	48	8	41	2	46
259	TCCCTGTCCTCCAGGAGCTC	rno-miR-339-5p_R-3	128	238	230	34	116	118	73	31	132	185	121	116	37	46	40	42	15	35	74	75	22	60	20	121
260	TGAGCGCCTCGACGACAGAGC	rno-miR-339-3p_R-2	90	216	193	49	106	114	62	37	115	117	82	70	34	17	18	25	12	26	52	55	2	34	10	51
261	AACAATATCCTGGTGCTGAGT	rno-miR-338-5p_R-1	2,041	4,283	5,213	778	2,158	2,208	1,821	1,558	2,540	1,492	882	1,309	799	897	532	503	279	766	1,068	1,695	223	707	232	1,124
262	TCCAGCATCAGTGATTTTGTT	rno-miR-338-3p_R-2	2,944	6,239	10,454	1,323	3,785	3,991	2,624	2,587	5,049	2,437	2,040	2,235	1,054	1,383	780	829	455	1,301	1,538	2,231	443	1,087	392	1,908
263	CGGCGTCATGCAGGAGTTGATT	rno-miR-337-5p_R+1	616	1,104	1,145	279	703	664	554	365	845	729	486	565	186	211	147	159	106	187	257	498	82	301	84	494
264	TTCAGCTCCTATATGATGCCT	rno-miR-337-3p_R-2	89	189	242	31	97	116	84	51	115	112	88	87	26	42	12	25	21	20	55	61	11	35	8	63
265	TCTAGGTATGGTCCCAGGGATC	rno-miR-331-5p_L+1R-1	68	176	188	31	95	104	66	36	84	92	43	56	29	34	23	24	17	32	55	77	12	35	9	62
266	GCCCCTGGGCCTATCCTAGAAT	rno-miR-331-3p_R+1	1,181	2,689	2,225	387	1,193	1,312	893	590	1,386	1,949	1,053	1,087	484	542	364	345	242	352	862	986	237	678	241	864
267	TCTCTGGGCCTGTGTCTTAGGCT	rno-miR-330-5p_R+1	1,704	3,021	3,940	592	1,782	1,611	1,039	851	1,734	1,596	1,023	1,075	466	433	384	375	252	455	695	1,112	220	629	231	1,460
268	CAAAGCACAGGGCCTGCAGAGA	rno-miR-330-3p_L-1	1,689	3,690	4,741	809	1,823	1,916	1,047	1,019	1,689	1,400	883	1,121	562	480	376	414	240	384	705	1,302	199	741	236	760
269	GTGCATTGTAGTTGCATTGC	rno-miR-33-5p_R-1	167	165	518	42	148	134	58	74	187	139	87	81	24	34	22	43	15	42	45	49	17	22	7	101
270	CAATGTTTCCACAGTGCATCA	rno-miR-33-3p	25	39	81	11	27	23	21	18	45	13	24	24	5	6	5	8	2	2	19	19	0	7	2	34
271	AGAGGTTTTCTGGGTCTCTGTTT	rno-miR-329-5p_R-1	991	2,337	2,757	451	1,271	1,249	874	678	1,362	1,377	918	1,013	366	357	302	323	221	260	565	1,115	197	662	202	573
272	AACACACCCAGCTAACCTTTTT	rno-miR-329-3p	3,343	5,638	4,257	1,169	3,316	3,083	2,193	2,010	4,684	1,756	1,439	1,643	939	841	624	669	362	649	842	1,264	352	1,052	313	934
273	CCTCTGGGCCCTTCCTCCAGT	rno-miR-326-3p	972	1,844	2,265	387	1,158	1,048	658	468	1,243	1,637	999	1,111	365	348	313	351	231	277	634	780	164	420	176	719
274	CCTAGTAGGTGCTCAGTAAGT	rno-miR-325-5p_R-2	1,096	2,033	3,371	488	1,060	1,173	612	776	1,377	872	574	533	225	263	174	212	131	236	397	713	110	293	97	382
275	CGCATCCCCTAGGGCATTGGTGT	rno-miR-324-5p	342	636	681	139	394	396	232	155	390	410	305	309	134	127	104	129	78	120	288	296	70	171	47	187
276	ACTGCCCCAGGTGCTGCTGGT	rno-miR-324-3p_L-2R+1	91	193	233	31	118	87	81	67	138	161	69	80	19	30	23	28	7	22	73	79	18	28	18	171
277	AGGTGGTCCGTGGCGCGTTCG	rno-miR-323-5p_R-1	54	77	89	18	57	44	43	25	80	49	25	56	32	20	10	14	10	16	23	50	9	21	5	37
278	CACATTACACGGTCGACCTCT	rno-miR-323-3p	827	1,644	2,023	338	864	903	622	356	1,066	1,283	735	830	471	485	329	372	256	387	793	1,127	211	573	160	941
279	CAGCAGCAATTCATGTTTTGGA	rno-miR-322-5p	275	574	676	92	377	364	176	191	366	334	242	224	89	103	96	73	50	88	130	207	40	143	46	168
280	AAACATGAAGCGCTGCAACA	rno-miR-322-3p	737	1,854	2,331	470	1,064	1,020	684	462	995	1,222	1,004	771	268	253	224	203	133	255	489	688	147	365	110	567
281	AAAAGCTGGGTTGAGAGGGCGA	rno-miR-320-3p	898	2,290	2,517	467	951	866	617	564	953	1,354	858	831	254	273	209	150	147	220	792	781	92	428	78	308
282	TATTGCACATTACTAAGTTGC	rno-miR-32-5p_R-1	30	78	75	8	44	48	25	22	45	35	15	14	10	7	5	6	3	14	9	13	4	9	5	17
283	AGGCAAGATGCTGGCATAGCT	rno-miR-31a-5p_R-1	851	1,515	2,286	414	1,084	1,028	499	518	1,103	645	419	467	204	234	155	168	118	187	231	497	79	233	76	435
284	TGCTATGCCAACATATTGCCATC	rno-miR-31a-3p	23	65	64	12	21	24	23	20	50	20	18	12	8	8	4	4	6	7	12	13	3	6	2	0
285	CTTTCAGTCGGATGTTTACAGC	rno-miR-30e-3p	3,617	6,957	12,309	2,089	4,181	4,553	2,131	2,631	4,647	3,529	2,515	2,595	916	825	692	858	395	781	1,957	2,639	436	1,196	385	1,702
286	CTTTCAGTCAGATGTTTGCTGC	rno-miR-30d-3p	243	409	453	112	265	236	206	70	270	214	163	149	82	61	53	51	38	48	133	155	36	109	34	146
287	CTGGGAGAAGGCTGTTTACTCT	rno-miR-30c-2-3p	198	407	369	83	209	191	164	130	202	266	165	211	82	59	73	51	39	46	141	189	39	101	36	176
288	CTGGGAGAGGGTTGTTTACTCC	rno-miR-30c-1-3p	50	101	120	26	68	64	41	33	53	64	35	54	16	17	16	14	6	9	28	53	3	33	5	37
289	CTGGGATGTGGATGTTTACGTC	rno-miR-30b-3p	63	90	108	12	50	38	29	15	84	35	21	23	31	14	19	16	4	20	34	34	7	24	5	31
290	CTTTCAGTCGGATGTTTGCAGC	rno-miR-30a-3p	1,486	2,643	3,962	725	1,777	1,582	1,000	985	1,818	1,601	1,070	1,128	424	386	310	351	182	354	731	1,058	158	540	180	877
291	TAGGCTGGAAAGAGGTTGGGGA	rno-miR-3099_1ss7AG	271	643	603	97	286	284	193	152	387	431	201	245	85	103	65	60	17	63	294	206	38	114	23	125
292	TCTGGCTGCTATGGCCCCCTCT	rno-miR-3085_R+1	213	314	643	70	168	185	115	117	208	164	118	118	38	55	39	48	20	50	71	96	15	62	24	146
293	TGCCCCCTCCAGGAAGCCTTCT	rno-miR-3072_R-1	30	98	116	13	55	54	29	28	77	54	27	51	17	18	12	12	7	17	18	52	5	20	9	19
294	TTGGAGTTCATGCAAGTTCTAACC	rno-miR-3068-5p_R-1	286	735	893	171	388	388	233	178	378	311	276	269	224	184	132	161	142	161	296	451	88	283	101	373
295	GCTCTGACTTTATTGCACTACT	rno-miR-301a-5p_R+1	81	164	211	34	110	93	68	69	68	64	76	66	31	27	25	15	17	26	71	40	19	38	14	67
296	CAGTGCAATAGTATTGTCAAAGC	rno-miR-301a-3p	171	310	450	66	202	200	93	75	203	145	159	74	40	46	42	43	15	61	90	87	34	45	27	82
297	TGAAGAGAGGTTATCCTTTGTGT	rno-miR-300-5p_L-1R+2	29	39	70	6	17	30	8	12	35	39	29	10	5	3	4	7	2	0	10	15	3	0	2	29
298	TATGCAAGGGCAAGCTCTCTTC	rno-miR-300-3p	4,188	7,115	10,958	1,627	4,325	3,880	2,490	2,138	4,710	3,208	2,142	2,412	853	841	706	774	522	888	1,356	2,440	395	1,022	371	2,142
299	ACCGATTTCTCCTGGTGTTCAGA	rno-miR-29c-5p_L-2R+3	349	724	1,118	125	386	407	254	226	460	423	271	320	140	115	76	96	72	85	202	262	83	168	58	224
300	TAGCACCATTTGAAATCGGTT	rno-miR-29c-3p_R-1	2,278	3,333	4,650	741	2,249	2,390	1,187	855	2,495	1,797	1,370	1,126	555	581	483	645	276	625	856	1,126	262	650	226	1,282
301	TAGCACCATTTGAAATCAGTGTT	rno-miR-29b-3p	4,758	7,045	12,473	1,605	5,136	4,734	2,266	2,252	5,647	3,429	2,501	2,227	1,139	1,234	912	1,266	554	1,203	1,776	2,351	605	1,425	432	2,279
302	CTGGTTTCACATGGTGGCTTAGA	rno-miR-29b-2-5p_R+1	68	142	134	20	74	55	46	24	67	57	49	40	15	21	12	12	10	16	24	73	6	25	20	77
303	GCTGGTTTCATATGGTGGTTTAGA	rno-miR-29b-1-5p_R+2	27	58	87	18	52	31	16	23	33	20	13	22	4	5	6	6	2	5	5	21	2	5	0	11
304	ACTGATTTCTTTTGGTGTTCAGA	rno-miR-29a-5p_R+1	72	141	218	20	89	66	34	65	114	114	88	52	18	27	11	20	7	25	53	58	8	27	10	42
305	TATGTGGGACGGTAAACCGCTT	rno-miR-299b-3p_R+5	100	153	201	37	81	65	41	45	87	63	46	44	29	19	13	23	13	25	32	64	15	38	12	59
306	TGGTTTACCGTCCCACATACAT	rno-miR-299a-5p	37	77	45	13	33	27	21	21	50	20	2	3	7	10	7	5	0	5	3	12	2	2	0	6
307	GGCAGAGGAGGGCTGTTCTTCCC	rno-miR-298-5p	127	245	387	71	105	136	70	102	156	103	57	48	34	33	14	11	4	27	63	40	6	31	8	26
308	GAGGAACTAGCCTTCTCTCTGCTT	rno-miR-298-3p_L+1R+1	40	76	101	14	32	31	20	8	32	51	31	19	13	18	5	4	0	6	36	19	4	21	4	37

309	AGGGCCCCCCCTCAATCCTGT	rno-miR-296-5p	22	41	31	6	8	14	10	5	18	22	15	16	7	5	8	4	2	2	16	5	0	2	5	6
310	ACTCAAACTGTGTGACACTTT	rno-miR-293-5p	0	13	0	0	0	4	0	0	13	3	0	4	2	0	0	4	4	0	2	7	0	8	0	0
311	AAGGAGCTCACAGTCTATTGA	rno-miR-28-5p_R-1	269	588	820	120	326	316	188	165	378	250	193	188	55	74	54	47	28	59	163	166	28	85	36	121
312	CACTAGATTGTGAGCTCCTGGA	rno-miR-28-3p	242	418	634	108	235	241	166	147	261	325	238	196	66	69	59	45	32	50	154	130	22	110	35	140
313	AGAGCTTAGCTGATTGGTGAAC	rno-miR-27b-5p_R-2	46	97	116	22	51	66	33	30	54	62	53	35	26	11	16	14	8	16	24	37	6	15	8	34
314	AGGGCTTAGCTGCTTGTGAGCA	rno-miR-27a-5p	14	35	23	4	13	16	5	7	11	18	11	8	4	2	0	2	4	2	5	10	0	5	2	10
315	CCTGTTCTCCATTACTTGGCTC	rno-miR-26b-3p	33	60	101	13	32	33	13	15	39	26	26	34	3	8	10	8	5	3	13	13	0	5	2	51
316	AGGCGGAGACACGGGCAATTGCT	rno-miR-25-5p_R+1	17	49	58	15	25	18	19	15	41	17	16	13	8	9	3	4	2	3	11	21	2	5	0	7
317	CATTGCACTTGTCTCGGTCTGA	rno-miR-25-3p	832	1,665	1,773	297	836	781	557	515	1,059	709	564	444	270	307	167	193	96	237	637	544	81	237	102	555
318	GTGCCTACTGAGCTGAAACAGT	rno-miR-24-2-5p	3,043	7,787	10,002	1,909	4,625	4,231	1,800	1,677	4,118	2,558	1,969	2,017	972	728	746	674	449	649	1,037	1,871	530	1,094	448	1,482
319	GTGCCTACTGAGCTGATATCAGT	rno-miR-24-1-5p_R+1	252	545	649	137	282	294	140	148	338	222	142	157	74	60	68	64	32	60	91	145	27	70	31	125
320	GGGTTCCTGGCATGCTGATTT	rno-miR-23b-5p	45	80	91	13	39	46	26	23	47	47	21	32	7	9	15	2	0	6	14	19	0	16	0	27
321	ATCACATTGCCAGGGATTACCACT	rno-miR-23b-3p_R+3	4,440	10,873	9,930	2,287	5,872	5,775	3,621	2,551	6,331	5,216	3,901	4,557	2,169	1,933	1,783	1,836	1,450	1,524	3,618	5,377	1,142	3,263	990	3,971
322	ATCACATTGCCAGGGATTTCC	rno-miR-23a-3p	2,632	8,107	7,708	1,937	4,296	4,114	2,382	1,549	4,009	4,147	3,152	3,572	1,669	1,293	1,226	1,316	1,185	1,018	2,763	4,451	982	2,579	770	2,206
323	CAAGTCACTAGTGGTTCCGTTT	rno-miR-224-5p	33	79	130	14	50	88	16	2	29	78	62	26	20	4	10	11	6	6	54	22	3	21	0	22
324	TGTCAGTTTGTCAAATACCCCA	rno-miR-223-3p_R+1	34	93	144	6	72	46	22	21	62	72	41	22	10	7	18	6	8	5	42	22	11	25	7	27
325	GGCTCAGTAGCCAGTGTAGATCC	rno-miR-222-5p_R+2	20	45	60	8	26	27	16	11	17	32	23	13	3	4	3	7	4	6	13	13	3	8	2	17
326	AGTTCTTCAGTGGCAAGCTTT	rno-miR-22-5p_R-1	361	579	789	116	369	345	260	222	453	373	212	204	87	100	77	95	31	102	146	197	28	102	41	206
327	TGATTGTCCAAACGCAATTCT	rno-miR-219a-5p	291	583	658	68	303	341	272	153	295	323	231	280	157	191	102	115	64	165	180	251	53	126	57	407
328	AGAATTGTGGCTGGACATCTGT	rno-miR-219a-2-3p	1,265	2,360	5,710	492	1,229	1,282	982	1,356	1,848	880	481	726	419	577	281	303	111	522	554	1,000	134	349	143	494
329	TTGTGCTTGATCTAACCATGT	rno-miR-218a-5p	3,437	5,645	9,875	1,439	4,061	4,104	2,143	1,947	4,911	3,016	2,448	2,600	979	1,113	794	1,087	514	1,135	1,132	2,161	457	1,113	385	2,155
330	ATGGTTCTGTCAAGCACCGCGT	rno-miR-218a-2-3p_L-1R+1	12	33	42	0	19	12	4	9	14	21	17	17	13	8	5	3	2	0	9	11	2	7	0	27
331	TAATCTCAGCTGGCAACTGTGA	rno-miR-216a-5p	22	51	28	15	26	20	8	9	20	36	27	22	8	6	7	6	2	8	35	27	2	14	3	14
332	ACAGCAGGCACAGACAGGCAGT	rno-miR-214-3p_R+1	15	27	38	4	17	24	12	4	20	42	30	11	6	0	2	0	2	0	42	10	3	6	5	19
333	TAACAGTCTCCAGTCACGGCC	rno-miR-212-3p_R-1	1,514	2,809	3,130	811	1,779	1,903	1,320	809	2,253	1,870	1,480	1,445	617	659	453	544	322	552	935	1,531	255	691	212	1,147
334	TTCCCTTTGTCATCCTTTGCCT	rno-miR-211-5p	103	135	190	34	59	85	37	23	76	143	70	78	21	43	11	4	12	37	82	44	10	21	2	29
335	AGCCACTGCCCACAGCACACTG	rno-miR-210-5p	6	11	20	3	8	4	8	2	9	14	7	3	3	0	0	0	0	5	0	4	0	2	0	13
336	CTGTGCGTGTGACAGCGGCTGA	rno-miR-210-3p	94	209	255	33	102	116	83	26	98	189	115	113	28	40	24	47	21	28	68	67	10	50	25	150
337	TAGCTTATCAGACTGATGTTGA	rno-miR-21-5p	3,676	9,021	14,241	1,587	4,233	4,673	3,001	3,351	5,763	4,304	2,902	3,122	1,288	1,549	912	1,099	654	1,334	3,196	3,183	584	1,488	654	1,987
338	CAACAGCAGTCGATGGGCTGT	rno-miR-21-3p_R-1	16	18	35	2	16	10	15	8	25	10	12	13	2	5	0	0	3	7	8	7	0	2	0	7
339	TAAAGTGCTTATAGTGCAGGTAG	rno-miR-20a-5p	233	631	859	118	300	354	203	201	347	413	297	279	103	130	87	91	51	101	308	281	52	169	53	262
340	TCCTTCATTCCACCGGAGTCTGA	rno-miR-205_1ss23TA	19	25	27	4	14	21	10	0	25	23	16	11	11	8	0	5	2	11	6	17	2	10	4	17
341	GTGAAATGTTTAGGACCACTAGA	rno-miR-203a-3p_R+1	201	529	632	100	293	289	137	105	272	359	292	134	47	58	50	35	31	62	284	139	30	98	22	124
342	CACTCAGTAAGGCATTGTTCTT	rno-miR-201-5p_R+2	21	36	12	8	16	15	7	6	17	17	9	5	5	3	3	4	2	2	6	6	2	5	0	8
343	TAATACTGCCGGGTAATGATGGA	rno-miR-200c-3p_R+2	37	36	37	3	11	24	3	2	157	769	608	7	6	16	9	11	6	74	2,261	17	3	8	0	4
344	CATCTTACTGGGCAGCATTGGA	rno-miR-200b-5p	13	7	8	4	0	2	0	0	32	143	135	3	0	2	0	0	0	6	421	0	0	0	0	2
345	CATCTTACCGGACAGTGCTGGA	rno-miR-200a-5p_R+1	2	0	2	0	0	0	0	0	18	86	77	0	0	0	0	0	0	4	173	4	0	0	0	0
346	TGTGCAAATCCATGCAAAACT	rno-miR-19b-3p_R-2	74	152	209	27	70	88	47	45	99	100	106	66	32	43	34	24	25	30	89	83	24	70	19	65
347	TGTGCAAATCTATGCAAAACTGA	rno-miR-19a-3p	15	32	65	0	9	20	12	10	25	22	17	11	8	5	2	5	5	4	24	20	8	14	7	12
348	CCCAGTGTTCAGACTACCTGTTC	rno-miR-199a-5p	1,369	5,766	4,467	1,262	2,477	2,976	1,715	468	1,994	4,474	3,251	2,658	977	600	656	583	549	392	3,848	1,838	412	1,709	641	2,305
349	ACAGTAGTCTGCACATTGGTT	rno-miR-199a-3p_R-1	965	2,204	3,436	537	1,437	1,317	683	429	1,617	1,473	815	714	288	262	215	296	133	215	1,283	512	142	371	147	655
350	TAGCAGCACAGAAATATTGGC	rno-miR-195-5p	1,201	3,281	2,875	618	1,628	1,839	983	717	1,666	1,617	1,354	1,334	608	567	493	414	334	384	1,252	1,126	241	702	243	1,043
351	CCAATATTGGCTGTGCTGCTCCA	rno-miR-195-3p	32	44	51	13	14	20	12	12	20	15	22	15	2	5	8	0	0	2	12	9	6	9	3	15
352	TATACCAGGATGTCAGCATAGTT	rno-miR-1949	18	21	31	18	22	57	37	6	22	31	28	45	15	17	16	22	14	17	40	31	5	37	11	26
353	TGTAACAGCAACTCCATGTGGA	rno-miR-194-5p	67	152	222	33	94	71	40	39	88	83	55	40	27	35	24	31	11	33	57	70	10	34	8	69
354	CTGACCTATGAATTGACAGCC	rno-miR-192-5p	3,645	7,253	11,906	2,172	4,074	4,140	2,265	3,331	5,720	2,366	1,945	1,756	745	941	634	688	404	1,028	1,564	1,888	398	847	294	1,374
355	CTGCACTTGGATTTCGTTCCC	rno-miR-191a-3p_L-1	145	243	318	68	162	155	102	66	171	156	95	108	60	51	36	35	26	39	76	93	21	70	17	172
356	TGATATGTTTGATATTAGGTTG	rno-miR-190b-5p_R+1	126	238	386	74	156	115	73	118	162	95	60	63	33	27	22	31	15	34	38	61	10	35	3	46
357	TGATATGTTTGATATATTAGGTT	rno-miR-190a-5p_R+1	294	605	893	155	362	384	202	162	427	314	288	218	74	97	82	75	47	99	117	163	51	120	50	252
358	CTATATATCAAGCATATTCCT	rno-miR-190a-3p_L-1	44	135	124	19	52	73	43	21	56	47	64	49	16	16	19	28	14	21	31	43	11	40	9	44
359	TAAGGTGCATCTAGTGCAGATAGA	rno-miR-18a-5p_R+1	13	25	35	4	12	14	0	4	15	13	7	4	2	2	4	0	0	0	12	5	2	3	0	7
360	CTCCCACATGCAGGGTTTGCA	rno-miR-188-3p_R+1	6	14	22	3	9	11	5	5	10	12	12	7	0	0	0	0	2	2	3	8	4	5	4	3

361	TCGTGTCTTGTGTTGCAGCCGGA	rno-miR-187-3p_R+1	91	316	406	31	100	107	59	56	103	214	126	88	22	38	17	20	14	33	147	75	5	37	6	117
362	TGGAGAGAAAGGCAGTTCCTGA	rno-miR-185-5p	4,190	8,887	10,849	2,388	5,096	5,250	3,022	2,115	4,369	5,581	3,855	4,534	1,933	1,828	1,604	1,433	1,274	1,539	3,588	5,746	950	3,378	963	3,862
363	AGGGGCTGGCTTTCCTCTGGT	rno-miR-185-3p	63	137	211	48	81	77	44	39	95	69	53	65	27	13	11	14	12	20	33	77	6	29	6	53
364	TATGGAGGTCTCTGTCTGACT	rno-miR-1843-5p	2,012	3,808	3,767	821	1,865	1,910	1,350	1,011	1,962	1,953	1,473	1,404	577	513	426	376	232	404	941	1,259	83	599	206	1,169
365	TCTGATCGTTCACCTCCATAC	rno-miR-1843-3p_R-1	192	464	519	102	231	194	138	146	293	249	143	131	68	44	42	43	24	49	112	147	7	43	33	153
366	TGGACGGAGAACTGATAAGGGT	rno-miR-184	61	77	78	27	46	50	17	16	61	33	31	33	6	8	11	16	6	13	18	25	2	10	0	7
367	AAGGTAGATAGAACAGGTCTTGT	rno-miR-1839-5p_R+1	1,724	3,084	4,821	1,017	1,879	1,869	1,002	1,055	2,115	1,733	1,185	1,171	443	411	299	306	222	419	867	1,187	86	580	161	599
368	AGACCTACTTATCTACCAACA	rno-miR-1839-3p_R-1	127	311	380	78	162	195	83	97	217	146	103	79	40	33	20	30	27	31	76	107	17	67	15	45
369	TGAATTACCGAAGGGCCATAA	rno-miR-183-3p	15	15	18	2	0	6	2	0	54	104	218	6	0	0	0	0	0	7	489	2	0	2	0	0
370	AACATTCATTGTTGTCGGTGGGT	rno-miR-181d-5p	2,032	3,891	4,827	897	2,236	2,187	1,400	1,199	2,373	2,205	1,565	1,522	542	567	496	457	300	476	1,116	1,574	283	810	248	1,272
371	AACATTCAACCTGTCGGTGAGT	rno-miR-181c-5p	4,112	7,335	8,780	1,462	3,993	4,056	2,322	1,820	5,086	3,009	2,634	1,877	869	721	673	702	416	809	1,378	1,715	421	933	339	1,501
372	ACCATCGACCGTTGAGTGGACC	rno-miR-181c-3p	431	788	615	165	343	405	316	117	365	416	408	333	164	137	117	107	67	95	268	335	54	200	68	328
373	AACATTCATTGCTGTCGGTGGGT	rno-miR-181b-5p	2,945	5,905	10,029	1,331	3,285	3,337	2,269	2,287	4,290	3,243	2,353	2,204	886	1,090	679	839	499	982	2,186	2,503	384	1,080	346	1,710
374	CTCACTGAACAATGAATGCAT	rno-miR-181b-1-3p_1ss21AT	55	137	185	23	62	74	31	43	68	37	34	26	8	8	0	8	11	10	13	30	4	13	6	23
375	ACCACCAACCGTTGACTGTACC	rno-miR-181a-2-3p_R+3	45	85	139	18	39	55	31	35	54	56	38	32	21	16	14	12	4	12	42	27	0	18	10	34
376	ACCATCGACCGTTGATTGTACC	rno-miR-181a-1-3p	1,208	2,300	3,056	484	1,153	1,311	900	790	1,445	1,090	851	722	414	387	255	296	212	328	768	853	176	482	164	826
377	CAAAGTGCTTACAGTGCAGGTAG	rno-miR-17-5p	76	173	248	32	95	105	65	47	96	124	81	77	29	43	22	30	22	25	71	86	17	48	19	95
378	ACTGCAGTGAAGGCACTTGTGGA	rno-miR-17-1-3p_R+1	40	86	107	10	37	43	25	10	39	25	20	23	21	9	6	10	6	10	21	30	4	17	7	25
379	TAGCAGCACATCATGGTTTACA	rno-miR-15b-5p	230	490	1,058	107	316	303	155	204	410	289	193	181	94	88	65	107	52	80	171	184	58	105	40	183
380	CGAATCATTATTTGCTGCTCT	rno-miR-15b-3p_R-1	12	24	48	2	13	21	4	11	15	19	14	7	4	4	2	2	2	6	15	7	2	6	0	6
381	TAGGTTATCCGTGTTGCCTTCG	rno-miR-154-5p	68	191	229	36	114	96	69	56	151	86	73	97	37	31	27	33	18	31	34	80	14	49	16	74
382	AATCATACACGGTTGACCTATT	rno-miR-154-3p	12	17	42	3	11	12	10	8	22	16	8	11	4	7	3	3	2	5	7	7	0	3	0	4
383	GTCATTTTTGTGATGTTGCAGCT	rno-miR-153-5p	301	656	552	129	390	394	241	133	299	391	354	301	145	102	127	93	74	85	150	231	63	226	68	271
384	AGGTTCTGTGATACACTCCGACT	rno-miR-152-5p	6	14	10	2	10	13	5	0	5	18	14	10	4	3	2	3	2	0	17	10	0	7	0	8
385	TCAGTGCATGACAGAACTTGG	rno-miR-152-3p	943	2,377	3,180	515	1,445	1,345	695	534	1,246	1,917	1,202	1,030	403	377	277	291	244	307	1,090	983	210	506	179	839
386	CTAGACTGAGGCTCCTTGAGG	rno-miR-151-3p	6,323	11,721	17,738	3,351	6,446	6,654	3,894	4,007	6,988	5,129	3,643	3,668	1,600	1,409	1,165	1,113	709	1,318	2,787	3,770	662	1,786	548	2,362
387	TCTCCCAACCCTTGTACCAGTG	rno-miR-150-5p	5,187	8,639	11,003	2,466	5,225	5,054	3,505	2,735	6,545	4,933	3,893	4,063	1,637	1,278	1,194	1,186	935	1,108	1,833	3,291	632	1,781	987	3,623
388	GAAGTTCTGTTATACACTCAGGC	rno-miR-148b-5p_R+1	137	259	429	69	131	156	86	65	164	131	124	107	43	32	41	26	22	34	94	111	16	56	16	65
389	TCAGTGCATCACAGAACTTTGT	rno-miR-148b-3p	5,000	10,539	12,385	2,542	5,897	6,025	3,294	2,917	6,760	4,562	3,741	3,947	1,602	1,493	1,254	1,254	968	1,507	2,688	3,929	873	2,154	707	3,285
390	GCCCTAGGGACTCAGTTCTGGT	rno-miR-146b-3p_L+2R-1	98	178	210	39	110	92	70	49	91	90	57	65	38	28	25	26	13	14	32	61	13	44	13	86
391	TGAGAACTGAATTCCATGGGTT	rno-miR-146a-5p	2,716	7,631	6,921	2,011	4,593	4,226	2,760	1,695	3,249	4,911	4,384	3,969	1,405	1,205	1,151	713	606	911	2,827	3,008	435	1,898	407	1,662
392	ACCTGTGAAGTTCAGTTCTTTA	rno-miR-146a-3p_R+1	28	50	86	16	26	47	25	16	47	27	18	15	11	7	12	9	4	4	23	17	0	8	2	20
393	GTCCAGTTTTCCCAGGAATCCCT	rno-miR-145-5p	1,617	5,612	4,132	842	2,607	2,421	2,071	922	2,154	4,348	3,484	2,774	1,064	983	907	711	933	552	1,998	2,302	736	2,111	956	1,847
394	GGATTCCTGGAAATACTGTTCT	rno-miR-145-3p_R+1	1,739	4,315	3,764	1,028	2,422	2,087	1,661	830	1,903	2,445	2,162	1,758	685	543	529	425	329	448	1,121	1,384	245	812	294	1,327
395	GGATATCATCATATACTGTAAGT	rno-miR-144-5p	20	45	41	8	33	17	23	8	22	51	15	21	12	6	0	3	6	4	14	14	2	14	8	26
396	TACAGTATAGATGATGTACT	rno-miR-144-3p	165	353	231	79	407	197	158	64	222	491	269	332	100	85	30	89	41	49	182	141	22	110	43	550
397	GGTGCAGTGCTGCATCTCTGGTA	rno-miR-143-5p_R+2	266	635	752	104	396	342	176	110	271	455	281	277	131	114	82	89	60	80	179	230	43	138	63	190
398	CCCATAAAGTAGAAAGCACTAC	rno-miR-142-5p_L+2R-1	965	2,493	2,778	564	1,486	1,391	717	627	1,416	1,162	1,080	939	416	350	247	277	197	331	665	717	208	524	159	744
399	TGTAGTGTTTCCTACTTTATGG	rno-miR-142-3p_R-1	85	182	240	36	102	111	52	48	99	108	94	67	32	28	24	22	16	28	82	64	19	57	18	65
400	CATCTTCCAGTGCAGTGTTGGAC	rno-miR-141-5p_L-2R+3	0	0	0	0	0	0	0	0	3	4	13	3	0	0	0	0	0	0	33	0	0	0	0	0
401	TAACACTGTCTGGTAAAGATG	rno-miR-141-3p_R-1	66	85	90	20	31	48	7	4	393	1,223	1,522	22	2	23	12	26	7	117	3,107	20	2	2	0	10
402	CAGTGGTTTTACCCTATGGTAG	rno-miR-140-5p	191	402	521	76	242	257	132	125	268	280	160	176	78	90	73	70	56	70	197	162	50	147	40	218
403	ACCACAGGGTAGAACCACGGAC	rno-miR-140-3p_L-1R+2	1,304	3,152	2,398	596	1,345	1,427	1,067	560	1,486	2,345	1,672	1,348	623	574	475	393	285	473	1,412	1,269	175	745	241	1,018
404	TCTACAGTGCACGTGTCTCCAGT	rno-miR-139-5p_R+1	7,758	12,399	14,719	2,867	7,564	7,658	4,703	3,467	8,153	7,026	4,268	5,009	2,484	1,895	2,068	2,171	1,187	1,861	2,856	4,907	1,021	3,121	1,108	5,347
405	TGGAGACGCGGCCCTGTTGGAGT	rno-miR-139-3p_R+1	347	645	760	203	372	392	219	141	346	355	225	296	113	108	107	74	52	117	178	269	42	165	32	137
406	AGCTGGTGTTGTGAATCAGGCCG	rno-miR-138-5p	4,409	8,417	8,358	1,464	4,586	4,504	3,063	1,850	4,776	5,196	3,634	3,770	1,890	1,624	1,340	1,322	836	1,326	2,405	3,127	765	2,202	792	5,329
407	GCTATTTCACGACACCAGGGT	rno-miR-138-2-3p	41	86	78	19	52	49	43	25	48	62	41	38	17	15	10	18	9	16	46	51	11	33	2	41
408	GCTACTTCACAACACCAGGGT	rno-miR-138-1-3p_L-2R+1	131	240	395	59	177	144	94	72	162	136	127	117	39	59	35	54	26	51	95	110	17	53	9	152
409	ACGGGTATTCTTGGGTGGATAAT	rno-miR-137-5p_R+1	2	13	19	2	6	7	4	4	17	7	4	7	0	0	2	0	0	0	0	0	2	0	0	16
410	TTATTGCTTAAGAATACGCGT	rno-miR-137-3p_R-2	3,889	7,098	11,236	1,168	4,028	4,721	2,407	1,886	4,792	4,511	2,884	3,110	1,157	1,202	915	1,108	710	1,196	1,731	2,294	647	1,541	635	1,792
411	ACTCCATTTGTTTTGATGATGG	rno-miR-136-5p_R-1	3,962	6,821	9,575	2,142	5,039	4,585	2,728	2,335	4,743	3,835	2,863	2,819	1,224	1,035	897	961	596	1,026	1,620	2,602	462	1,358	410	2,299
412	TATGGCTTTTCATTCCTATGTGA	rno-miR-135b-5p	388	916	891	172	474	479	286	176	558	545	431	307	132	200	115	145	80	130	463	309	77	221	89	348

413	TATGGCTTTTTATTCCTATGTGA	rno-miR-135a-5p	2,050	4,072	7,328	963	2,557	2,730	1,354	1,347	2,889	2,936	2,077	1,542	784	980	605	732	447	760	1,458	1,698	517	1,397	478	1,572
414	TGTAGGGATGGAAGCCATGAAA	rno-miR-135a-3p	83	136	162	42	70	68	42	28	73	104	60	56	20	19	10	21	5	20	39	54	2	25	5	40
415	TGTGACTGGTTGACCAGAGGGG	rno-miR-134-5p	757	1,245	1,906	294	757	698	441	399	917	756	431	483	182	238	154	186	111	239	387	558	95	260	73	531
416	CTGTGGGCCACCTAGTCACC	rno-miR-134-3p_R-2	86	142	155	33	81	59	44	46	130	30	25	33	11	21	14	15	12	15	24	48	4	22	6	39
417	TTTGGTCCCCTTCAACCAGCT	rno-miR-133b-3p_R-1	21	46	34	5	29	22	11	7	24	29	25	17	10	8	7	8	5	6	10	10	3	11	5	12
418	TTGGTCCCCTTCAACCAGCTGT	rno-miR-133a-3p_L-1R+1	321	691	508	149	489	420	237	148	442	399	260	240	126	74	128	129	57	75	119	267	45	141	57	367
419	ACTCTTTCCCTGTTGCACTACT	rno-miR-130b-5p	36	62	89	20	44	43	20	23	43	64	41	30	10	15	9	3	6	10	50	26	11	14	8	41
420	CAGTGCAATGTTAAAAGGGCAT	rno-miR-130a-3p	530	1,013	1,296	173	682	693	417	247	602	753	526	382	174	159	149	144	92	153	397	374	93	225	90	465
421	CCACCTCCCCTGCAAACGTCC	rno-miR-1306-5p_R-1	10	14	21	4	6	10	11	3	17	12	9	12	3	2	0	0	0	2	7	2	0	3	2	20
422	CATCTGGGCAACTGATTGAACT	rno-miR-1298-3p	58	120	320	25	62	43	35	67	48	74	39	32	19	32	21	9	11	35	15	37	6	10	0	50
423	TTCATTCGGCTGTCCAGATGTA	rno-miR-1298	124	192	1,221	56	139	119	63	183	143	106	37	37	30	46	19	18	10	67	33	75	18	42	9	38
424	CTTTTTGCGGTCTGGGCTTGC	rno-miR-129-5p	2,339	4,792	5,845	1,031	2,391	2,348	1,718	1,670	3,465	2,749	1,752	1,994	720	723	536	604	348	625	1,432	2,142	353	973	389	1,977
425	GGGGGCCGATGCACTGTAAGAGA	rno-miR-128-2-5p_R+2	616	1,586	1,931	348	690	746	406	434	748	427	331	477	166	155	148	120	79	146	244	465	70	253	49	101
426	CGGGGCCGTAGCACTGTCTGA	rno-miR-128-1-5p	143	337	362	63	159	138	109	60	174	125	85	120	26	27	31	27	24	40	57	128	14	47	12	64
427	CTGAAGCTCAGAGGGCTCTGATT	rno-miR-127-5p	873	1,921	2,258	376	991	1,013	791	506	1,133	1,635	1,029	1,064	361	366	278	353	202	276	711	1,121	153	531	187	1,241
428	ACAAGTCAGGCTCTTGGGACCT	rno-miR-125b-2-3p	4,035	9,488	8,551	2,421	4,407	4,457	3,384	2,191	4,240	4,788	3,437	3,706	1,445	1,353	1,121	911	885	999	2,269	3,642	693	2,120	629	2,293
429	ACGGGTTAGGCTCTTGGGAGT	rno-miR-125b-1-3p_R-1_1ss21CT	1,040	1,830	3,024	509	1,257	1,101	637	540	1,165	1,145	714	740	275	312	212	220	144	301	510	662	132	345	89	432
430	ACAGGTGAGGTTCTTGGGAGC	rno-miR-125a-3p_R-1	42	107	121	21	63	59	42	27	79	82	44	59	19	27	22	13	11	19	43	70	8	35	11	45
431	ACGCCCTTCCCCCCCTTCTTCA	rno-miR-1249	862	1,546	2,265	366	994	738	529	551	1,223	1,078	591	632	175	206	156	182	101	195	392	594	92	292	139	310
432	CGTGTTCACAGCGGACCTTGAT	rno-miR-124-5p	148	336	383	51	171	187	123	68	216	205	136	121	45	75	54	63	33	46	196	140	28	68	14	168
433	GTGAGGACTGGGGAGGTGGAGGGT	rno-miR-1224_R+3	473	791	883	187	447	404	258	198	421	432	258	304	115	105	108	97	36	102	197	323	40	159	27	65
434	CCCCACCTCTTCTCTCCTCAG	rno-miR-1224-3p	3	8	4	0	2	3	4	2	11	0	0	2	0	0	0	0	0	0	0	2	3	0	0	10
435	TAGGTCACCCGTTTTACTATCC	rno-miR-1193-3p	506	1,050	1,730	300	697	701	361	415	768	351	251	310	163	158	146	119	75	133	151	310	104	228	64	347
436	TGGTGTGAGGTTGGGCCAGGA	rno-miR-1188-5p	11	30	20	4	12	16	0	3	8	9	7	16	2	0	2	0	0	0	5	5	2	5	0	6
437	TACCCTGTAGAACCGAATTTGT	rno-miR-10b-5p_L+2R-2	349	1,370	2,432	352	824	769	434	252	1,332	765	512	421	152	107	164	52	86	113	356	297	119	202	92	112
438	TACCCTGTAGATCCGAATTTGT	rno-miR-10a-5p_R-1	499	1,843	2,954	170	2,755	2,309	1,198	506	4,390	595	1,471	529	101	130	81	31	128	149	406	419	82	493	35	81
439	AGCAGCATTGTACAGGGCTATC	rno-miR-107-3p_R-1	1,875	3,548	3,990	611	1,751	1,978	1,060	918	2,142	1,656	1,100	1,099	522	548	407	459	266	455	989	1,148	231	670	238	1,074
440	TAAAGTGCTGACAGTGCAGATA	rno-miR-106b-5p_R+1	249	506	933	85	281	250	120	165	391	271	145	175	65	70	56	90	19	80	212	128	28	63	28	175
441	CCGCACTGTGGGTACTTGCTGC	rno-miR-106b-3p	153	298	464	63	149	145	82	78	194	169	94	84	33	45	32	27	19	37	122	96	14	38	10	94
442	AGCAGCATTGTACAGGGCTATGA	rno-miR-103-3p	7,536	15,103	16,967	3,203	7,118	7,590	4,477	4,024	7,728	7,858	4,820	5,092	2,214	2,077	1,667	1,846	1,137	1,976	4,351	6,007	1,011	3,563	1,014	3,950
443	AGCTTCTTTACAGTGCTGCCTTGT	rno-miR-103-2-5p_R+7	15	26	44	7	22	18	16	3	28	30	18	18	0	6	4	2	0	4	14	14	3	8	6	3
444	TCAGTTATCACAGTGCTGATGC	rno-miR-101a-5p	38	73	95	8	36	32	20	21	58	48	29	23	9	7	5	8	10	10	14	16	4	10	4	19
445	CTGCGCAAGCTACTGCCTTGCT	rno-let-7i-3p	134	300	249	30	158	130	99	61	194	170	91	105	60	52	37	34	15	43	91	108	17	63	23	196
446	CTATACAGTCTACTGTCTTTCT	rno-let-7f-2-3p_R+1	114	178	188	39	104	114	86	46	125	139	126	112	49	40	41	28	25	40	100	97	18	74	16	90
447	CTATACAATCTATTGCCTTCCT	rno-let-7f-1-3p_R+1	70	180	225	45	106	96	62	41	108	102	64	58	33	22	21	25	11	21	37	60	14	36	15	60
448	CTATACGGCCTCCTAGCTTTCC	rno-let-7e-3p	176	338	471	69	184	177	121	117	226	163	119	126	57	46	39	54	25	42	85	105	23	76	25	150
449	CTATACGACCTGCTGCCTTTCT	rno-let-7d-3p	1,448	3,085	3,128	565	1,620	1,302	884	819	1,776	1,963	956	1,255	567	468	376	416	244	366	1,062	1,034	200	674	328	1,336
450	CTGTACAACCTTCTAGCTTTCC	rno-let-7c-1-3p	46	110	205	23	76	61	35	43	75	92	49	42	17	14	8	12	13	13	25	43	9	29	9	33
451	CTATACAACCTACTGCCTTCCT	rno-let-7b-3p_1ss22CT	614	1,343	1,809	326	713	731	381	388	822	625	379	475	211	145	159	126	111	143	337	490	101	242	108	319
452	CTATACAATCTACTGTCTTTCT	rno-let-7a-1-3p_1ss22CT	951	2,265	2,742	494	1,234	1,186	797	758	1,401	1,410	1,232	951	376	434	292	312	231	343	1,080	1,170	230	672	199	664
453	CTATACAATCTACTGTCTTTCC	rno-let-7a-1-3p	261	620	878	139	329	343	194	251	404	354	311	221	78	112	72	82	50	82	266	299	54	151	45	144
454	ATAAAGCTAGATAACCGAAGA	rno-mir-9b-3-p3_2ss4CA17AG	0	1	1	0	1	2	0	0	0	0	0	0	0	0	0	0	0	0	2	0	0	1	0	0
455	TCTGAGCAATCATGTGCAGTGCA	rno-mir-96-p3_1ss23CA	0	0	0	0	0	0	0	0	0	0	0	0	0	0	0	0	0	0	2	0	0	0	0	0
456	GCCCCTCGGCCATCCTCCGTCT	rno-mir-935-p5	0	0	2	0	0	0	0	0	0	4	0	0	0	0	0	0	0	0	0	0	0	0	0	0
457	CTGGGTAGAGCAGGGCT	rno-mir-878-p3_1ss11GC	0	0	0	0	0	0	0	0	0	2	0	0	0	0	0	0	0	0	2	0	0	0	0	0
458	GTGGTTTGCAAAGTAATTCATA	rno-mir-876-p3	0	0	0	0	0	2	0	0	0	0	2	2	0	0	0	0	0	0	0	0	0	0	0	0
459	TCAGCTGAAAACACT	rno-mir-875-p3_1ss4CG	0	0	0	0	0	0	0	0	2	0	0	0	0	0	0	0	0	2	0	0	0	0	0	0
460	ACGTGAAATGTAAGA	rno-mir-742-p5_1ss15TA	2	0	0	0	0	0	0	0	0	0	0	0	0	0	0	0	0	0	0	0	0	0	0	0
461	TATGTTGGGTAAAGTA	rno-mir-742-p3_1ss1CT	0	2	0	0	0	0	0	0	0	0	0	0	0	0	0	0	0	0	0	0	0	0	0	0
462	CCTGACTTTGAATCTC	rno-mir-711-p5_1ss4AG	0	0	2	0	0	0	0	0	0	0	0	0	0	0	0	0	0	0	0	0	0	0	0	0
463	CGAAAGCTGGGGACC	rno-mir-711-p3_1ss1GC	0	0	4	0	0	0	0	0	0	0	0	0	0	0	0	0	0	0	0	0	0	0	0	0
464	TGCCTCGGGTGAGCATGCACTTAAC	rno-mir-668-p5	0	0	2	0	2	0	0	2	0	0	0	0	0	0	2	0	0	0	0	0	0	0	0	0
465	AGTTCACTGGGTTTT	rno-mir-653-p3_1ss12GT	0	0	0	0	4	0	0	0	0	0	0	0	0	0	0	0	0	0	0	0	0	0	0	0
466	CAGGCTATGGCTGCT	rno-mir-6333-p5_1ss5TC	0	0	0	0	0	0	0	0	0	0	0	0	0	0	0	2	0	0	0	0	0	0	0	4

467	TAGGCTGGCCTTGAACTG	rno-mir-6333-p3_2ss9AC17AT	0	0	2	0	0	0	2	0	0	4	0	0	0	0	0	0	0	0	0	0	0	0	0	2
468	AGGGCCCAGAGAAGC	rno-mir-6328-p5_1ss12CA	0	0	5	0	3	2	0	0	2	0	0	4	0	0	0	0	0	0	4	2	0	0	0	0
469	AGGCTGAGGAGTCTGTCCT	rno-mir-6327-p3_1ss17TC	0	0	0	0	0	0	2	0	0	0	0	2	0	0	0	0	0	0	0	0	0	0	0	2
470	CTGCCTTTTGAGTGC	rno-mir-6326-p5_1ss6CT	2	0	0	2	2	2	2	2	2	2	0	0	0	0	0	2	0	0	2	0	0	0	0	0
471	AAGAGTCTCCGTCTGGCCTTTCA	rno-mir-6325-p5	0	0	4	0	0	4	0	0	0	0	0	0	0	0	0	0	0	0	0	0	0	0	0	0
472	TGCCCCGAGCAGGAT	rno-mir-6323-p5_1ss6AC	0	0	4	0	0	0	0	0	0	0	0	2	0	0	0	0	0	0	0	0	0	0	0	0
473	TCTCTGTGTTCCTCCT	rno-mir-6322-p3_1ss8CG	0	0	0	0	0	0	0	0	0	0	0	0	0	0	0	0	0	0	0	0	0	0	0	2
474	CAGTGCTCGTGGGTCTC	rno-mir-6321-p5_1ss9AG	0	0	2	0	0	0	0	0	0	0	2	0	0	0	0	0	0	0	0	0	0	0	0	0
475	GAAGCCAATGCTCTCATGA	rno-mir-6321-p3	0	0	0	0	0	2	0	0	0	0	0	0	0	0	0	0	0	0	0	2	0	0	0	0
476	GGAGGTTGAGATAGAACT	rno-mir-632-p5_2ss7GT18GT	0	0	0	0	0	0	2	0	0	0	0	0	0	0	0	0	0	0	0	0	0	0	0	0
477	TTTTGTTTCTGTTTCCT	rno-mir-632-p3_1ss7GT	0	2	0	0	0	0	0	0	0	0	0	0	0	0	0	0	0	0	0	0	0	0	0	0
478	TCAGCAGTGTCAGAGGTG	rno-mir-6319-p5_2ss11TC17AT	0	0	0	0	0	0	0	2	0	0	0	0	0	0	0	0	0	0	0	0	0	0	0	0
479	CTCAGGCCTGTGCCCAAGGACT	rno-mir-6318-p5	13	22	15	0	7	8	3	9	13	17	6	3	0	0	2	2	0	0	10	4	0	6	0	2
480	TGTGTGCTCTGACTGTAA	rno-mir-6316-p5_2ss15TG17CA	0	0	0	0	0	2	0	0	0	0	0	0	0	0	0	0	0	0	0	0	0	0	0	0
481	TCACGTGCCCTTCCTGTCCTCT	rno-mir-6315-p3	0	0	6	0	3	0	0	0	6	0	3	2	0	0	0	0	0	2	0	2	0	0	0	0
482	AGAGGCACTGAAAGA	rno-mir-6314-p5_1ss12CA	2	0	2	0	2	0	0	0	0	0	0	0	0	0	0	0	0	0	0	0	0	0	0	0
483	GCAGCATTCAGGCCTGGCTC	rno-mir-6314-p3_3ss6CA17TG19AT	0	0	0	0	0	0	2	0	0	0	0	0	0	0	0	0	0	0	0	0	0	0	0	0
484	CTGCATCTGTGTGTCTAAC	rno-mir-6216-p3_2ss15AC18GA	0	0	0	0	0	0	0	0	0	0	0	0	2	0	0	0	0	0	0	0	0	0	0	0
485	GTGCTGTAGCACACT	rno-mir-6215-p5_1ss6CG	0	0	0	0	0	0	0	0	0	0	0	0	0	0	0	0	0	0	0	0	0	0	0	4
486	AGGGGCGGCGGGGCC	rno-mir-484-p5_1ss6GC	2	0	0	0	0	0	0	0	0	0	0	2	3	0	2	4	0	3	7	3	0	0	0	3
487	ATACTTACACACACACT	rno-mir-466b-1-p3_2ss5AT17AT	0	6	2	2	4	4	0	0	5	6	0	0	0	0	0	0	0	0	2	0	0	0	0	0
488	AGTAGATTGTATAGTTA	rno-mir-3596d-p5_1ss2AG	4	4	1	0	0	0	0	0	2	0	0	0	0	0	0	0	0	0	0	0	0	0	0	0
489	AGTCTGGGGAAAGCT	rno-mir-3596c-p5_1ss3CT	2	0	0	0	0	0	0	0	0	0	0	0	0	0	0	0	0	0	0	0	0	0	0	2
490	AAGGAAGGCAGCAGG	rno-mir-3596b-p5_1ss4AG	0	0	0	0	2	0	0	0	0	0	0	0	0	0	0	0	0	0	0	0	0	0	0	0
491	CAGGGGTGAAGCTGC	rno-mir-3593-p5_1ss6TG	0	0	0	0	0	0	0	0	0	0	0	0	0	0	0	0	0	0	0	0	0	0	0	2
492	TCCAGAGTGGAAACC	rno-mir-3589-p5_1ss8AT	0	0	0	0	0	0	0	0	0	0	0	0	0	0	0	0	0	0	0	0	0	0	0	2
493	ATGCTACTAAAATACCA	rno-mir-3588-p5_2ss4TC17TA	2	0	2	0	0	0	0	0	0	0	0	0	0	0	0	0	0	0	0	0	0	0	0	0
494	TGGATCCCCGCAGCCC	rno-mir-3584-p3_1ss16TC	0	0	2	0	0	0	0	0	0	0	0	0	0	0	0	0	0	0	0	0	0	0	0	2
495	AGTAGAGGAAAGTTC	rno-mir-3580-p3_1ss8TG	0	0	0	0	0	0	0	0	0	0	2	0	0	0	0	0	0	0	0	0	0	0	0	0
496	AAAAGAGCAAAGACA	rno-mir-3576-p3_1ss12CG	0	0	0	0	0	0	2	0	0	0	0	3	0	0	0	0	0	0	0	0	0	0	0	2
497	TACCCGGCAGTTTTAGAG	rno-mir-3575-p5_1ss12AT	0	2	3	0	0	0	0	0	0	0	0	0	0	0	0	0	0	0	0	0	0	0	0	0
498	GACGAGGGCTCCTACC	rno-mir-3575-p3_1ss10CT	2	0	2	0	2	0	0	0	0	0	0	0	0	0	0	0	0	0	0	0	0	0	0	7
499	CGGACATTACCTGCC	rno-mir-3571-p5_1ss13AG	0	0	0	0	0	0	0	0	2	0	0	0	0	0	0	0	0	0	0	0	0	0	0	0
500	TCGGGACCTGAGTCTGCGCT	rno-mir-3569-p3	0	0	0	0	2	3	0	0	0	0	0	0	0	0	0	0	0	0	0	0	0	0	0	2
501	CTGTATCCCTCGAGC	rno-mir-3564-p5_1ss12AG	0	0	0	0	0	0	0	0	0	0	0	0	3	0	0	0	0	0	0	0	0	0	0	0
502	GCTGTGATTTGGGGCT	rno-mir-3562-p5_1ss16AT	0	0	0	0	0	0	0	0	0	2	0	0	0	0	0	0	0	0	0	0	0	0	0	0
503	CTAGAGCCTGAGACC	rno-mir-3562-p3_1ss14TC	5	2	4	0	7	2	4	0	2	2	0	2	0	0	0	2	0	0	2	2	0	0	0	0
504	AGGGCAGACACTCAGAACCTGAGACACT	rno-mir-3561-p5_2ss26TA27GC	0	2	0	0	4	0	2	0	0	2	0	0	0	0	0	0	0	0	0	0	0	0	0	4
505	CAGAACCCGAGGCAGCA	rno-mir-3561-p3_1ss8TC	2	2	0	0	0	0	0	0	0	0	2	0	0	0	0	0	0	0	0	0	0	0	0	0
506	AGACCAGGCTGACCT	rno-mir-3560-p5_1ss11TG	0	2	0	0	0	0	0	0	0	2	0	0	0	0	0	0	0	0	0	0	0	0	0	3
507	CCTACAAATAGCATTTTG	rno-mir-3560-p3_2ss2AC18CG	0	0	2	0	0	0	0	0	0	2	0	0	0	0	0	0	0	0	0	0	0	0	0	0
508	TGGCACTGTCACCCCC	rno-mir-3557-p3_1ss5TA	2	0	2	0	0	2	0	0	7	0	0	0	0	0	0	2	0	2	0	0	0	0	0	4
509	GGCCAGTACTGTCTT	rno-mir-3551-p5_1ss3GC	0	0	0	2	0	0	0	0	0	0	0	0	0	0	0	0	0	0	0	0	0	0	0	0
510	ACTGGTACTTTGTAG	rno-mir-3549-p5_1ss6GT	0	0	2	0	0	0	0	0	0	0	0	0	0	0	0	0	0	0	0	0	0	0	0	0
511	GGGGCCCGGGACAGTACTC	rno-mir-3547-p5_2ss13TA18CT	0	2	0	0	4	2	0	0	2	0	0	0	0	0	0	0	0	0	2	0	0	0	0	0

512	ACGATAGCTACTCTGAT	rno-mir-3546-p5_2ss10CA17CT	0	0	2	0	0	0	0	0	0	0	0	0	0	0	0	0	0	0	0	0	0	0	0	0
513	CTCTCCCTCCCCTTC	rno-mir-3541-p3_1ss13CT	4	3	7	0	6	2	0	0	4	4	7	4	2	0	0	0	0	0	0	0	0	0	0	5
514	TTGGGGGTGGGGGCT	rno-mir-349-p5_1ss8AT	2	0	0	0	0	0	0	0	0	0	0	0	0	0	0	0	0	0	0	0	0	0	0	0
515	AGTCTTCTCCCCCCA	rno-mir-349-p3_1ss9TC	0	0	0	0	0	0	0	0	0	0	0	0	0	0	0	2	0	0	0	0	0	0	0	2
516	CCTGGCTAGATTCCAGGTACCAATTGGTACCTGA	rno-mir-344a-1-p5_1ss24CT	0	0	1	0	0	0	0	0	0	0	0	0	0	0	0	0	0	0	0	0	0	0	0	0
517	CTCGGAGCCTGGGGCA	rno-mir-328-p5	4	2	0	0	0	2	3	0	2	3	0	2	0	0	0	0	0	0	0	2	0	0	0	3
518	CTCATCTGTCTGTGGGGCT	rno-mir-326-p5	6	13	0	0	4	5	2	0	4	9	5	5	0	0	0	0	0	0	3	3	0	2	0	0
519	TCCAAGTTTCAGGAGCT	rno-mir-31b-p3_1ss17AT	0	0	0	0	0	0	0	0	0	2	0	0	0	0	0	0	0	0	0	0	0	0	0	4
520	CTGCCTGTCTCTGCCT	rno-mir-3120-p5_1ss11GC	0	7	0	0	2	2	0	0	0	4	0	0	0	0	0	0	0	0	0	0	0	0	0	0
521	CAACTCTGTCAATCC	rno-mir-3120-p3_1ss11CA	2	0	8	0	0	0	0	0	0	2	0	2	0	0	0	0	0	0	0	2	0	0	0	0
522	CTGAGCGATCGATCCCCATC	rno-mir-3099-p5	0	0	0	0	0	2	0	0	0	0	0	0	0	0	0	0	0	0	0	0	0	2	0	0
523	TGCCTCTTGTCTTCTGC	rno-mir-3075-p3_2ss2AG17TC	0	0	0	0	0	0	0	0	0	3	0	0	0	0	0	0	0	0	0	0	0	0	0	0
524	AGGACTGCCTGGGCAGAGTC	rno-mir-3065-p3_2ss6GT17CA	2	0	0	0	3	0	0	0	0	2	0	2	0	0	0	2	0	0	0	0	0	0	0	2
525	CCTAAAGGATCCTCATT	rno-mir-2985-p5	0	0	0	0	0	0	0	0	0	2	0	0	0	0	0	0	0	0	0	0	0	0	0	0
526	AAAGTGCTTCCATTTTGTGTGT	rno-mir-294-p3	0	2	0	0	0	2	0	0	2	0	0	0	0	0	0	0	0	0	0	0	0	0	0	0
527	CTTCAAAGTGGAGGCCCCCT	rno-mir-291b-p5_2ss2AT18TC	0	0	0	0	0	0	0	0	0	1	0	0	0	0	0	0	0	0	1	0	0	0	0	0
528	CACTTTGTCTGCCACT	rno-mir-291a-p3_1ss9GC	0	0	0	0	0	0	0	0	0	0	0	2	0	0	0	0	0	0	0	0	0	0	0	2
529	ATGGGATCTGATGCC	rno-mir-23a-p5_1ss8TC	2	2	0	0	0	3	4	0	2	2	0	0	0	0	0	0	0	0	2	0	0	2	0	7
530	TTTGCTGCTCTCTACT	rno-mir-20b-p3_1ss9AT	8	0	5	2	3	8	6	2	5	4	0	3	0	2	0	0	0	4	0	4	0	0	0	2
531	CCACCTCCTGCACCT	rno-mir-208b-p5_1ss12TA	0	0	0	0	0	0	0	0	0	0	0	0	0	0	0	0	0	0	0	0	0	0	0	2
532	AGAAGGCATGGAGCT	rno-mir-205-p3_1ss3GA	0	0	0	0	0	0	2	0	0	0	0	0	0	0	0	0	0	0	0	0	0	0	0	0
533	TTCTCTGTGGATCTGGA	rno-mir-202-p5_2ss5TC17TA	0	0	2	0	0	0	0	0	0	0	0	0	0	0	0	0	0	0	0	0	0	0	0	0
534	CAAAAGAGGTATAGC	rno-mir-202-p3_1ss2TA	0	0	0	0	0	0	2	0	0	0	0	0	0	0	0	0	0	0	0	0	0	0	0	0
535	ATGGCTTTCTGAACACAAC	rno-mir-196c-p3_1ss1CA	2	2	0	0	0	0	0	0	0	0	0	0	0	0	0	0	0	0	0	0	0	0	0	0
536	GCTGGTTGGCATTCTGGGCCTGGTTCATGCC	rno-mir-1949-p5	0	0	0	0	0	0	0	0	0	0	0	0	0	0	0	0	0	0	0	2	0	0	0	0
537	GGAGACAACAGCTGCT	rno-mir-191b-p3	0	0	0	0	0	0	0	0	0	0	0	0	0	0	0	0	0	0	0	0	0	0	0	2
538	ATGAGCCAACTAAATGT	rno-mir-190b-p3_1ss5AG	0	0	0	0	0	0	0	0	0	0	0	0	0	0	0	0	0	0	0	0	0	0	0	2
539	CTAGCTGCCTGAGGC	rno-mir-182-p5_1ss10GT	0	0	2	0	0	0	0	0	0	0	0	0	0	0	0	0	0	0	0	0	0	0	0	0
540	AACTCACTGGTCAATGAATGCCT	rno-mir-181b-2-p3_2ss22AC23AT	0	0	4	0	0	2	0	0	0	0	0	0	0	0	0	0	0	0	0	0	0	0	0	0
541	TCTTCCTGAGATGTT	rno-mir-133b-p5_1ss12GT	0	0	0	0	0	0	0	0	0	0	0	0	0	0	0	0	0	0	0	0	0	0	0	2
542	ACATTGTCACAGATTC	rno-mir-10b-p3_1ss5AT	0	0	0	0	0	2	0	0	0	0	0	0	0	0	0	0	0	0	0	0	0	0	0	0
543	TTCGGTTATCTAGCTTTATGA	rno-miR-9b-5p_R+2	2	6	13	0	4	4	2	0	2	1	0	1	0	1	2	0	0	0	1	1	0	1	1	0
544	AGGTTGGGATTTGTCGCAATGCT	rno-miR-92a-1-5p	2	3	10	0	0	0	2	5	3	3	2	0	0	0	0	0	0	0	0	2	0	0	0	0
545	TATTCAGATTGGTGCCGGTCA	rno-miR-871-5p_R-3	0	0	0	0	0	0	0	2	0	0	0	0	0	0	0	0	0	0	0	0	0	0	0	0
546	TGACTGGCACCATACTGGATAAT	rno-miR-871-3p_R+1	0	3	0	0	0	0	0	0	0	0	0	0	0	0	0	0	0	2	0	0	0	2	0	0
547	ACGGAGAGTCTTTGTCACTCA	rno-miR-802-3p_R-2	2	0	7	2	0	2	0	0	3	4	0	0	0	0	0	0	0	0	3	0	0	0	0	0
548	CAACAAGTCACAGCCAGCCTCA	rno-miR-7b-3p	12	9	15	0	9	3	7	8	12	7	6	7	4	3	2	0	0	0	3	2	0	0	2	3
549	TGGTTGACCAGAGAGCACACG	rno-miR-758-5p	2	0	0	0	0	0	0	0	0	0	0	2	0	0	0	0	0	0	0	0	0	0	0	3
550	TGTTCAGACTGGTGTCCATCAT	rno-miR-743b-5p	0	2	0	0	2	0	0	0	0	0	0	0	0	0	0	0	0	0	0	0	0	0	0	0
551	GAAAGACGCCAAACTGGGTAGA	rno-miR-743a-3p	0	0	0	0	0	0	0	0	0	0	0	0	0	0	0	0	0	0	0	0	0	3	0	0
552	GTGAGTGGGGTGGTTGGCATG	rno-miR-702-5p	3	0	2	0	0	3	0	0	0	0	0	0	0	0	0	0	0	0	0	0	0	0	0	2
553	GAACTGTGAGGTGACTCTTGGT	rno-miR-679-5p_1ss2GA	0	7	3	0	2	0	0	0	2	2	0	0	0	0	0	0	0	0	0	2	0	0	0	5
554	GGCTGCAGCGTGATCGCCTGCTCT	rno-miR-666-3p_R+1	10	7	28	0	2	2	4	2	13	6	2	4	0	2	0	0	0	0	0	3	2	0	0	6
555	AGGGGCCTCTGCCTCTATCCAGG	rno-miR-665-5p_R-3	0	2	0	0	0	0	0	0	0	0	0	0	0	0	0	0	0	0	0	0	0	0	0	0
556	TTGAAACATTCTCTACTGAACC	rno-miR-653-5p_R+1	2	8	27	2	3	3	2	3	4	2	2	2	0	3	0	0	0	2	0	2	0	3	0	0
557	CAACCCTAGGAGGGGGTGCCATTC	rno-miR-652-5p_L-1R+2	4	10	22	3	2	11	4	2	14	12	0	6	0	0	0	0	0	0	4	0	0	0	0	2
558	AATGTGACTCAGCTATCTGAAC	rno-miR-6329_R-1	4	12	8	0	6	7	2	5	9	6	7	9	0	0	0	0	0	0	3	7	0	0	0	6
559	AGGCCTGCTCTGAGCCCCCGCCT	rno-miR-6328_R+2	8	17	10	4	3	8	6	3	5	8	3	8	2	0	0	0	0	0	6	7	0	2	2	4
560	CTGGTCTGTATAGGGAC	rno-miR-6326_R-6_1ss11AT	0	0	6	0	0	2	0	0	2	0	0	0	0	0	0	0	0	0	0	0	0	0	0	0

561	TCAGTAGGCCAGACAGCAAGC	rno-miR-6324_R-2	0	2	5	2	0	2	0	0	4	0	0	0	0	0	0	0	0	0	0	0	0	0	0	0
562	TGGCACTCCATCTCT	rno-miR-6323_R-10_1ss4AC	4	2	0	0	2	0	0	0	2	2	2	0	0	0	0	0	0	0	0	0	0	0	0	4
563	TCATTCTCGCTGCTCTGGAGT	rno-miR-6319	0	11	9	2	5	6	7	3	5	6	0	3	2	0	0	2	2	2	0	5	0	0	0	2
564	GTGTCTGTCTCTGTG	rno-miR-6316_L-1R-7_1ss2AG	0	0	0	0	0	0	0	0	0	0	0	1	0	0	0	0	0	0	0	0	0	0	0	0
565	TCTGGACAGGACAGGCCCTGAGC	rno-miR-6315	6	14	28	3	11	10	3	0	9	9	6	4	2	0	0	0	0	0	2	2	2	0	0	2
566	TATACACAGAGGCAGGAGGAGA	rno-miR-6216_R-1_1ss1GT	0	5	0	0	3	2	0	0	0	0	0	2	0	0	0	0	0	2	0	0	0	0	0	4
567	GCGGTGATGCCGATGGTGCGAGC	rno-miR-598-5p_R+1	0	0	4	0	2	0	0	0	0	0	2	0	0	0	0	2	0	0	0	0	0	0	0	2
568	TCATCACGTGGTGACGCAACAT	rno-miR-592-3p	0	0	0	0	0	3	0	0	0	0	0	0	0	0	0	0	0	0	0	0	0	0	0	0
569	TCACTTCAGGATGTACCACCC	rno-miR-547-5p_R-1	2	0	2	0	2	0	0	2	2	0	0	0	2	0	0	0	0	2	0	2	0	0	0	2
570	CATGCCTTTTGCTCTGCACTCA	rno-miR-511-5p_R+1	0	0	2	0	0	2	2	0	0	2	0	0	0	0	0	0	0	0	0	0	0	0	0	0
571	AATGTGTAGCAAAAGACAGGAT	rno-miR-511-3p_R+1	2	5	0	0	0	4	2	2	3	4	3	0	0	0	0	0	0	0	0	0	0	0	0	0
572	TACTCCAGAATATGGCAATCATG	rno-miR-509-5p	0	2	0	0	0	0	0	0	0	0	0	0	0	0	0	0	0	0	0	2	0	0	0	0
573	AGGGAGAGCAGGGCAGGGTTTC	rno-miR-504-3p	0	4	0	0	4	0	2	2	4	3	0	0	0	0	0	0	0	0	0	0	0	0	2	0
574	TAGCAGCGGGAACAGTACTGCAG	rno-miR-503-5p	4	12	15	2	4	2	2	3	2	6	3	2	3	3	0	0	2	2	7	2	0	0	0	2
575	AATCCTTTGTCCCTGGGTGAAAATGC	rno-miR-501-5p_R+6	0	0	2	0	0	0	2	0	0	0	0	0	0	0	0	0	0	0	0	0	0	0	0	0
576	AATCCTTGCTATCTGGGTGCTTAGT	rno-miR-500-5p_R+1	0	3	0	0	3	4	5	2	2	0	2	0	2	0	0	2	0	0	0	0	0	0	0	0
577	CGGCACTGTGGCCACGTCCAAACCACACTGTGGTGTTAGA	rno-miR-497-3p_L+17	0	4	8	0	0	7	2	0	2	4	3	0	0	0	0	0	0	0	0	0	2	2	0	0
578	AGGTTGTCCATGGTGTGTTCA	rno-miR-496-5p_R+1	0	2	0	0	0	0	0	0	0	3	0	0	0	0	0	0	0	0	0	2	0	0	0	2
579	GAAGTTGCCCATGTTATTTTTC	rno-miR-495-5p_R-1	0	3	12	0	0	0	0	9	4	2	0	0	0	0	0	0	0	0	0	0	0	2	0	2
580	AGGTTGTCCGTGTTGTCTTCTCT	rno-miR-494-5p_R+1	0	0	3	0	0	0	0	0	2	0	0	0	0	0	0	0	0	0	0	0	0	0	0	0
581	ATGACATCACATATATGGCAGC	rno-miR-489-3p	0	0	2	0	0	0	0	0	0	2	0	0	0	0	0	0	0	0	0	0	0	0	0	0
582	AAGACGGGAGAAGAGAAGGGAGT	rno-miR-483-5p_R+1	0	4	4	0	0	0	0	0	0	6	0	0	0	0	0	0	0	0	0	0	0	0	0	0
583	TACGTAGTATAGTGCTTTTCAC	rno-miR-471-5p	0	3	0	0	0	0	0	0	0	0	0	0	0	0	0	0	0	0	0	0	0	4	0	0
584	TGAAAGGTGCCATACTATGTAC	rno-miR-471-3p_R+2	0	2	0	0	0	0	0	0	0	0	0	0	0	0	0	0	0	0	0	0	0	2	0	0
585	ATGTGTGTGTGTATGTCCAT	rno-miR-466b-5p_L-1R-1	1	0	0	0	1	0	2	0	0	0	0	0	0	0	0	0	0	0	0	0	0	0	0	0
586	ATTGGGAACATTTTGCATAAA	rno-miR-450a-3p_L-1R+1	2	0	0	0	0	0	0	2	0	0	0	0	0	0	0	0	0	0	0	0	0	0	0	0
587	TGGCAGTGTATTGTTAGCTGGT	rno-miR-449a-5p	4	5	8	0	7	0	2	2	4	8	0	0	0	0	0	0	0	4	0	0	2	2	0	6
588	GAACATCCTGCATAGTGCTGCC	rno-miR-448-5p_L+1R-1	7	8	41	4	3	4	7	6	8	2	5	5	0	0	0	0	0	0	2	0	2	5	0	11
589	GGCCTCATTAAATGTTTGTTGA	rno-miR-421-5p_R+1	0	14	19	0	4	12	6	0	0	11	5	5	0	4	2	0	0	0	0	6	0	2	0	0
590	AGGTTGTCTGTGATGAGTTCG	rno-miR-410-5p	13	5	22	2	11	6	4	2	5	0	5	0	0	0	0	0	0	2	2	2	0	3	0	0
591	AGCGAGGTTGCCCTTTGTATATT	rno-miR-381-5p_R+1	16	14	31	2	10	10	2	6	14	13	3	10	5	2	3	0	0	3	0	6	0	5	0	5
592	AGAGGTTGCCCTTGGTGAATTC	rno-miR-377-5p	2	14	7	0	7	8	2	4	11	7	0	9	0	0	4	0	2	0	3	9	2	2	0	6
593	GTGGATATTCCTTCTATGGTTAT	rno-miR-376c-5p_R+2_1ss19TG	6	5	17	2	11	5	4	4	10	0	1	4	0	1	1	0	0	2	0	5	0	4	0	6
594	GAGGGACTTTCAGGGGCAGCTGTG	rno-miR-365-5p_R+7	2	0	6	0	0	0	0	0	0	0	0	0	0	0	0	0	0	0	0	0	2	0	0	0
595	CCCAGGGCAGAGCAGTGTGA	rno-miR-3594-5p_R-1	6	12	8	2	5	4	4	0	2	6	0	0	0	0	0	0	0	0	2	0	0	0	0	0
596	CTTCAACCTTAAGGGGGCCTCT	rno-miR-3593-3p_1ss22AT	0	0	2	0	2	0	0	0	0	0	0	0	0	0	0	0	0	0	0	0	0	0	0	0
597	TAGCACAATGTGAAAAGAGCTC	rno-miR-3590-3p	0	0	2	0	0	0	0	0	0	4	0	0	0	0	0	0	0	0	0	0	0	0	0	0
598	CACAAGTTAGGGTATC	rno-miR-3588_L-1R-5_1ss15CA	0	0	0	0	2	0	0	0	0	0	0	0	0	0	0	2	0	0	0	0	0	0	0	0
599	TTCACAAGAAGGTGTCTTTCATG	rno-miR-3585-5p_R+1	0	4	0	0	4	0	0	0	2	2	0	0	0	0	0	0	0	0	0	0	0	0	0	0
600	TGAACGGCCCTTGTTGTGAGGA	rno-miR-3585-3p_R+3	4	3	2	0	0	0	0	0	0	0	2	0	0	0	0	0	0	0	0	0	0	0	0	0
601	AGCATGAAGAGTTCAGATCACGT	rno-miR-3583-5p_R-1	0	0	6	0	0	4	0	2	0	0	0	5	0	0	0	0	0	0	0	0	0	0	0	0
602	CTGACTTCTCTCTTCATGCAGT	rno-miR-3583-3p_L-1R+1	7	15	10	2	7	6	0	0	4	2	0	0	0	0	0	0	0	0	0	3	0	0	2	0
603	AGGGGCAGTGATAGAAAGGAG	rno-miR-3573-5p_L-2R+1	0	0	0	0	0	0	0	0	4	0	0	0	0	0	0	0	0	0	0	0	0	0	0	0
604	CTTCCTAACTCTGCCCCCTCCCAT	rno-miR-3573-3p_L+1	0	2	0	0	2	2	0	0	0	0	0	0	0	0	0	0	0	0	0	0	0	0	0	0
605	TTACACTTGCCCTTTTTTCCCCAGT	rno-miR-3572_R+1	0	9	7	2	2	14	2	4	0	4	4	2	0	2	0	0	0	0	0	4	0	0	0	2
606	GGAGGACAGCAGACTCAGGTC	rno-miR-3569	0	2	4	0	0	4	2	0	0	0	0	0	0	0	0	0	0	0	0	0	0	0	0	2
607	ATGTAGTACTGAGTCTGTCGTG	rno-miR-3559-3p	5	7	14	0	0	2	0	4	4	0	2	0	0	2	0	0	2	0	0	0	0	0	0	0
608	CCATAGAAGTCATCCCACAGTGCC	rno-miR-3558-5p	0	2	0	0	0	0	0	0	0	0	0	0	0	0	0	0	0	0	0	0	0	0	0	2
609	ACTGTGGAGGGTTTCTATGTCT	rno-miR-3558-3p_R+2	0	5	3	0	0	3	2	0	0	2	2	0	0	0	0	0	0	0	0	3	0	0	0	0

610	TCTGACTCCAAGCCC	rno-miR-3557-5p_L-4R-3_1ss17TC	0	0	0	0	0	0	0	0	0	3	0	0	0	0	0	0	0	0	0	0	0	0	0	0
611	CCGAGCCCATCCCTGCCCTAGT	rno-miR-3550_R+1	0	4	12	0	5	5	4	3	7	3	0	0	0	2	0	0	0	0	0	2	0	0	0	7
612	ATAACCCGGGCCAAAAGCTCAC	rno-miR-3546	5	4	18	0	2	7	2	4	4	2	0	0	2	0	0	0	0	4	0	5	2	0	0	2
613	ACTCCTGCATGACGCCGTTCCC	rno-miR-3544_L-1	0	0	2	0	0	0	0	0	0	0	2	0	0	0	0	0	0	0	2	0	0	0	0	0
614	AGAAGGGCATCATATAGGAGCTGA	rno-miR-3544-5p_R+1_1ss5AG	0	0	3	0	0	0	0	0	0	0	0	0	0	0	0	0	0	0	3	0	0	0	0	0
615	AAGGCTCTTCTTTCCTTGCAGT	rno-miR-3542_R+1	4	12	3	0	9	0	2	0	6	6	2	3	0	0	0	0	0	2	2	2	0	0	0	2
616	AGGCAGTGTAATTAGCTGATTGT	rno-miR-34b-5p	0	9	7	0	7	0	4	3	8	4	9	2	0	0	0	0	0	0	3	0	2	2	2	3
617	AATCAGCAAGTATACTGCCCTA	rno-miR-34a-3p	2	4	10	0	4	3	0	0	9	0	0	0	0	0	0	0	0	0	2	3	0	0	0	0
618	AGTCAGGCTCCTGGCAGGAGT	rno-miR-344g_R-1	0	5	6	0	0	3	3	5	2	10	2	4	0	0	0	0	0	0	4	2	0	4	0	0
619	TCACCCTTCCATATCTAGTCT	rno-miR-336-5p	2	6	6	4	0	3	4	0	3	2	2	2	0	2	0	2	0	0	4	2	2	0	0	0
620	TCTATGAAGGGATGTG	rno-miR-336-3p_L-7R-1_1ss10CT	0	0	0	0	2	0	0	0	0	0	0	0	0	0	0	0	0	0	0	0	0	0	0	0
621	CTGGCCCTCTCTGCCC	rno-miR-328b-3p_R-2	8	8	7	0	1	2	2	0	4	9	4	1	0	3	1	0	0	0	7	3	0	0	0	8
622	CAATTTAGTGTGTGTGATATT	rno-miR-32-3p_L-1	0	0	4	0	5	0	0	0	0	0	2	0	0	0	0	0	0	0	0	0	0	0	0	0
623	GGAGGAGCCAAGGAC	rno-miR-3075_L-6R-3_1ss11CG	0	0	0	0	0	0	0	0	0	0	0	2	0	0	0	0	0	0	0	0	0	0	0	0
624	GATATCAGCTCAGTAGGCACCGA	rno-miR-3074_R+1	7	2	14	0	2	2	0	0	5	2	2	0	0	0	0	0	0	0	10	4	0	0	0	0
625	GTTCCTGCTGAACTGAGCCAGT	rno-miR-3074-5p	2	6	8	5	4	3	2	4	4	5	3	2	0	3	0	0	0	0	3	3	0	3	0	2
626	TCAACAAAATCACTGATGCTGGAGT	rno-miR-3065-5p_R+5	0	8	10	0	2	0	2	0	5	9	6	0	0	2	0	0	0	0	0	2	0	2	0	3
627	TGGTTTACCGTCCCACATACATA	rno-miR-299b-5p_R+3_1ss1CT	0	2	2	0	0	0	0	0	1	1	0	2	0	0	0	0	0	0	0	0	0	0	0	0
628	GTATGTGGGACGGTAAACC	rno-miR-299b-3p_L+1R+1	0	0	0	0	0	0	1	0	0	2	0	0	0	0	0	0	0	0	0	0	0	0	0	0
629	TGTTATAGTATCCCACCTACCC	rno-miR-2985	4	0	8	0	0	0	0	0	2	2	2	0	0	0	0	0	0	0	3	2	0	0	0	2
630	ATGTGTGCATGTATA	rno-miR-297_L-4R-4_1ss19GA	0	0	0	0	0	0	0	0	0	0	2	0	0	0	0	0	0	0	0	0	0	0	0	2
631	AGGGTTGGGTGGAGGCTCTCC	rno-miR-296-3p_L-1	7	7	13	0	0	3	0	2	5	5	4	0	0	3	0	0	0	0	4	0	0	0	0	2
632	AAAGTGCTACTACTTTTGGGTGT	rno-miR-295-3p_L+1	0	0	0	0	0	2	0	0	2	0	0	0	0	0	2	2	0	0	0	2	0	0	0	0
633	ACTCAAACTGGGGGCTCTTTT	rno-miR-292-5p_R-1	0	0	2	0	0	0	0	0	0	0	0	0	0	0	0	0	0	0	0	2	0	0	0	0
634	AAGTGCCGCCAGGTTTTGAGTGT	rno-miR-292-3p	0	0	2	0	0	0	0	0	3	0	0	0	0	0	0	0	0	0	0	2	0	0	0	0
635	AAAGTGCATCCATTTTGTTAGT	rno-miR-291b	0	0	4	0	0	0	0	0	2	0	2	0	0	0	0	0	0	0	0	2	0	0	0	0
636	CCTATTCTTGGTTACTTGCAC	rno-miR-26a-3p	6	12	9	6	7	9	3	0	8	0	7	0	2	0	0	0	0	2	0	3	0	4	0	4
637	CGTGTATTTGACAAGCTGAGTTGGACACT	rno-miR-223-5p_R+6	2	0	0	0	2	0	0	0	0	5	2	0	3	0	0	0	0	0	3	0	0	2	0	0
638	AGAATTGCGTTTGGACAATCAGT	rno-miR-219b-3p	0	2	2	0	0	0	0	0	0	2	0	0	0	0	0	0	0	0	0	0	0	0	0	0
639	AGATGTCCAGCCACAATTCTCG	rno-miR-219b	2	6	10	3	4	3	2	0	3	0	2	0	2	0	0	0	0	0	0	6	0	0	0	0
640	AGAGTTGCGTCTGGACGTCCCGA	rno-miR-219a-1-3p_R+1	0	8	13	4	5	8	0	3	3	4	2	2	0	0	0	0	0	3	5	3	0	0	0	0
641	AAACATGGTTCCGTCAAGCACC	rno-miR-218a-1-3p_R+1	2	5	3	2	5	3	2	2	0	1	0	0	0	4	0	2	0	2	0	1	0	2	0	0
642	TACTGCATCAGGAACTGACTGGA	rno-miR-217-5p_R+1	6	15	7	0	6	2	0	0	0	7	3	0	0	0	0	0	0	0	7	5	0	0	0	3
643	AAATCTCTGCAGGCAAATGTGA	rno-miR-216b-5p	2	3	10	2	2	0	2	2	4	5	2	2	0	0	0	0	0	0	2	0	0	0	0	3
644	CACACTTACTTGTAGAGATTCTT	rno-miR-216b-3p_R+3	0	0	0	0	0	0	0	0	2	3	0	0	0	0	0	0	0	0	0	0	0	0	0	0
645	CACAGTGGTCTCTGGGATTATG	rno-miR-216a-3p	0	0	0	0	2	0	0	0	0	0	2	0	0	0	0	0	0	0	0	0	0	0	0	0
646	ATGACCTATGATTTGACAGACA	rno-miR-215	0	6	4	0	1	6	0	0	4	10	5	4	0	0	0	0	0	0	0	0	0	2	0	4
647	TGCCTGTCTACACTTGCTGTGC	rno-miR-214-5p	0	3	7	0	2	0	0	0	2	6	2	2	0	0	0	0	0	0	2	0	0	0	0	4
648	CAAAGTGCTCATAGTGCAGGTAGA	rno-miR-20b-5p_R+1	0	0	2	0	1	0	0	0	0	0	0	0	0	0	0	0	0	0	0	0	0	0	0	0
649	ACTGCATTACGAGCACTTACAG	rno-miR-20a-3p_R+1	4	23	37	6	15	10	2	4	11	0	2	0	0	2	0	0	0	2	4	2	0	2	0	2
650	TTGACCCGGGTTATA	rno-miR-208a-5p_L-6R-1_1ss10GA	0	0	2	0	0	0	0	0	0	0	0	0	0	0	0	0	0	0	0	3	0	0	0	0
651	ATAAGACGAGCAAAAAGCTTGT	rno-miR-208a-3p_R+4	0	4	3	0	2	0	0	0	0	0	0	0	0	0	0	0	0	0	0	0	0	0	0	0
652	GCTGGGAAGGCAAAGGGACGT	rno-miR-204-3p_R-1	4	3	4	0	4	0	0	2	3	5	2	3	0	0	0	2	0	0	3	0	0	0	0	0
653	TTGAACTGTTAAGAACCACTGG	rno-miR-203b-3p	6	11	9	0	6	10	2	0	5	13	2	4	0	0	0	0	0	0	4	3	0	0	0	0
654	AGTGGTTCTTAACAGTTCAAC	rno-miR-203a-5p	0	3	0	0	0	0	0	0	0	0	0	0	0	0	0	0	0	0	0	0	0	0	0	0
655	TGAACAGCGCCTTTCTGTGTAGC	rno-miR-201-3p_R+1	0	10	4	0	2	0	4	0	0	4	2	3	0	0	0	0	0	0	4	0	0	0	2	2
656	AGTTTTGCAGGTTTGCATCCAGC	rno-miR-19b-1-5p_R+1	0	4	4	0	0	0	0	0	0	0	0	0	0	0	0	0	0	0	0	0	0	0	0	0
657	TAGGTAGTTTCATGTTGTTGGG	rno-miR-196a-5p	0	2	0	0	0	0	0	0	0	0	0	1	0	0	0	0	0	0	0	0	0	0	0	0
658	CCAGTGGGGCTGCTGTTATCT	rno-miR-194-3p	2	7	10	2	0	0	2	2	2	2	5	0	0	0	0	0	0	0	0	0	0	3	0	3

659	TGGGTCTTTGCGGGCAAGATGA	rno-miR-193-5p	0	2	11	0	4	0	4	0	2	5	3	2	2	2	0	0	0	2	2	2	0	0	0	2
660	AACTGGCCTACAAAGTCCCAGT	rno-miR-193-3p	0	4	5	0	5	0	7	0	0	9	0	3	2	3	0	0	2	0	5	0	2	2	0	0
661	CTGCCAGTTCCATAGGTCACAG	rno-miR-192-3p	0	4	6	0	0	0	0	0	0	0	0	0	0	0	0	0	0	0	0	0	0	0	0	0
662	GAACGAAATCCAAGTGCAGCTG	rno-miR-191b_R+1	2	2	2	0	2	2	0	2	0	2	4	0	0	0	0	0	0	0	0	0	0	0	0	0
663	CACAGAACATGCAGTGAGAACT	rno-miR-1912-3p_R+3	2	4	17	0	0	2	5	4	0	5	4	2	0	2	0	0	0	0	0	0	0	0	0	2
664	ACTGCCCTAAGTGCTCCTTCT	rno-miR-18a-3p	2	0	2	0	0	2	0	0	3	0	0	0	0	0	0	0	0	0	0	0	0	0	2	0
665	CATCCCTTGCATGGTGGAGGGC	rno-miR-188-5p_R+1	8	7	4	0	0	4	4	0	5	11	5	5	0	0	0	0	0	0	2	2	0	0	0	8
666	AGGCTACAACACAGGACCCGGG	rno-miR-187-5p_R+4	2	6	0	2	0	3	0	0	0	3	0	0	0	0	0	0	0	0	2	2	0	0	0	2
667	GCCCAAAGGTGAATTTTTTGA	rno-miR-186-3p_1ss21GA	0	4	0	0	0	0	0	3	0	0	0	0	0	0	0	0	0	0	2	0	0	0	0	2
668	CCACCGGGGGATGAATGTCAT	rno-miR-181d-3p_R+1	2	3	7	0	2	2	0	0	4	4	0	0	0	0	0	0	0	0	3	0	0	0	0	3
669	ACCAATATTATTGTGCTGCTTT	rno-miR-16-3p_R+1	3	10	15	2	6	4	2	0	0	5	6	3	0	2	0	0	0	0	0	0	0	0	0	2
670	CTGGTACAGGCCTGGGGGACA	rno-miR-150-3p_R+2	2	12	10	0	4	5	4	2	8	12	2	3	2	2	0	0	0	2	0	9	0	0	0	0
671	TGGAAACACTTCTGCACAAACT	rno-miR-147-5p_R-2_1ss9TC	0	3	2	0	0	0	2	0	4	2	2	0	0	0	2	0	0	0	3	0	0	2	0	3
672	GTGTGCGGAAATGCTTCTGCTA	rno-miR-147	0	8	4	0	2	0	0	2	2	2	4	3	0	0	0	0	0	0	0	0	0	0	0	2
673	TGTAGGGCTAAAAGCCATGGGC	rno-miR-135b-3p_L-1R+1	0	25	11	4	4	7	2	0	6	16	8	8	0	0	2	0	0	0	8	2	0	6	2	9
674	AGCTGGTAAAATGGAACCAAAT	rno-miR-133a-5p	0	3	5	0	0	2	2	0	0	0	0	0	0	0	0	0	0	0	2	0	0	0	0	3
675	CAGTGCAATGATGAAAGGGCA	rno-miR-130b-3p_R-1	0	6	0	0	2	0	0	2	0	5	0	0	0	0	0	0	0	0	4	4	0	0	0	0
676	GCTCTTTTCACATTGTGCTACT	rno-miR-130a-5p	8	5	8	2	2	6	2	4	7	2	6	3	2	0	0	2	0	0	4	0	0	5	0	6
677	ACGTTGGCTCTGGTGGTGATGT	rno-miR-1306-3p_L-1R+1	0	0	8	0	2	2	0	0	0	4	0	0	0	0	0	0	0	0	0	0	0	0	0	0
678	AGGAGGGAGGGGATGGGCCAAGTTC	rno-miR-1249-5p	0	0	0	2	2	0	0	0	0	0	0	0	0	0	0	0	0	0	0	0	0	0	0	0
679	TGGAGTGTGACAATGGTGTTTGT	rno-miR-122-5p_R+1	6	17	35	6	18	13	6	9	12	7	0	4	0	0	0	0	0	0	3	0	0	3	0	2
680	TCCGAGGCTCCCCACCACACCCTG	rno-miR-1188-3p_L+2R+5	0	5	9	2	4	0	2	0	2	2	0	2	0	0	2	0	0	0	0	2	0	0	0	4
681	CAAATTCGTATCTAGGGGAAT	rno-miR-10a-3p_R-1	0	0	2	0	0	0	0	0	0	0	0	0	0	0	0	0	0	0	0	0	0	0	0	0
682	AGCTTCTTTACAGTGTTGCCTTG	rno-miR-107-5p_R-1	2	3	9	3	6	9	2	0	10	0	0	0	0	0	0	0	0	0	0	0	0	0	0	0
683	TCAAGTGCTCAGATGTCTGTGGT	rno-miR-105_L+1	0	0	0	0	0	0	0	0	0	2	0	0	0	0	0	0	0	0	0	0	0	0	0	0
684	GGCTTCTTTACAGTGCTGCCTTG	rno-miR-103-1-5p_R-1	0	2	1	0	0	2	0	0	0	2	0	0	0	0	0	0	0	0	0	0	0	3	0	0
685	TCGGTTATCATGGTACCGATGCTGTAGATCTGAAAGGT	rno-miR-101b-5p_R+16	0	2	0	0	0	0	0	0	0	0	2	0	0	0	0	0	0	0	0	0	0	0	0	0
686	ACAAGCTTGTGTCTATAGGTAT	rno-miR-100-3p_L+1R+2	8	9	23	0	4	7	2	4	9	6	11	11	3	4	0	0	0	0	2	4	0	0	2	2
687	CTGTACAGCCTCCTAGCTTTCC	rno-let-7a-2-3p	11	18	33	3	4	8	6	9	11	2	5	9	0	2	0	0	0	4	2	0	0	2	2	0
688	TCCGAGCCTGGGTCTCCCTCT	cgr-miR-615-3p_R-1	6	8	13	2	6	0	7	0	0	6	3	0	2	0	0	4	0	2	2	0	0	0	0	9

Supplemental Table 2. List of miRNAs identified from RNA-Seq analysis.

ABCA1
ABCC5
ACOX1
ACVR1B
ACVR2B
AGO1
AHSA2
AKAP13
ALS2
AMN
ANGEL2
ANKRD13C
ANKRD52
AP1G1
APLNR
ARHGAP29
ARHGAP35
ARIH2
ATG14
ATP1B3
ATP2B2
ATXN1L
ATXN7
BCAM
BCL7A
BEND3
BMPR2
BTBD9
BTRC
C10orf105
C18orf25
C2orf68
C6orf120
CABP7
CACUL1
CBL
CBX5
CCDC120
CCDC88C
CCNJ

CCNY
CDCA7L
CECR2
CELSR1
CHN2
CLCN3
CLOCK
CREBRF
CSDC2
CSNK1D
DDB2
DDX6
DLC1
DLGAP3
DNAH3
DST
EPB41L1
ERBB4
ETS2
ETV6
EVX2
FAM133B
FAM222B
FER
FP15737
GCNT2
GIT1
GJA5
GPR124
GPR180
GPR63
GPRC5A
GRIK3
GSK3B
HAPLN1
HDLBP
HIF1A
HIP1R
HMCN1
HSPBAP1

IKBKB
INO80D
ITFG3
ITGA3
ITGA8
KANK2
KIAA0226
KIAA1324L
KIAA2018
KLHL23
KPNA4
LCOR
LIN7C
LRP4
M6PR
MAPK4
MAPK8
MARK4
MAX
MGAT3
MICAL3
MIER3
MINK1
MIOS
MTMR3
MYEF2
MYH9
MYO1C
MYO5A
MYRF
NAA15
NCOA2
NCSTN
NLK
NPTX2
NUFIP2
ONECUT2
OSR1
PAN3
PAOX

PDE7A
PHF21B
PLXNA2
PLXND1
PNPLA6
PODXL
POGK
PPARGC1A
PPL
PPM1B
PPP1R12A
PPP1R1A
PPP1R2
PPP2R2D
PPP6C
PRDM16
PRPF40A
PTPN4
PTPN9
PURA
RAB21
RAD23B
RAD54L2
RANBP10
RB1
RBM23
RBM47
RGMA
RGP1
RORB
RPS6KA5
RSF1
RUNX1T1
SH3PXD2A
SHOC2
SIRT1
SLC25A23
SLC25A37
SMARCD1
SNN

SORL1
SOS2
SOX6
SRGAP3
SRSF1
ST6GAL1
STON2
STRADB
STRN
SULF1
TAB3
TANC2
TAOK1
THUMPD3
TMEM135
TMEM178B
TMEM19
TMEM63B
TMEM97
TMOD1
TNPO1
TRAF3
TST
UBE2Q1
UBN2
UCP3
UHMK1
USP37
VGLL2
VPS26A
WDR55
WIPI2
WNT2
WNT3A
WNT7A
WNT7B
YAF2
ZBTB20
ZBTB37
ZBTB42

ZBTB5
ZCCHC2
ZFP91
ZIK1
ZNF225
ZNF226
ZNF512B
ZNF516
ZNF740

Supplemental Table 3. Common targets of miR-199a and miR-214 idenified by bioinformatics analysis.
